# B-Ring-Aryl Substituted Luotonin A Analogues with a New Binding Mode to the Topoisomerase 1-DNA Complex Show Enhanced Cytotoxic Activity

**DOI:** 10.1371/journal.pone.0095998

**Published:** 2014-05-15

**Authors:** Víctor González-Ruiz, Irene Pascua, Tamara Fernández-Marcelo, Pascual Ribelles, Giulia Bianchini, Vellaisamy Sridharan, Pilar Iniesta, M. Teresa Ramos, Ana I. Olives, M. Antonia Martín, J. Carlos Menéndez

**Affiliations:** 1 Sección Departamental de Química Analítica, Facultad de Farmacia, Universidad Complutense, Madrid, Spain; 2 BIOHET (Biologically Relevant Heterocycles) group, Facultad de Farmacia, Universidad Complutense, Madrid, Spain; 3 Departamento de Bioquímica y Biología Molecular II, Facultad de Farmacia, Universidad Complutense, Madrid, Spain; 4 Instituto de Investigación Sanitaria del Hospital Clínico San Carlos, Madrid, Spain; 5 Departamento de Química Orgánica y Farmacéutica, Facultad de Farmacia, Universidad Complutense, Madrid, Spain; 6 Department of Chemical and Biotechnology, SASTRA University, Thanjavur, India; University of East Anglia, United Kingdom

## Abstract

Topoisomerase 1 inhibition is an important strategy in targeted cancer chemotherapy. The drugs currently in use acting on this enzyme belong to the family of the camptothecins, and suffer severe limitations because of their low stability, which is associated with the hydrolysis of the δ-lactone moiety in their E ring. Luotonin A is a natural camptothecin analogue that lacks this functional group and therefore shows a much-improved stability, but at the cost of a lower activity. Therefore, the development of luotonin A analogues with an increased potency is important for progress in this area. In the present paper, a small library of luotonin A analogues modified at their A and B rings was generated by cerium(IV) ammonium nitrate-catalyzed Friedländer reactions. All analogues showed an activity similar or higher than the natural luotonin A in terms of topoisomerase 1 inhibition and some compounds had an activity comparable to that of camptothecin. Furthermore, most compounds showed a better activity than luotonin A in cell cytotoxicity assays. In order to rationalize these results, the first docking studies of luotonin-topoisomerase 1-DNA ternary complexes were undertaken. Most compounds bound in a manner similar to luotonin A and to standard topoisomerase poisons such as topotecan but, interestingly, the two most promising analogues, bearing a 3,5-dimethylphenyl substituent at ring B, docked in a different orientation. This binding mode allows the hydrophobic moiety to be shielded from the aqueous environment by being buried between the deoxyribose belonging to the G(+1) guanine and Arg364 in the scissile strand and the surface of the protein and a hydrogen bond between the D-ring carbonyl and the basic amino acid. The discovery of this new binding mode and its associated higher inhibitory potency is a significant advance in the design of new topoisomerase 1 inhibitors.

## Introduction

Cancer continues to be one of the leading causes of death worldwide. According to the latest data from the International Agency for Research on Cancer, in 2012 there were 14.1 million new cancer cases, 8.2 million cancer deaths and 32.6 million people living with cancer (within 5 years of diagnosis). Cancer is no longer a disease of the developed world, with 57% of newly diagnosed cases of cancer and 65% of deaths being associated with less developed regions [1].

Cancer therapy is still founded on the pillars of surgery, radiotherapy and chemotherapy, with immunotherapy having recently entered the stage as a fourth approach [2]. Nevertheless, the development of new anticancer drugs continues to be essential in the fight against the disease [3].

Topoisomerases are present in all living organisms and are crucial for relieving torsional tension in supercoiled DNA in the course of DNA replication, transcription and reparation [4]. Topoisomerases, and topoisomerase 1 in particular, are among the most relevant anticancer targets [5], [6].

**Figure 1:**
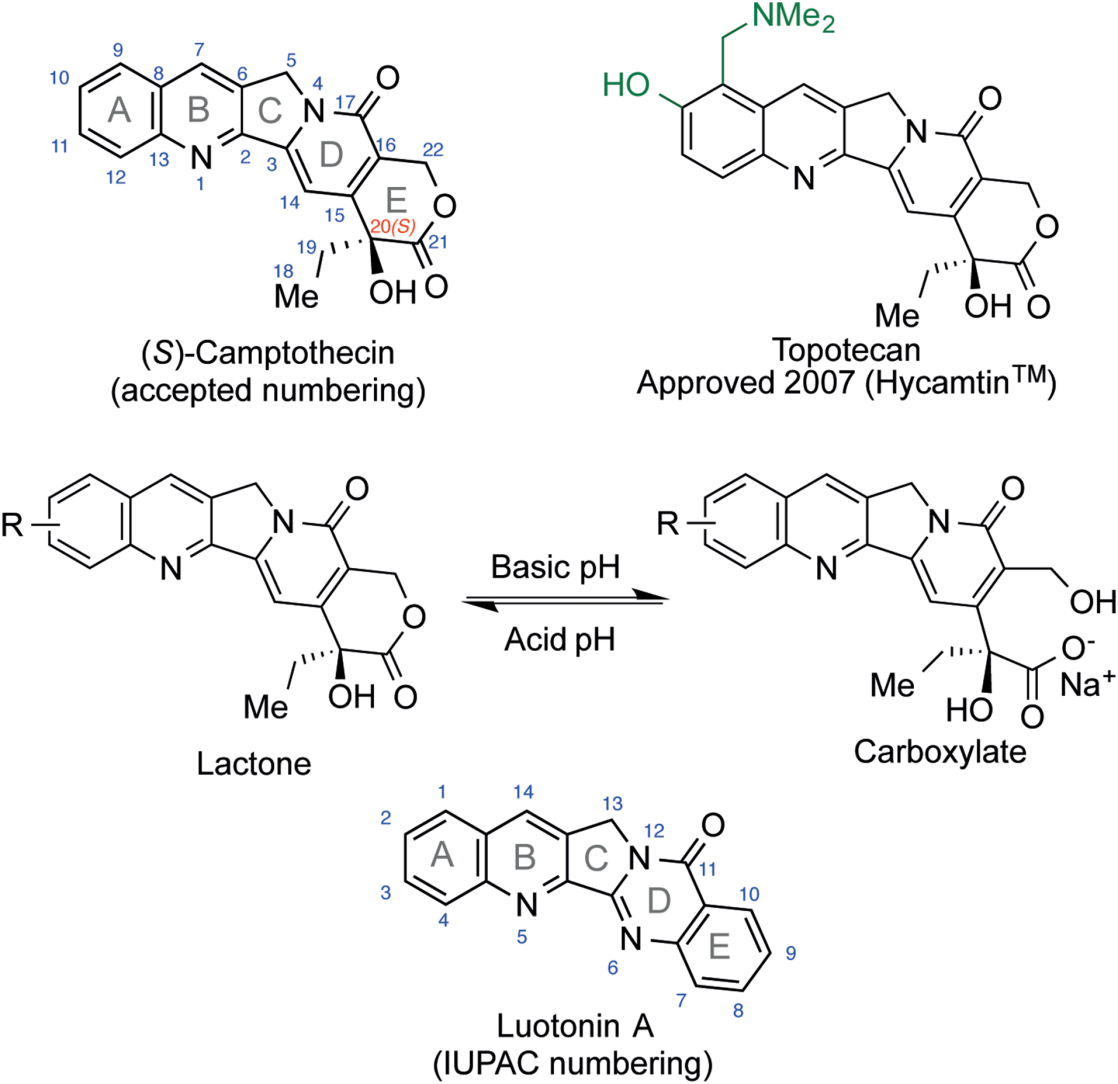
Structure of representative camptothecins and luotonin A. Camptothecin is a natural topoisomerase 1 inhibitor that has been used as a lead for the development of a family of anticancer agents in clinical use, exemplified by topotecan. Nevertheless, the camptothecins suffer severe limitations because of their low stability, which is associated with the hydrolysis of the δ-lactone moiety in their E ring that leads to an inactive carboxylate form. Luotonin A is a plant alkaloid whose structure strongly resembles that of camptothecin but lacks the lactone moiety. The discovery that luotonin A is also a topoisomerase 1 inhibitor, although less potent than the camptothecins, provided a unique opportunity for drug discovery in the anticancer area.

The camptothecins, specially irinotecan, topotecan and belotecan (Figure 1), are the main family of clinically relevant topoisomerase 1 inhibitors [7]. These compounds have a planar, pentacyclic core comprising a lactone functional group in ring E and containing a stereocenter at C-20, which must be in the *S* configuration for camptothecins to be active. Their pharmacologic target is the covalent topoisomerase 1-DNA binary complex, where they can bind non-covalently at the interphase formed between both macromolecules during the enzimatic catalytic cycle. This binding stabilizes the complex and retards its dissociation, finally leading to irreversible DNA damage and cell death [6], [8].

In spite of their widespread use, they show severe undesired effects, their main dose-limiting toxicities being myelosupression, diarrhea and bone marrow toxicity [9]. Another serious problem with the camptothecins is their low stability [10], which is due to the easy opening of its lactone E ring to give an inactive hydroxy acid form (camptothecin carboxylate) that is sequestered by seric albumin [11]. The lactone-hydroxy acid equilibrium is relevant to bladder toxicity, another problem associated to the clinical use of the camptothecins that is due to accumulation of the active δ-lactone form owing to lactonization at the acidic pH of urine [12].

Against this background, camptothecin analogues lacking the lactone ring seem attractive, but during decades this structural feature was believed to be essential for activity. The discovery of the stabilization of the human topoisomerase 1-DNA covalent binary complex by the alkaloid luotonin A, closely related to the camptothecins but having an aromatic E ring, brought about a landmark change in this paradigm [13]. Furthermore, luotonin A is achiral, a feature that can be viewed as an advantage in terms of its potential role as a lead compound in drug discovery and development because it simplifies synthetic access to new analogues and their analytical control. In summary, although the potency of luotonin is much lower than that of the camptothecins [14], it does provide an excellent opportunity for the discovery of improved topoisomerase 1 inhibitors.

The low potency of the natural luotonin A has prompted the synthesis and biological evaluation of a considerable number of analogues [15]–[18]. Nevertheless, these studies have been limited by the constraints found in known synthetic methodologies, specially in terms of A and B ring modification. Among the methods allowing access to the luotonin A framework, the Friedländer reaction between precursors corresponding to the C-D-E fragment and aromatic *o*-aminoketones or aldehydes provides the best opportunity for this type of structural manipulation. We have recently reported a protocol that improves the yields of this classical reaction by employing Ce(IV) ammonium nitrate (CAN) as a catalyst [19], and describe now its application to the synthesis of A and B ring-modified luotonin A analogues, together with studies of their activity as topoisomerase 1 poisons and cytotoxic agents.

## Materials and Methods

### 1. Synthesis

#### General experimental information

All reagents were of commercial origin (Aldrich, Fluka, Acros) and were used as received. Melting points reported in this work were measured in open capillaries. Flash chromatography was performed on silica gel (230–400 mesh) or neutral alumina having the activity grade reported in each case. ^1^H and ^13^C NMR spectra were measured at 250/300 and 63/75 MHz, respectively, with Bruker 250 and 300 MHz (Avance) instruments maintained by the CAI de Resonancia Magnética Nuclear, Universidad Complutense. The solvents used were CDCl_3_ and CF_3_CO_2_D. Chemical shifts are reported as δ values (ppm). All one- and two-dimensional NMR spectra were obtained using Bruker Topspin software. IR spectra were recorded on a Perkin-Elmer FT-IR Paragon 1000 spectrometer in film form, prepared by evaporation of a few drops of sample solution over a sodium chloride window. Combustion elemental analyses were obtained by the CAI de Microanálisis, Universidad Complutense, using a Leco CHN 932 Elemental Analyzer.

#### Synthesis of starting 2-aminophenylketones 1

2-Aminobenzaldehyde **1a**, 2-aminoacetophenone **1b**, 2-aminobenzophenone **1c** and (4-chloro-2-amino)benzophenone **1d** were purchased from Aldrich. (5-Bromo-2-amino)benzophenone **1e** was prepared according to a literature method [20].

For preparation of compounds **1f** and **1g**, a solution of the corresponding 2-aminonitrile (5 mmol, 1 eq) in anhydrous THF (10 mL) was added dropwise over a solution of 3,5-dimethylphenylmagnesium bromide (15 mmol, 3 eq) and the reaction mixture was refluxed for 2.5 h. After cooling, the reaction mixture was slowly poured onto ice water (30 mL). A 3 M aqueous solution of hydrochloric acid (21.5 mL) was then added, the mixture was stirred at room temperature for 1 h, and the organic phase was separated. The aqueous layer was extracted with ethyl ether (2×20 mL). The combined organic layers were washed with water (1×20 mL), brine (1×20 mL), dried over MgSO_4_ and filtered. The filtrate was concentrated *in* *vacuo* and the residue was either crystallized from ethanol or purified by column chromatography on silica gel eluting with petroleum ether:ethyl acetate to give compounds **1f** or **1g**.

#### 2-Aminophenyl 3,5-dimethylphenyl ketone (1f)

Prepared from 2-aminobenzonitrile (590 mg, 5 mmol) and 3,5-dimethylphenylmagnesium bromide in dry THF (30 mL, 15 mmol). Purification: chromatography on petroleum ether:ethyl acetate (10∶1, v/v). Yield: 845 mg (75%). Yellow solid, mp 65°C. ^1^H^–^NMR (CDCl_3_, 250 MHz) δ: 2.40 (s, 6H, 2×CH_3_); 6.10 (bs, 2H, NH_2_); 6.62 (t, *J* = 8.1 Hz, 1H, H-5); 6.77 (d, *J* = 8.3 Hz, 1H, H-3); 7.19 (s, 1H, H-4′); 7.27–7.35 (m, 3H, H-4, H-2′ and H-6′); 7.49 (d, *J* = 8.0 Hz, 1H, H-6). ^13^C^–^NMR (CDCl_3_, 63 MHz) δ: 21.7 (2×CH_3_); 115.9 (C-3); 117.4 (C-5); 118.8 (C-1); 127.3 (C-2′ and C-6′); 133.1 (C-6); 134.6 (C-4); 135.1 (C-4′); 138.2 (C-3′ and C-5′); 140.6 (C-1′); 151.3 (C-2); 200.1 (CO). IR (NaCl): 3473.3 (NH), 3349.1 (NH), 1615.7 (C = O) cm^−1^. Elemental analysis: Calc. for C_15_H_15_NO (M = 225.29): %C 79.97; %H 6.71; %N 6.22. Found: %C 79.58; %H 6.56; %N 6.02.

#### 2-Amino-4-methylphenyl 3,5-dimethylphenyl ketone (1g)

Prepared from 2-amino-4-methylbenzonitrile (661 mg; 5 mmol) and 3,5-dimethylphenylmagnesium bromide in dry THF (30 mL; 15 mmol). Purification: petroleum ether:ethyl acetate (3∶1, v/v). Yield: 921 mg (77%). Yellow solid, mp 125°C. ^1^H^–^NMR (CDCl_3_, 250 MHz) δ: 2.32 (s, 3H, CH_3_); 2.39 (s, 6H, 2×CH_3_); 6.10 (bs, 2H, NH_2_); 6.44–6.61 (m, 2H, H-3 and H-5); 7.17 (s, 1H, H-4′); 7.24 (s, 2H, H-3′ and H-5′); 7.38 (d, *J* = 8.2 Hz, 1H, H-6).^ 13^C^–^NMR (CDCl_3_, 63 MHz) δ: 21.7 (2×CH_3_); 22.2 (CH_3_); 116.6 (C-1); 117.4 (C- 3 and C-5); 127.1 (C-2′ and C-6′); 132.9 (C-6); 135.3 (C-4′); 138.1 (C-3′ and C-5′); 140.9 (C-1′); 145.7 (C-4); 151.6 (C-2); 199.8 (CO). IR (NaCl): 3471.3 (NH), 3346.5 (NH), 1621.0 (C = O) cm^−1^. Elemental analysis: Calc. for C_16_H_17_NO (M = 239.31): %C 80.30; %H 7.16; %N 5.85. Found: %C 79.93; %H 7.25; %N 5.55.

#### Synthesis of luotonin A and its analogues (3a–3g)

A solution of 1,2-dihydropyrrolo[2,1-*b*]quinazoline-3,9-dione (**2**) (1 or 1.5 eq), the corresponding aminophenone **1** (1 eq) and cerium(IV) ammonium nitrate, CAN (15 mol %) in ethanol (5 mL) was refluxed for the time period specified in each case. After completion of the reaction, as indicated by TLC, the mixture was cooled, diluted with dichloromethane (20 mL), washed with water (2×10 mL) and brine (1×10 mL), and dried over anhydrous Na_2_SO_4_. The solvent was evaporated under reduced pressure and the crude reaction mixture was purified by silica gel column chromatography eluting with a petroleum ether:ethyl acetate (4∶1, v/v) mixture to afford compounds **3**. Analytical samples were obtained by recrystallization from ethanol. In two of the reactions, small amounts of intermediates **4** were also isolated.

#### 13*H*-Quino[2′,3′:3,4]pyrrolo[2,1-*b*]quinazolin-11-one (luotonin A, 3a)

Prepared from compound **2** (120 mg; 0.60 mmol) and *o*-aminobenzaldehyde (**1a**) (72.7 mg, 0.60 mmol). Reaction time: 1.5 h. Yield: 112 mg (66%). Pale brown solid, mp 253°C (decomposition) (lit. [21] 252°C, decomposition). ^1^H^–^NMR (CDCl_3_, 250 MHz) δ: 5.40 (s, 2H, H-13); 7.62 (td, *J* = 8.1, 1.1 Hz, 1H, H-9); 7.74 (td, *J* = 7.9, 1.1 Hz, 1H, H-2); 7.86–7.93 (m, 2H, H-3 and H-8); 8.01 (dd, *J* = 8.0, 1.0 Hz, 1H, H-1); 8.17 (d, *J* = 7.7 Hz, 1H, H-7); 8.46–8.54 (m, 3H, H-4, H-10 and H-14).^ 13^C^–^NMR (CDCl_3_, 63 MHz) δ: 47.7 (C-13); 121.7 (C-10a); 126.9 (C-10); 127.6 (C-9); 128.4 (C-1); 128.9 (C-2); 129.2 (C-7 and C-14a); 129.8 (C-13a); 131.1 (C-3 and C-4); 132.0 (C-14); 135.0 (C-8); 149.7 (C-6a); 149.8 (C-4a); 151.6 (C- 5a); 152.9 (C-5b); 161.1 (C-11). IR (KBr): 1678.6 (C = O), 1629.6 (C = N), 1606.6 (C = N) cm^−1^. Elemental analysis: Calcd. for C_18_H_11_N_3_O (M = 285.30): %C 75.78; %H 3.89; %N 14.73. Found: %C 75.49; %H 4.03; %N 14.82.

#### 14-Methyl-13*H*-quino[2′,3′:3,4]pyrrolo[2,1-*b*]quinazolin-11-one (3b)

Prepared from compound **2** (110 mg; 0.55 mmol) and 2-aminoacetophenone **1b** (50.1 mg; 0.37 mmol). Reaction time: 24 h. Yield: 52 mg (68%). Pale brown solid. Mp: >280°C. ^1^H^–^NMR (CDCl_3_, 250 MHz) δ: 2. 85 (s, 3H, CH_3_); 5.29 (s, 2H, H-13); 7.59 (td, *J* = 7.0, 1.1 Hz, 1H, H-9); 7.69 (td, *J* = 6.8, 1.3 Hz, 1H, H-2); 7.81–7.90 (m, 2H, H-3 and H-8); 8.08–8.14 (m, 2H, H-1 and H-7); 8.41–8.49 (m, 2H, H-4 and H-10). ^13^C^–^NMR (CF_3_CO_2_D, 75.5 MHz) δ: 17.7 (CH_3_); 51.7 (C-13); 121.9 (C-10a); 124.5 (C-4); 126.7 (C-7); 127.5 (C-10); 129.7 (C-1); 132.6 (C-14a); 133.6 (C- 9); 134.1 (C-13a); 134.6 (C-2), 139.8 and 139.9 (C-3 and C-8); 142.1 (C-4a); 142.3 (C-5a); 145.5 (C-6a); 151.2 (C-5b); 158.9 (C-14); 162.4 (C-11). IR (KBr): 1673.8 (C = O), 1627.3 (C = N), 1607.2 (C = N) cm^−1^. Elemental analysis: Calcd. for C_19_H_13_N_3_O (M = 299.33): %C 76.24; %H 4.38; %N 14.04 Found: %C 75.90; %H 4.69; %N 13.73.

#### 14-Phenyl-13*H*-quino[2′,3′∶3,4]pyrrolo[2,1-*b*]quinazolin-11-one (3c)

Prepared from compound **2** (110 mg; 0.55 mmol) and 2-aminobenzophenone **1c** (73 mg; 0.37 mmol). Reaction time: 8 h. Yield: 105 mg (80%). Pale brown solid, mp>280°C. ^1^H^–^NMR (CDCl_3_, 250 MHz) δ: 5.21 (s, 2H, H-13); 7.51–7.71 (m, 7H, H-2, H-9 and Ph); 7.86–7.93 (m, 3H, H-1, H-3 and H-8); 8.18 (d, *J* = 8.2 Hz, 1H, H-7); 8.46 (dd, *J* = 8.0, 1.8 Hz, 1H, H-10); 8.52 (d, *J* = 8.5 Hz, 1H, H-4).^ 13^C^–^NMR (CF_3_CO_2_D, 63 MHz) δ: 52.5 (C-13); 122.0 (C-10a); 124.7 (C-4); 126.8 (C-7); 130.0 (C-10); 130.4 (C-1); 130.8 (C-2′ and C-6′); 132.2 (C-3′ and C-5′); 132.4 (C-14a); 133.3 (C-1′); 133.8 (C-13a); 133.9 (C-9); 134. 5 (C-4′); 135.0 (C-2); 139.9 (C-3); 140.3 (C-8); 143.3 (C-5a); 144.2 (C-4a); 145.4 (C-6a); 151.8 (C-5b); 159.9 (C-14); 162.6 (C-11). IR (KBr): 1681.3 (C = O), 1626.4 (C = N), 1466.3 (C = C) cm^−1^. Elemental analysis: Calcd. for C_24_H_15_N_3_O (M = 361.40): %C 79.76; %H 4.18; %N 11.63. Found: %C 79.42; %H 4.61; %N 11.25.

#### 2-Chloro-14-phenyl-13*H*-quino[2′,3′∶3,4]pyrrolo[2,1-*b*]quinazolin-11-one (3d)

Prepared from compound **2** (110 mg; 0.55 mmol) and 2-amino-5-chlorophenyl phenyl ketone **1d** (87.7 mg; 0.37 mmol). Reaction time: 8 h. Yield: 102 mg (71%). Pale brown solid, mp>280°C. ^1^H^–^NMR (CDCl_3_, 250 MHz) δ: 5.12 (s, 2H, H-13); 7.46–7.84 (m, 9H, H-1, H-3, H-8, H-9 and Ph); 8.03 (d, *J* = 8.1 Hz, 1H, H-7); 8.29 (dd, *J* = 7.9, 1.2 Hz, 1H, H-10); 8.36 (dd, *J* = 8.0, 1.7 Hz, 1H, H-4).^ 13^C^–^NMR (CDCl_3_, 63 MHz) δ: 47.7 (C-13); 121.5 (C-10a); 124.9 (C-1); 126.8 (C-10); 127.9 (C-9); 128.6 (C-14a); 129.0 (C-7); 129.4 (C-2′ and C-6′); 129.5 (C-13a); 129.9 (C-3′ and C-5′); 130.1 (C-4′); 131.9 (C-3); 132.6 (C-4); 133.9 (C-2); 134.9 (C-8); 135.1 (C-1′); 144.3 (C-14); 148.6 (C-6a); 149.4 (C-4a); 151.2 (C-5a); 152.8 (C-5b); 160.7 (C-11). IR (KBr): 1680.8 (C = O), 1628.1 (C = N), 1608.3 (C = N) cm^−1^. Elemental analysis: Calcd. for C_24_H_14_ClN_3_O (M = 395.84): %C 72.82; %H 3.56; %N 10.62. Found: %C 72.47; %H 4.00; %N 10.29.

#### 2-Bromo-14-phenyl-13*H*-quino[2′,3′∶3,4]pyrrolo[2,1-*b*]quinazolin-11-one (3e)

Prepared from compound **2** (300 mg; 1.5 mmol) and 2-amino-5-bromophenyl phenyl ketone (**2e**) (276 mg; 1 mmol). Reaction time: 8 h. Yield: 282 mg (64%). Brown solid, mp: 218°C (decomposition). ^1^H^–^NMR (CDCl_3_, 300 MHz) δ: 5.16 (s, 2H, H-13); 7.45–7.80 (m, 6H, H-9 and Ph); 7.82–7.92 (m, 2H, H-3 and H-8); 7.98 (s, 1H, H-1); 8.11 (d, *J* = 8.3 Hz, 1H, H-7); 8.36–8.41 (m, 2H, H-4 and H-10).^ 13^C^–^NMR (CDCl_3_, 75.5 MHz) δ: 47.7 (C-13); 121.5 (C-10a); 123.5 (C-2); 126.8 (C-10); 127.9 (C-9); 128.3 (C-1); 129.0 (C-7 and C-14a); 129.3 (C-2′ and C-6′); 129.5 (C-13a); 129.9 (C-3′ and C-5′); 130.1 (C-4′); 132.7 (C-4); 133.9 (C- 1′); 134.4 (C-3); 135.0 (C-8); 144.2 (C-14); 148.9 (C-6a); 149.5 (C-4a); 151.3 (C-5a); 152.8 (C-5b); 160.7 (C-11). IR (KBr): 1683.3 (C = O), 1627.3 (C = N), 1602.7 (C = N) cm^−1^. Elemental analysis**:** Calcd. for C_24_H_14_BrN_3_O (M = 440.29): %C 65.47; %H 3.20; %N 9.54. Found: %C 65.10; %H 3.58; %N 9.15.

#### 14-(3,5-Dimethylphenyl)-13*H*-quino[2′,3′∶3,4]pyrrolo[2,1-*b*]quinazolin-11-one (3f)

Prepared from compound **2** (300 mg; 1.5 mmol) and (2-aminophenyl) (3,5-dimethylphenyl) ketone (**1f**) (225 mg; 1 mmol). Reaction time: 8 h. Yield: 352 mg (90%). Pale brown solid, mp: 221°C (decomposition). ^1^H^–^NMR (CDCl_3_, 300 MHz) δ: 2.46 (s, 6H, 2×CH_3_); 5.19 (s, 2H, H-13); 7.10 (s, 2H, H-2′ and H-6′); 7.23 (s, 1H, H-4′); 7.57–7.67 (m, 2H, H-2 and H-9); 7.83–7.94 (m, 3H, H-1, H-3 and H-8); 8.17 (d, *J* = 7.7 Hz, 1H, H-7); 8.44 (dd, *J* = 8.0, 1.1 Hz, 1H, H-10); 8.55 (d, *J* = 8.0 Hz, 1H, H-4).^ 13^C^–^NMR (CDCl_3_, 75.5 MHz) δ: 21.8 (2×CH_3_); 47.8 (C-13); 121.7 (C-10a); 126.5 (C-1); 126.8 (C-10); 127.0 (C-2′ and C-6′); 127.7 (C-9); 128.2 (C-14a); 128.5 (C-13a); 128.6 (C-7); 129.1 (C-3); 130.7 (C-4); 131.2 (C-2); 131.3 (C- 4′); 134.5 (C-1′); 134.9 (C-8); 139.3 (C-3′ and C-5′); 145.5 (C-14); 149.8 (C- 6a); 150.4 (C-4a); 151.0 (C-5a); 153.4 (C-5b); 161.0 (C-11). IR (KBr): 1672.2 (C = O), 1626.8 (C = N), 1607.3 (C = N) cm^−1^. Elemental analysis: Calc. for C_26_H_19_N_3_O (M = 389.45): %C 80.18; %H 4.92; %N 10.79 Found: %C 79.88; %H 5.17; %N 10.38.

#### 14-(3,5-Dimethylphenyl)-3-methyl-13*H*-quino[2′,3′∶3,4]pyrrolo[2,1-*b*]quinazolin-11-one (3g)

Prepared from compound **2** (300 mg; 1.5 mmol) and (2-amino-4-methylphenyl) (3,5-dimethylphenyl) ketone (**1g**) (239 mg; 1 mmol). Reaction time: 24 h. Yield: 343 mg (85%). Grey solid, mp>280°C. ^1^H^–^NMR (CDCl_3_, 300 MHz) δ: 2.46 (s, 6H, 2×CH_3_); 2.63 (s, 3H, CH_3_); 5.16 (s, 2H, H-13); 7.09 (s, 2H, H-2′ and H-6′); 7.22 (s, 1H, H-4′); 7.45 (dd, *J* = 8.7, 1.7 Hz, 1H, H-2); 7.58 (td, *J* = 8.1, 1.1 Hz, 1H, H-9); 7.80 (d, *J* = 8.7 Hz, 1H, H-1); 7.86 (td, *J* = 7.2, 1.6 Hz, 1H, H-8); 8.14 (d, *J* = 7.7 Hz, 1H, H-7); 8.30 (s, 1H, H-4); 8.43 (dd, *J* = 8.0, 1.3 Hz, 1H, H-10). ^13^C^–^NMR (CDCl_3_, 75 MHz) δ: 21.4 (2×CH_3_); 21.8 (CH_3_); 47.4 (C-13); 121.2 (C-10a); 125.6 (C-1); 125.9 (C-14a); 126.4 (C-10); 126.6 (C-2′ and C-6′); 127.2 (C-9); 127.5 (C-13a); 128.7 (C-7); 129.7 (C-4), 130.6 (C-2); 130.8 (C-4′); 134.3 (C-1′); 134.4 (C-8); 138.8 (C-3′ and C-5′); 140.8 (C-3); 144.8 (C-14); 149.4 (C-6a); 150.3 (C-4a); 150.4 (C-5a); 153.2 (C-5b); 160.6 (C-11). IR (KBr): 1679.0 (C = O), 1626.1 (C = N), 1609.8 (C = N) cm^−1^. Elemental analysis: Calc. for C_27_H_21_N_3_O (M = 403.48): %C 80.37; %H 5.25; %N 10.41 Found: %C 79.96; %H 5.46; %N 10.22.

#### 3-(2-Acetylphenylamino)-1*H*-pirrolo[2,1-*b*]quinazolin-9-one (4b)

Prepared from compound **2** (200 mg; 1 mmol) and 2-aminoacetophenone **1b** (135 mg; 1 mmol). Reaction time: 3 h. Yield: 225 mg (75%). Yellow solid, mp 225°C (decomposition). ^1^H^–^NMR (CDCl_3_, 250 MHz) δ: 2.73 (s, 3H, CH_3_); 4.77 (d, *J* = 2.6 Hz, 2H, H-1); 6.39 (t, *J* = 2.6 Hz, 1H, H-2); 6.96 (td, *J* = 8.1, 1.8 Hz, 1H, H-4′); 7.46–7.54 (m, 3H, H-5′, H-6′ and H-7); 7.79 (td, *J* = 8.1, 1.5 Hz, 1H, H-6); 7.94–7.97 (m, 2H, H-5 and H-3′); 8.36 (dd, *J* = 8.0, 1.1 Hz, 1H, H-8); 11.52 (s, 1H, NH).^ 13^C^–^NMR (CDCl_3_, 63 MHz) δ: 28.7 (CH_3_); 49.8 (C-1); 106.6 (C-2); 116.3 (C-6′); 119.5 (C-4′); 120.8 (C-8a); 121.5 (C-2′); 126.8 (C-7 and C-8); 128.3 (C-5); 133.2 (C-3′); 133.9 (C-3); 134.5 (C-6); 135.3 (C-5′); 144.8 (C-1′); 149.7 (C- 4a); 154.6 (C-3a); 160.5 (C-9); 202.0 (COCH_3_). IR (KBr): 3444.6 (NH), 1668.1 (C = O), 1651.0 (C = O), 1601.1 (C = N) cm^−1^. Elemental analysis: Calcd. for C_19_H_15_N_3_O_2_ (M = 317.34): %C 71.91; %H 4.76; %N 13.24. Found: %C 71.51; %H 4.68; %N 12.90.

#### 3-(2-Benzoyl-4-chlorophenylamino)-1*H*-pyrrolo[2,1-*b*]quinazolin-9-one (4d)

Prepared from compound **2** (200 mg; 1 mmol) and (2-amino-5-chlorophenyl) phenyl ketone **1d** (231.7 mg; 1 mmol). Reaction time: 3 h. Yield: 102 mg (80%). Brown solid, mp: 206–207°C. ^1^H^–^NMR (CDCl_3_, 250 MHz) δ: 4.81 (d, *J* = 2.6 Hz, 2H, H-1); 6.41 (t, *J* = 2.6 Hz, 1H, H-2); 7.49–7.66 (m, 7H, H-7, H-3′, H-5′, H-6′, H-3″, H-4″ and H-5″); 7.76–7.85 (m, 3H, H-6, H-2″ and H-6″); 7.96 (d, *J* = 7.6 Hz, 1H, H-5); 7.39 (dd, *J* = 8.0, 1.2 Hz, 1H, H-8); 10.67 (s, 1H, NH).^ 13^C^–^NMR (CDCl_3_, 63 MHz) δ: 49.8 (C-1); 106.3 (C-2); 118.2 (C-6′); 120.9 (C- 8a); 123.7 (C-2′); 124.3 (C-4′); 126.9 (C-7 and C-8); 128.3 (C-5); 128.9 (C-3″ and C-5″); 130.1 (C-2″ and C-6″); 132.8 (C-4″); 133.8 (C-3); 134.4 (C-3′)*; 134.5 (C-5′)*; 134.6 (C-6′)*; 138.8; (C-1″) 143.3 C-1′); 149.6 (C-4a); 154.3 (C-3a); 160.5 (C-9); 198.2 (COPh). IR (KBr): 3445.6 (NH), 1666.4 (C = O), 1641.9 (C = O), 1609.3 (C = N) cm^−1^. Elemental analysis: Calcd. for C_24_H_16_ClN_3_O_2_ (M = 413.84): %C 69.65; %H 3.90; %N 10.15. Found: %C 69.30; %H 4.07; %N 10.05.

### 2. Pharmacological Studies

#### DNA relaxation assay

DNA relaxation assay was carried out to investigate the inhibitory effect of luotonin A derivatives on the activity of the human topoisomerase 1 enzyme. A final reaction volume of 20 µL containing 200 ng of pGEM-5Zf(+) plasmid (Promega, Madison, Wisconsin) in its native supercoiled form, the corresponding drug diluted in 1 µL DMSO for a final concentration of 10 µM, 1 unit of human topoisomerase 1 (Sigma-Aldrich, St. Louis, Missouri) and the enzyme reaction buffer (TopoGEN, Port Orange, Florida) was incubated over 15 minutes at 37°C. Lanes containing native supercoiled and fully relaxed forms were run from a mixture containing the reaction buffer and plasmid, for the first one; and the reaction buffer, plasmid and human topoisomerase 1, for the latter. After incubation, the reactions were stopped by addition of a mixture of electrophoresis loading buffer and 20% SDS. The reaction products were loaded into a 1% agarose gel (Bio-Rad, Hercules, California) and separated for 3 h at 60 V in Tris-borate-EDTA buffer. The gels were stained using GelRed dye (Biotium, Hayward, California) according to the manufacturer’s protocol, and photographed in 254 nm UV light using a ChemiDoc XRS+ (Bio-Rad) system. The bands were analysed using OriginPro 8.6 software (OriginLab, Northampton, Massachusetts) and the inhibitory activity of the drugs was calculated for each one as the supercoiled to unwinded bands ratio, and normalised to the activity of camptothecin (CPT) (Sigma-Aldrich, St. Louis, Missouri).

#### Cell culture and in vitro anti-proliferation assay

Three human cell lines were used; HeLa (cervical carcinoma), A549 (lung adenocarcinoma) and SW480 (colon adenocarcinoma) were obtained from the American Type Culture Collection (ATCC, Manassas, USA). All cell lines were seeded in 96-well plates at a density of 19000 (HeLa) and 5000 (A549 and SW480) cells/well in 200 µl of Dulbeccós modified Eaglés medium (DMEM, Life Technologies, Carlsbad, USA) supplemented with 10% fetal bovine serum (FBS) (Life Technologies). Cell cultures were maintained at 37°C under 5% CO_2_.

After 5 h, cells were treated with compounds **3a**–**g**: 1 µL DMSO containing a compound to yield final concentrations of 25.0, 12.5, 6.3 and 3.1 µM per well, the final concentration of DMSO being 0.5%. Every concentration of the drugs was assayed in triplicate. In addition, every 96-well plate contained wild type cells, negative control wells (0.5% DMSO) and positive control wells containing CPT (Sigma-Aldrich) to final concentrations of 25.0 and 2.5 µM, which caused 100% cell mortality. The plate was incubated for 72 h before the fixation step.

Moreover, a no-growth control 96-well plate was seeded in every experiment. The cells on this plate were allowed to adhere for 5 h as in the experiment plate and then directly fixed, dried and stored until dying as described below, with no exposure to any compound. This no-growth plate was used to calculate the cell growth in every well of the experiment plate by subtraction of the no-growth values to the 72 h time values of the experiment plate. Experiments for every cell line were performed in triplicate.

Cells were fixed by addition of 100 µL of cold 30% (w/v) trichloroacetic acid into every well and incubated at 4°C for 1 h. Plates were then washed with water four times and dried. Protein dying was carried out with 100 µL per well of 0.057% (w/v) sulforhodamine B in 1% (v/v) acetic acid, kept at room temperature for 30 minutes. After that, excess of sulforhodamine B was removed by quickly rinsing the plates four times with 1% (v/v) acetic acid. After drying, sulforhodamine B was redissolved by adding 100 µL unbuffered Tris base solution to each well and shaking for 45 minutes on a gyratory shaker. Sulforhodamine B was quantified by its absorbance measured at 510 nm [22].

### 3. Docking Studies

#### Crystal structure preparation

The crystal structure of topotecan bound to the topoisomerase 1-DNA covalent complex was downloaded from Protein Data Bank (PDB code 1K4T) [23]. Its choice as the starting point was dictated by the fact that topotecan is the only camptothecin in clinical use that has been crystallized in its site of action. To prepare the crystal structure for docking, firstly an atom of Hg, a molecule of PEG and the two forms of topotecan (the closed lactone and the opened carboxylate) were deleted; later the Dockprep tool of the UCSF Chimera package [24] was used. This tool allows to automatically delete water molecules, add hydrogens to both the enzyme and the DNA, repair truncated side chains (Dunbrack rotamer library [25]) and assign Gasteiger charges with the AMBER ff12SB force field [26]. The input file for docking was generated with AutoDock Tools 1.5.6 [27]. The docking site was defined as a box with dimensions 15×15×20 Å, whose centroid was calculated using the coordinates of the closed lactone form of topotecan (x = 21.377, y = −4.068, z = 28.192).

#### Ligand preparation

Individual PDB files of ligands were prepared by *ab initio* energy minimization with Spartan ’10 at the 3–21G level. Hydrogens were added to all ligands and the root of torsion tree was detected. The input file for docking was generated with AutoDock Tools 1.5.6.

#### Docking studies

Docking was performed with AutoDock Vina [28] using the same parameters described above. The best pose for each ligand was selected and analyzed. To validate the docking protocol, we docked the closed lactone form of topotecan into the target binding site, after preparing both files as described above. The best resulting pose was compared with the pose of the crystal structure, and a RMSD value of 0.492 Å was calculated for the differences between the positions of the atoms in both pentacyclic scaffolds.

## Results and Discussion

### 1. Synthesis

**Figure 2:**
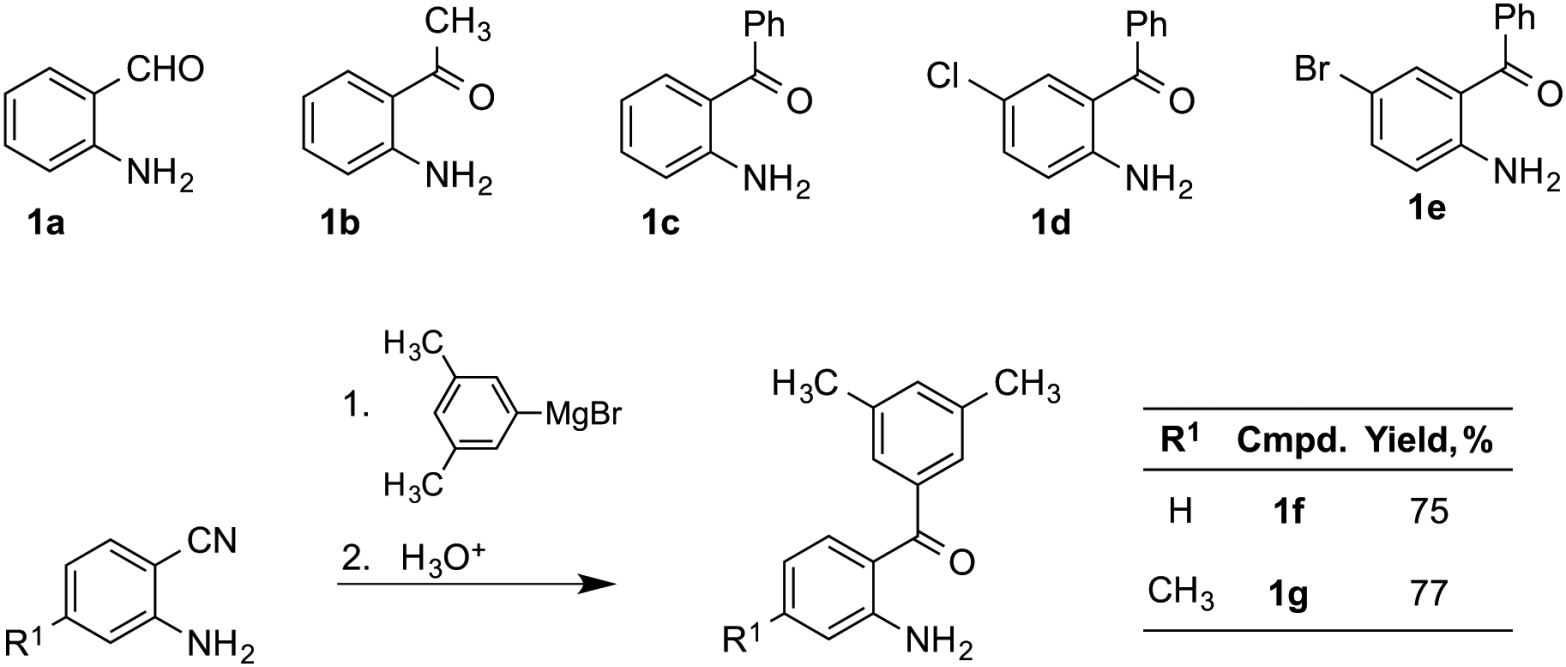
Preparation of synthetic precursors. Compounds **1a**–**d** are commercially available. Compound **1e** was prepared by bromination of **1c**, using a literature method. Compounds **1f** and **1g** were prepared by addition of 3,5-dimethylphenylmagnesium bromide to the corresponding 2-aminobenzonitrile derivative to generate an imine that was hydrolyzed *in situ* by addition of acid.

As mentioned in the Introduction, the planned Friedländer disconnection of the target compounds **3** required the preparation of two kinds of starting materials, compounds **1** and **2**. Compound **2**, which can be regarded as an oxidised derivative of the alkaloid vasicinone, was prepared using a literature method [29]. Some of the compounds **1**, namely **1a**–**d**, were commercially available, while access to **1e** was secured by a literature method involving the bromination of **1c** with potassium bromide under oxidative conditions [20]. Finally, compounds **1f** and **1g** were prepared by treatment of the corresponding derivatives of 2-aminobenzonitrile with 3,5-dimethylphenylmagnesium bromide followed by acidic hydrolysis (Figure 2).

**Figure 3:**
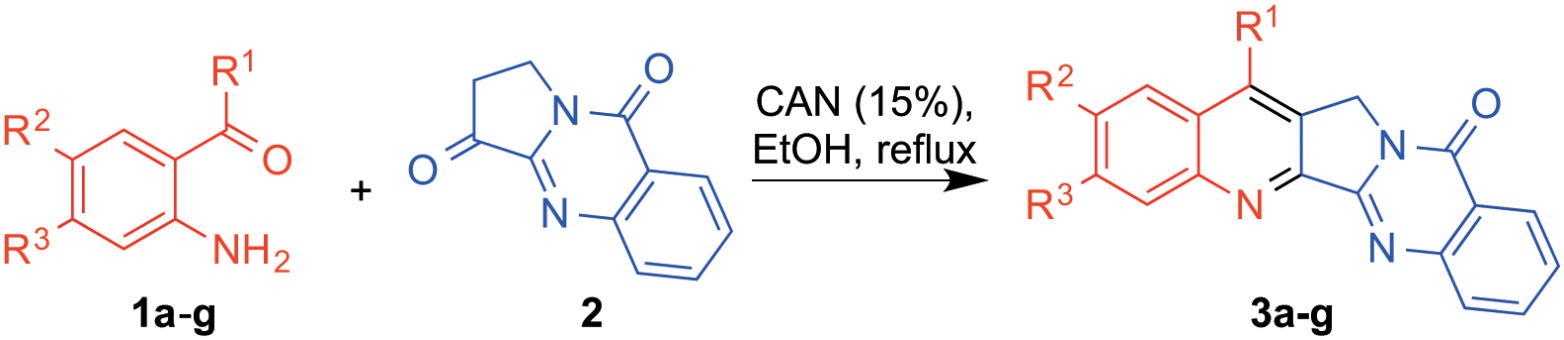
Synthesis of luotonin A and its analogues by CAN-catalyzed Friedländer reactions. The reaction between the *o*-aminocarbonyl compounds **1** and the dehydrovasicinone **2** was performed in refluxing ethanol, in the presence of Ce(IV) ammonium nitrate (CAN) as a Lewis acid.

With compounds **1** and **2** in hand, we studied their Friedländer coupling under conditions previously established by our group [19] that involved heating of the starting materials in refluxing ethanol in the presence of cerium(IV) ammonium nitrate (CAN) as a Lewis acid catalyst [30] (Figure 3). As shown in Table 1, the reaction proceeded in good to excellent yields and furnished a small library of luotonin analogues with a variety of substituents at the A and B rings. Compound **3b** had been previously prepared by a related method, albeit as a side product in a poor 14% yield, and was not tested as a topoisomerase 1 inhibitor [31].

**Table 1:** Scope and yields of the CAN-promoted Friedländer synthesis of luotonins.

R^1^	R^2^	R^3^	Cmpd.	Time, h	Yield, %
H	H	H	**3a**	1.5	66^a^
CH_3_	H	H	**3b**	24	68
Ph	H	H	**3c**	8	80
Ph	Cl	H	**3d**	8	71
Ph	Br	H	**3e**	8	64
3,5-(CH_3_)_2_C_6_H_3_	H	H	**3f**	8	90
3,5-(CH_3_)_2_C_6_H_3_	H	CH_3_	**3g**	24	85

a This reaction was described in reference 18, and is included here for comparison purposes.

**Figure 4:**
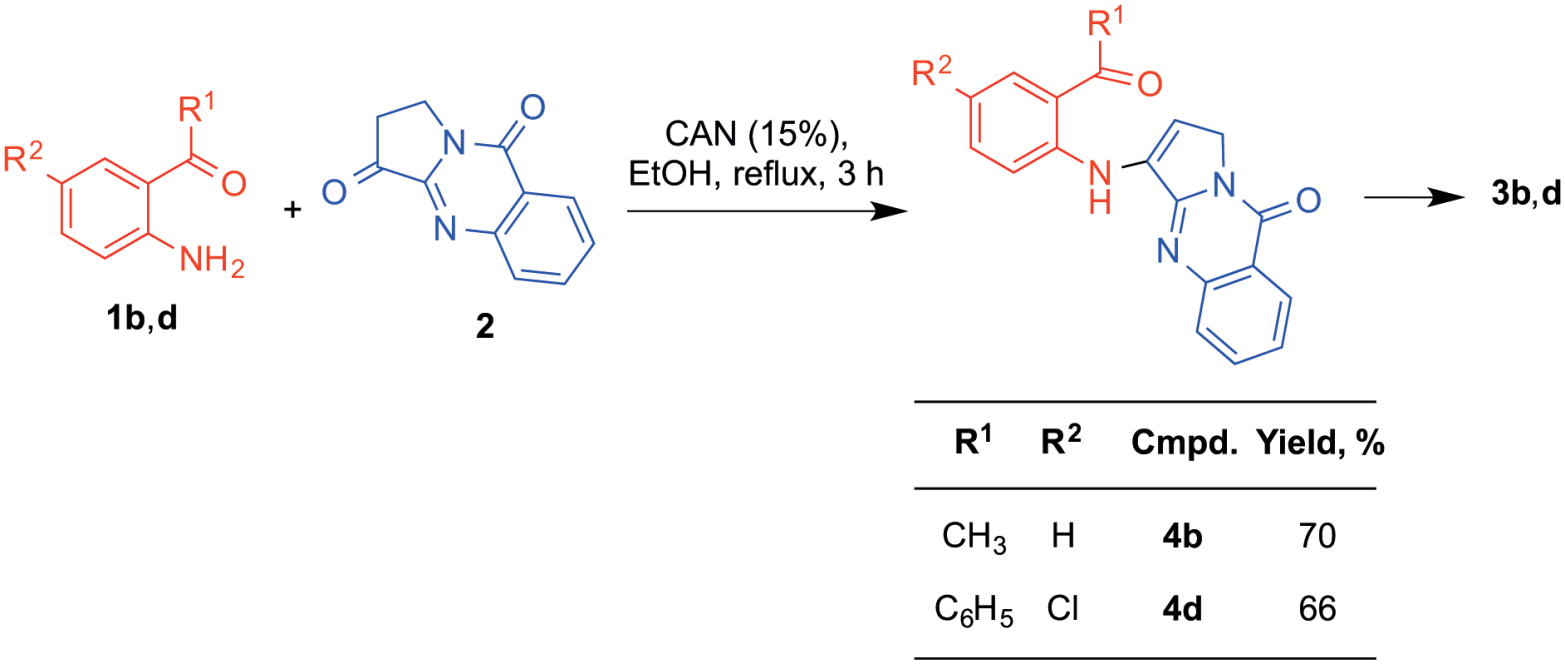
Isolation of open luotonin analogues that behave as intermediates of the Friedländer reaction. By interrupting the reaction at mid-course, it was possible to isolate enamines **4** and prove their role as intermediates of the Friedländer reaction by verifying that they afforded the final products under our standard conditions.

During the initial optimization studies, we carried out two of the reactions for shorter reaction times (3 h) and obtained the open luotonin analogues **4**, finding subsequently that they were transformed into the corresponding final products **3** by use of longer reaction times. This result proves that compounds **4** are intermediates of the CAN-catalyzed Friedländer reaction, a conclusion that is of some mechanistic interest because there is not a general agreement on whether the Friedländer reaction is initiated by an aldol reaction or by the formation of a µ-enaminone like compounds **4** (Figure 4) [32].

### 2. Pharmacological Studies

#### DNA relaxation assay

**Figure 5:**
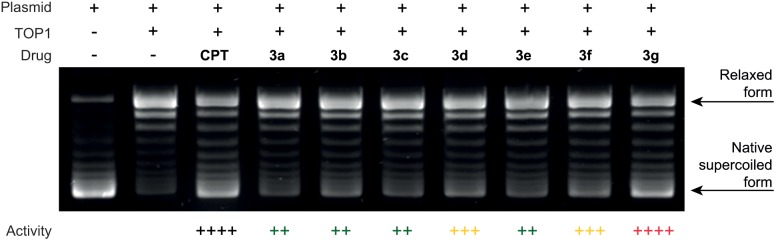
DNA relaxation inhibition assay. Drug concentration was 10 µM. Activities are expressed relative to that of camptothecin (CPT): (+) 1–25%, (++) 26–50%, (+++) 51–75%, (++++) 76–100% of the CPT inhibition. All compounds were at least as potent as luotonin A and compound **3g** showed a remarkable activity, comparable to that of camptothecin.

A representative electrophoresis gel showing the separation of DNA topomers after incubation of the plasmid in the presence of human topoisomerase 1 and the inhibitors under assay is shown in Figure 5.

When normalised to the activity of CPT, luotonin A (**3a**) showed a lower inhibitory activity (++), as expected. Interestingly, all the newly synthesized analogues exhibited an inhibitory activity similar or better than that of the reference compound, luotonin A (**3a**). Compounds **3b**, **3c** and **3e** exhibited an activity similar to that of **3a** (++). The chlorine-containing compound **3d** and the dimethylphenyl derivative **3f** showed an inhibitory potential (+++) higher than the lead compound **3a**. Finally, the methyl dimethylphenyl derivative **3g** demonstrated an excellent activity and achieved the best poisoning capacity (++++), which was close to that of camptothecin. This level of potency is remarkable in that it has very rarely been found in the inhibition of human topoisomerase by luotonin analogues [16].

**Figure 6:**
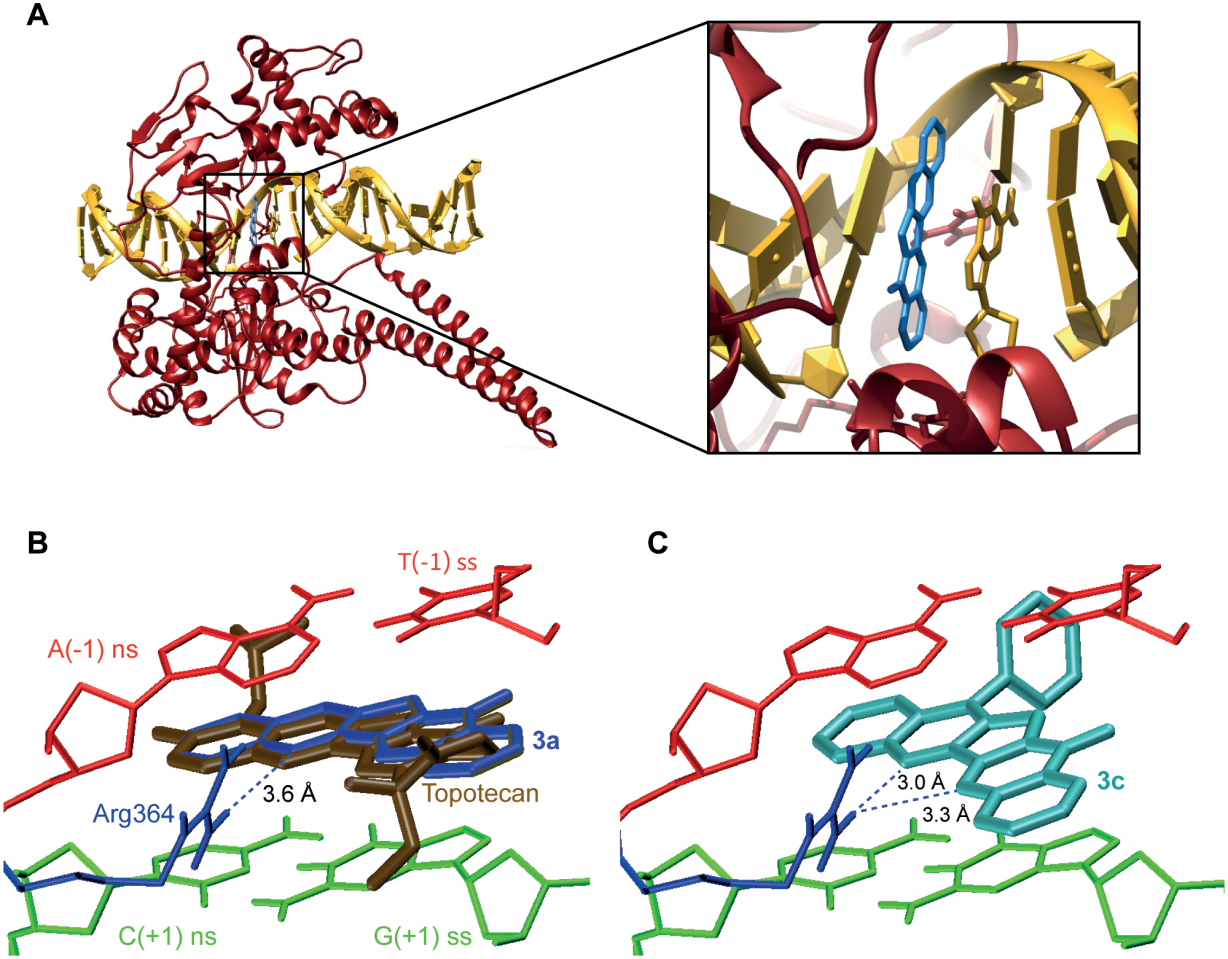
Docking of luotonin A, topotecan and compound 3c. A. A full view of the topoisomerase 1-DNA complex with luotonin A docked onto the active site. B. Expansion of the binding site. Luotonin A (navy blue) is shown compared with the position found for topotecan (brown) by crystallography, showing an excellent concordance between both poses. The base pairs are coloured in red (−1) and green (+1). The topoisomerase Arg364 residue responsible for hydrogen bonding with the compounds is displayed in blue. C. Docking of compound **3c**, showing the hydrogen bonding interaction of Arg364 with both nitrogens N5 and N6.

In order to rationalize the high activity as topoisomerase poisons of some of our luotonin analogues, and also with a view of using the information thus obtained for planning improved analogues in the future, we undertook *in silico* studies. There is no crystal structure available for the luotonin-DNA-topoisomerase 1 ternary complex and, furthermore, to the best of our knowledge, this is the first time that docking of luotonin A derivatives onto the DNA-topoisomerase 1 complex has been carried out. For this reason, we started this part of our work by studying the behaviour of luotonin A itself (**3a**), the lead compound in this series. As shown in Figures 6A and 6B, it stacked between the base pairs (−1) and (+1) and also gave hydrogen bonding interactions between N5 and Arg364. A comparison between the docking pose of luotonin with the crystal structure of the complex formed by topotecan showed that the orientation of both molecules was the same. The docking of compound **3c** is also shown (Figure 6C), showing a similar docking pose but a stronger interaction due to hydrogen bonding of its two nitrogen atoms, N5 and N6. This is a feature common to all our analogues, as will be discussed below.

The affinity of the examined molecules was quantified as the free-energy difference between the binary DNA-top1 complex and the ternary complexes formed upon binding of the drugs (“scores” in Table 2). Importantly, a good correlation was found between the ability of the molecules to stabilize the binary complex and their *in vitro* topoisomerase 1 inhibitory effects, as shown in Table 2. It is interesting to note that the two compounds bearing 3,5-dimethylphenyl substituents (**3f** and **3g**), which were the most potent of the series, were also the ones showing the highest scores.

**Table 2:** Quantification of the affinities of compounds **3a**–**g** for their binding sites and its correlation with their inhibitory activity.

Cmpd.	R^1^	R^2^	R^3^	Score (kcal/mol)	Inhibitory activity
**3a**	H	H	H	−11.4	++
**3b**	CH_3_	H	H	−11.7	++
**3c**	C_6_H_5_	H	H	−11.8	++
**3d**	C_6_H_5_	Cl	H	−12.0	+++
**3e**	C_6_H_5_	Br	H	−11.7	++
**3f**	3,5-Me_2_C_6_H_3_	H	H	−13.3	+++
**3g**	3,5-Me_2_C_6_H_3_	H	CH_3_	−13.7	++++
Camptothecin	-	-	-	−12.4	++++
Topotecan	-	-	-	−12.1	−

**Figure 7:**
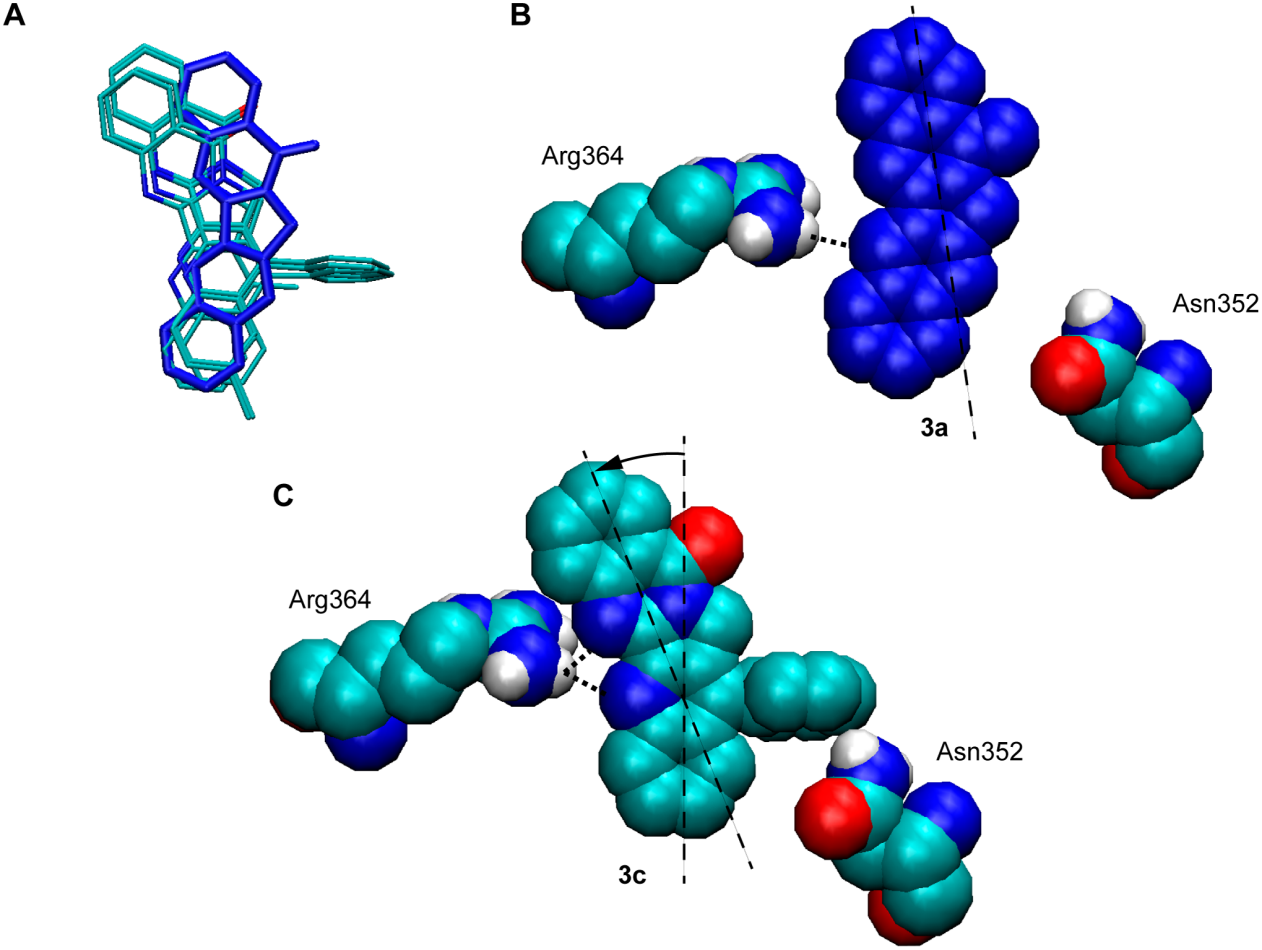
B-ring substitution improves hydrogen bonding to Arg364. **A.** Overlay of luotonin A (**3a**) and compounds **3b**–**e** at their binding sites, showing that the presence of the substituent at the C-14 position of ring B induces a rotation of the docking pose that leads to improved hydrogen bonding to Arg364 and hence to a better fit to the binding site. **B.** Docking of luotonin A showing the position of Asn352, which would interfere with any ring-B substituent. **C.** Docking of the B-phenyl substituted compound **3c,** also showing the position of Asn352.

Our docking studies revealed that the binding modes of the molecules under study could be grouped into two different types. Derivatives **3a**–**3e** (Figure 7A) bound the target site in the same fashion as the original crystallographic topotecan. All these compounds stacked between the (−1) and (+1) base pairs, perpendicular to the DNA main axis and with the two nitrogens at positions 5 and 6 facing the minor groove of the DNA. The aromatic rings in the pentacyclic structure established π-π stacking interactions, within a 3.6 Å range, with the four DNA bases flanking the molecule. A single direct protein-drug interaction was detected through hydrogen bonding between the Arg364 residue and the two basic nitrogen atoms in the molecule. Only in the case of halogenated compounds **3d** and **3e** a third class of interaction was found, as halogen bonding was possible between the chlorine or bromine atoms and the Glu356 side chain. This type of bonding is a subset of the so-called µ-hole bonds [33] and can play a significant role in molecular recognition in biological environments, as often evidenced by crystallographic studies of ligand-binding site interactions [34]. All compounds in this group pointed their E rings towards the scissile strand, although in the case of compound **3b**, another pose was found with a similar score (−11.7 *vs*. −11.6 kcal/mol) that stacks the opposite way, with the ring A on the scissile strand side.

Interestingly, compounds **3b**–**3e** showed in all cases a better affinity (“score”) than luotonin itself in our computational studies. Upon examination of the 3D docking models, this was attributed to the fact that luotonin binds to the Arg364 residue by a single hydrogen bond by the nitrogen belonging to its quinoline fragment, while in the other cases the quinoline and quinazoline fragments are both within hydrogen bonding distance of Arg364. An overlay of all five compounds at their binding sites (Figure 7A) clearly shows that the docking pose of luotonin A is different from the others. This difference may be attributed to the steric effect of the R^1^ substituent in compounds **3b**–**3e**, which would give repulsive interactions with Asn352 if their binding poses were superimposable with that of luotonin A (Figures 7B and 7C).

**Figure 8:**
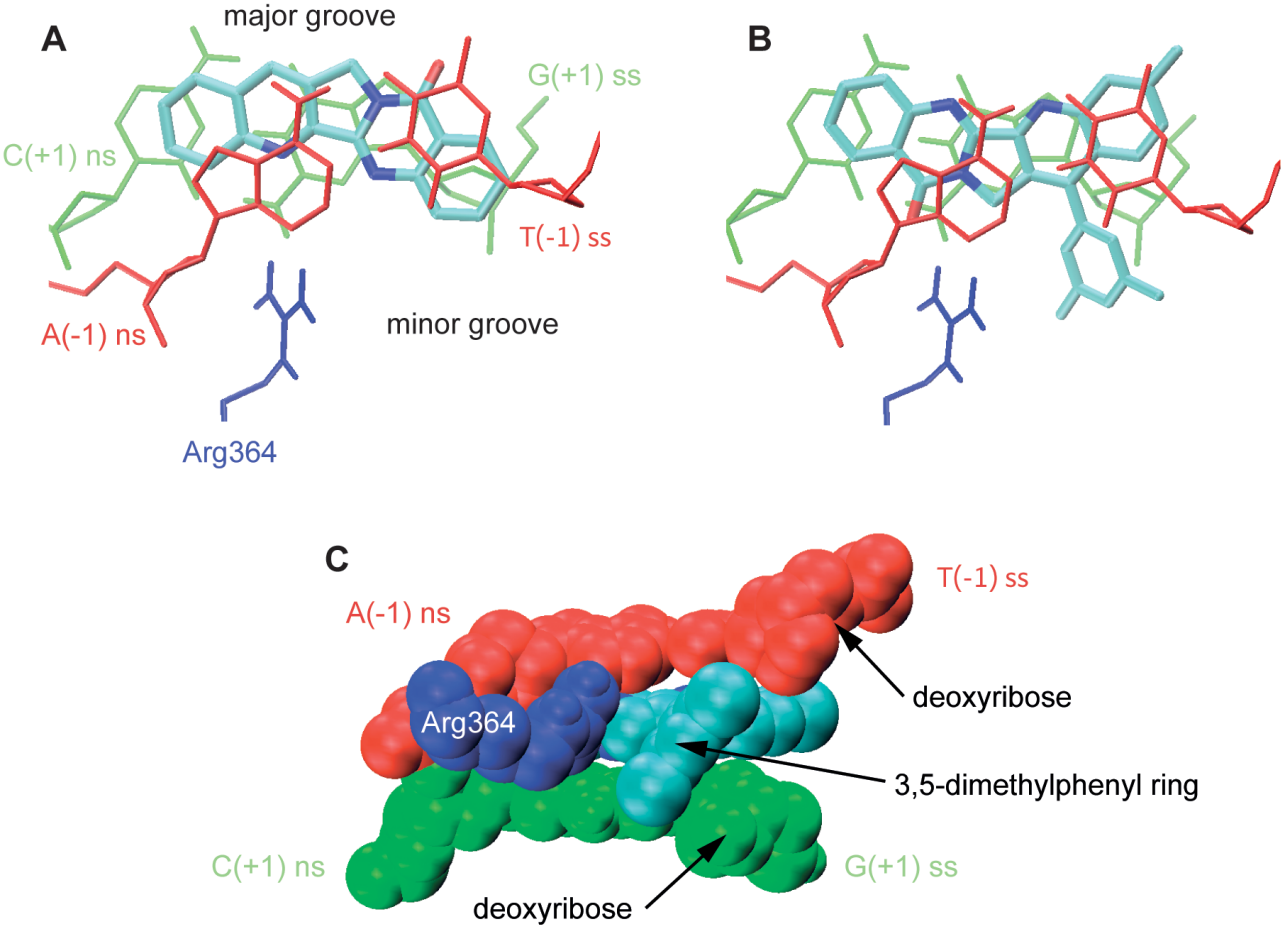
The two binding orientations of compounds 3. Docking studies revealed two different modes of binding: **A**. In the case of compounds **3a**–**e**, the docking pose was similar to the one found for topotecan by X-Ray crystallography. **B**. Compounds **3f** and **3g**, on the other hand, changed their mode of binding in order to shield the highly hydrophobic dimethylphenyl moiety from the aqueous environment by burying it between the base pairs of the scissile strand and the surface of the protein. **C** Detail of the newly described binding feature of the human topoisomerase 1-DNA complex uncovered by our study.

Compounds **3f** and **3g** showed a different binding mode (Figure 8B). Both molecules stacked between the (−1) and (+1) base pairs, but the orientation of the rings was modified. Both molecules bear a 3,5-dimethylphenyl substituent at the C-14 position of ring B that confers them a high lipophilicity [35]. Although the topotecan-like binding mode previously described allows the accommodation of large groups on position 14 of the luotonin B ring [36], nevertheless such substituents remain exposed to the highly polar environment. In this regards, it is worth noting that solvent accessible surface area for compounds **3f** (530.45 Å^2^) and **3g** (562.76 Å^2^) is at least 10% higher than the values calculated for the other luotonin derivatives [37]. The binding mode of **3f** and **3g** almost totally shields the hydrophobic dimethylphenyl moiety from the aqueous environment by burying it between the base pairs of the scissile strand and the surface of the protein, more specifically between Arg364 and the deoxyriboses belonging to G(+1) and T(−1). This minimizes the entropic effect of an ordered layer of water molecules covering the dimethylphenyl moiety [38] and enhances the stability of the ternary complex (Figure 8C). As a consequence of this binding mode, the hydrogen bonds between the basic nitrogens and Arg364 are lost. Nevertheless, this is compensated by the formation of a new, shorter hydrogen bond between the D-ring carbonyl and the basic amino acid. The dimethylphenyl group ceases to be orthogonal to the pentacyclic system in order to achieve a better fit with this binding pocket.

The main ligand-binding site interactions found in our docking studies are summarized in Table 3.

**Table 3:** Main interactions located for compounds **3a**–**g** at their binding sites.

Cmpd.	H-bonding interactions	Stacking interactions
**3a**	N5/Arg364 (3.6 Å)	Ring D,E/G(+1) (3.6 Å)
**3b**	N5/Arg364 (2.5 Å)	Ring D,E/A(−1) (3.4 Å)
	N6/Arg364 (2.7 Å)	Ring D,E/G(+1) (3.5 Å)
**3c**	N5/Arg364 (3.0 Å)	Ring A,B/G(+1) (3.6 Å)
	N6/Arg364 (3.3 Å)	
**3d** a	N5/Arg364 (3.0 Å)	Ring A,B/A(−1) (3.6 Å)
	N6/Arg364 (3.4 Å)	Ring A,B/G(+1) (3.5 Å)
**3e** b	N5/Arg364 (3.1 Å)	Ring A,B/A(−1) (3.6 Å)
	N6/Arg364 (3.5 Å)	Ring A,B/G(+1) (3.5 Å)
**3f**	Carbonyl/Arg364 (1.9 Å)	Ring A,B/T(−1) (3.6 Å)
		Ring A,B/G(+1) (3.4 Å)
		Ring D,E/A(−1) (3.5 Å)
		Ring D,E/C(+1) (3.6 Å)
**3g**	Carbonyl/Arg 364 (1.9 Å)	Ring A,B/T(−1) (3.6 Å)
		Ring A,B/G(+1) (3.4 Å)
		Ring D,E/A(−1) (3.5 Å)
		Ring D,E/C(+1) (3.6 Å)

Halogen bonds were found in these cases:

a Cl – Glu356 (3.1 Å);

b Br – Glu356 (3.1 Å).

#### Cell culture and in vitro anti-proliferation assays

**Figure 9:**
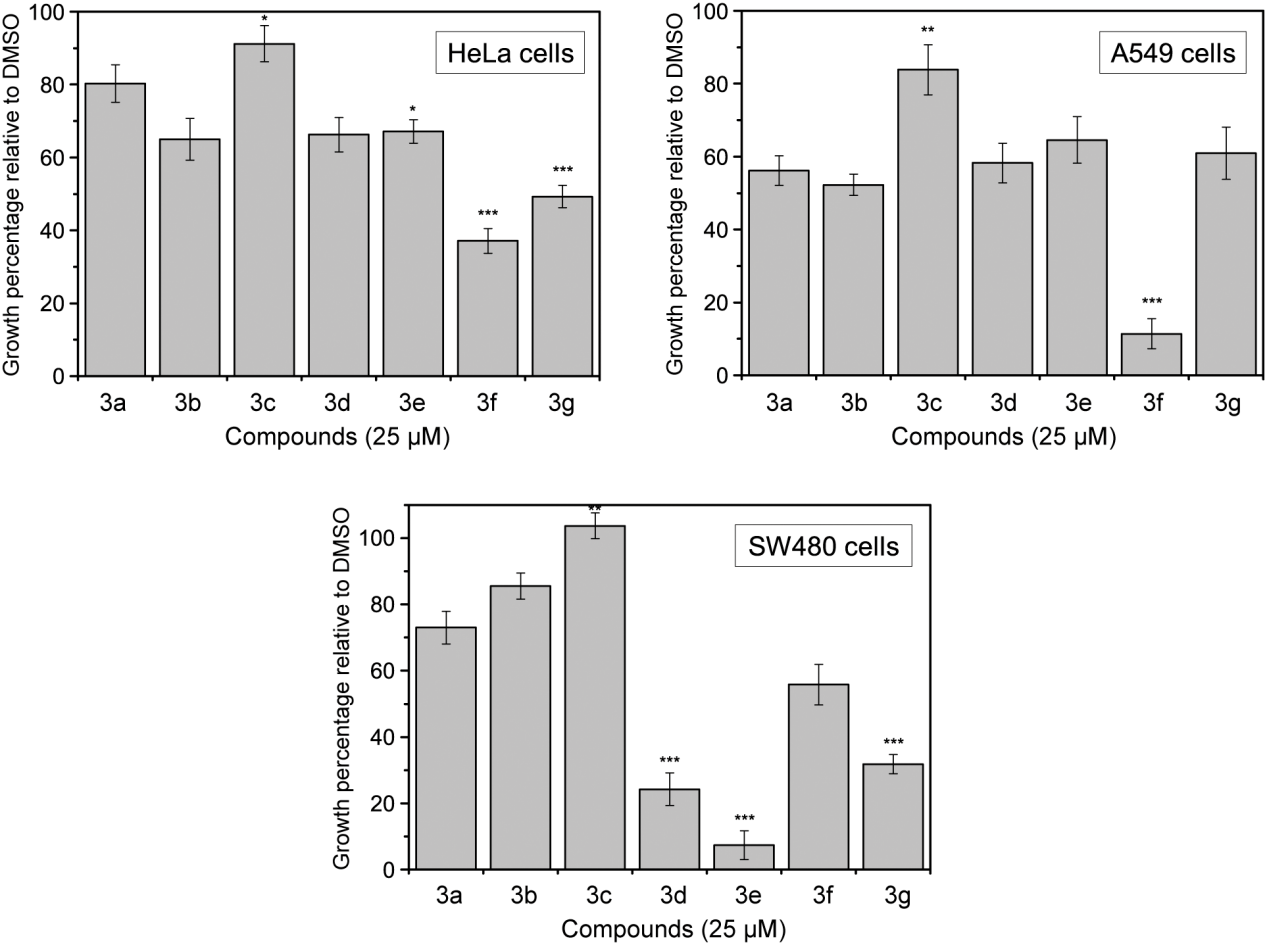
Results of the cytotoxicity assays. The cytotoxicities of compounds **3a**–**g** are expressed as percentage of cell growth relative to the negative control. Error bars account for the standard error. Asterisks indicate statistically significant differences between a drug and the model compound **3a**. Camptothecin was employed as a positive control (0% growth) at 25 and 2.5 µM concentrations.

The *in vitro* cytotoxic activity of the studied compounds was deduced from the percentage of cell growth observed when several cell cultures were exposed to 25 µM drug concentration, normalized with regard to the negative control (DMSO-treated), with the results shown in Figure 9. In the case of HeLa cells, cell growth reached an 80.3±5.1% (mean ± standard error) in the presence of the reference compound luotonin A (**3a**). The mean growth of the cells in the presence of the **3b**–**3g** derivatives was compared to that of compound **3a**, in search for statistically significant differences. All the derivatives except **3c** (91.2±4.9%, p = 0.038) showed a higher cytotoxicity than luotonin A, being particularly remarkable the results of compounds **3f** (37.1±3.4%, p<0.001) and **3g** (49.3±3.1%, p<0.001).

A similar profile was obtained in the case of lung adenocarcinoma A549 cells. All compounds showed a higher inhibitory effect in this culture than in HeLa cells, including luotonin A (56.2±4.0%). Compound **3c** was again the one with the lowest inhibitory activity (83.8±6.8%, p = 0.004). On the other hand, compound **3f** continued to be the most cytotoxic molecule, leading to a cell growth as low as 11.4±4.1% (p<0.001).

Interestingly, the tested compounds showed a different inhibition pattern in the case of SW480 (colon adenocarcinoma) cells. In this cell line, the largest inhibitory effect was exerted by compounds **3d** (24.2±4.9%), **3e** (7.3±4.3%) and **3g** (31.8±2.9%) all of them with a p value<0.001 with respect to luotonin A (73.02±4.9%). Taken in the aggregate, these results unveil a selectivity inhibitory activity pattern of the halogenated derivatives **3d** and **3e** against the colon adenocarcinoma cell line and of compound **3f** against the lung adenocarcinoma cell line. This is an interesting feature, since a high potency against all cell lines is usually regarded as a proof of indiscriminate toxicity and therefore as an undesirable property.

The fact that the topoisomerase inhibition data showed a less than perfect correlation with the cytotoxicity results can be attributed to the participation in the latter of physicochemical characteristics and pharmacokinetic phenomena such as solubility and transport across membranes.

When examined together with the results from the topoisomerase 1 inhibition assay, the outcome from the cell cytotoxicity experiments supports the conclusion that compounds **3f** and **3g** are the most promising therapeutic candidates, showing a much higher potency than the reference luotonin A in the three cell lines assayed. Also compounds **3d** and **3e**, which showed a moderate activity in the topoisomerase 1 inhibition experiments, have been revealed as interesting alternatives, with good results in the colon adenocarcinoma cell line. The high selectivity of these chloro- and bromo- derivatives towards SW480 cells is remarkable and shall be the subject of further research.

## Conclusions

The CAN-catalyzed Friedländer reaction provides a reliable and efficient method for the synthesis of luotonin A analogues modified at their A and B rings. All analogues showed an activity similar or higher in topoisomerase 1 inhibition in comparison to the reference compound luotonin A, and some compounds even had an activity comparable to camptothecin, which is an unusually good result for series of analogues of this alkaloid. Furthermore, almost all compounds showed a better activity than luotonin A in cell cytotoxicity assays. In order to rationalize these results, docking studies of luotonin A-topoisomerase 1-DNA ternary complexes were undertaken for the first time, and led to the conclusion that most compounds bound in a manner similar to standard topoisomerase poisons such as topotecan and also to luotonin A, although the presence of the substituent at the C-14 position of ring B induced in most cases a better fit to the binding site due to improved hydrogen bonding to Arg364. Interestingly, the two most promising compounds, bearing a 3,5-dimethylphenyl substituent at ring B, docked in a different orientation, with this hydrophobic moiety shielded from the aqueous environment by being buried between the deoxyribose belonging to the G(+1) nucleotide in the scissile strand and the Arg364 on the surface of the protein, additionally forming a hydrogen bond between the D-ring carbonyl and the basic amino acid. The discovery of this new binding mode and its associated higher inhibitory potency is a significant advance that will find application in the design of new topoisomerase 1 inhibitors.

## Supporting Information

File S1 SI1, ^1^H and ^13^C^–^NMR spectra of all compounds. Figure S1, Two views of compound 3a (luotonin A) docked in the topoisomerase 1 active site. Figure S2, Two views of compound 3b docked in the topoisomerase 1 active site. Figure S3, Two views of compound 3c docked in the topoisomerase 1 active site. Figure S4, Two views of compound 3d docked in the topoisomerase 1 active site. Figure S5, Two views of compound 3e docked in the topoisomerase 1 active site. Figure S6, Two views of compound 3f docked in the topoisomerase 1 active site. Figure S7, Two views of compound 3g docked in the topoisomerase 1 active site.
(ZIP)

## References

[1] Ferlay J, Soerjomataram EM, Dikshit R, Eser S, Mathers C, et al. (2013) GLOBOCAN 2012 v1.0, Cancer incidence and mortality worldwide: IARC CancerBase No. 11 [Internet]. Lyon, France: International Agency for Research on Cancer. Available: http://globocan.iarc.fr, accessed on 22/01/2014.

[2] Levin B, Boyle P (Eds.) (2008) World cancer report. Lyon: International Agency for Research on Cancer, WHO Press.

[3] Avendaño C, Menéndez JC (2008) Medicinal chemistry of anticancer drugs. Amsterdam: Elsevier.

[4] Chen SH, Chan NL, Hsieh TS (2013) New mechanistic and functional insights into DNA topoisomerases. Ann Rev Biochem 82: 139–170. 23495937 10.1146/annurev-biochem-061809-100002

[5] Pommier Y (2011) DNA topoisomerases and cancer. New York: Springer.

[6] Pommier Y (2013) Drugging topoisomerases: Lessons and challenges. ACS Chem Biol 8: 82–95. 23259582 10.1021/cb300648v PMC3549721

[7] Kacprzak KM (2013) Chemistry and biology of camptothecin and its derivatives, in Ramawat KG, Mérillon JM (Eds.) Natural Products (643–682) Heidelberg, Berlin: Springer.

[8] Castelli S, Coletta A, D’Anness I, Fiorani P, Tesauro C, et al (2012) Interaction between natural compounds and human topoisomerase 1. Biol Chem 393: 1327–1340. 23109546 10.1515/hsz-2012-0240

[9] Venditto VJ, Simanek EE (2010) Cancer therapies utilizing the camptothecins: a review of the *in vivo* literature. Mol Pharmaceutics 7: 307–349. 10.1021/mp900243b PMC3733266 20108971

[10] Pommier Y (2009) DNA topoisomerase 1 inhibitors: Chemistry, biology, and interfacial inhibition. Chem Rev 109: 2894–2902. 19476377 10.1021/cr900097c PMC2707511

[11] Marszałł MP, Buciński A, Kruszewski S, Ziomkowska B (2011) A new approach to determine camptothecin and its analogues affinity to human serum albumin. J Pharm Sci 100: 1142–1146. 20740669 10.1002/jps.22318

[12] Pizzolato JF, Saltz LB (2003) The camptothecins. Lancet 361: 2235–2242. 12842380 10.1016/S0140-6736(03)13780-4

[13] Cagir A, Jones SH, Gao R, Eisenhauer BMA, Hecht SM (2003) Luotonin A. A naturally occurring human DNA topoisomerase 1 poison. J Am Chem Soc 125: 13628–13629. 14599178 10.1021/ja0368857

[14] Dallavalle S, Merlini L, Beretta G, Tinelli S, Zunino F (2004) Synthesis and cytotoxic activity of substituted luotonin A derivatives. Bioorg Med Chem Lett 14: 5757–5761. 15501036 10.1016/j.bmcl.2004.09.039

[15] Liang JL, Cha HC, Jahng Y (2011) Recent advances in the studies on luotonins. Molecules 16: 4861–4883. 21677601 10.3390/molecules16064861 PMC6264391

[16] Tseng MC, Chu YW, Tsai HP, Lin CM, Hwang J, et al (2011) One-pot synthesis of luotonin A and its analogues. Org Lett 13: 920–923. 21268635 10.1021/ol1029707

[17] Boisse T, Gavara L, Gautret P, Baldeyrou B, Lansiaux A, et al (2011) Toward new camptothecins. Part 7: Synthesis of thioluotonine and its 5-methoxycarbonyl derivative. Tetrahedron Lett 52: 1592–1596.

[18] Golubev AS, Bogomolov VO, Shidlovskii AF, Dezhenkova LG, Peregudov AS, et al (2011) Synthesis of fluoromethyl-containing analogs of antitumor alkaloid luotonin A. Russ Chem Bull, Int Edition. 59: 209–218.

[19] Sridharan V, Ribelles P, Ramos MT, Menéndez JC (2009) Cerium(IV) ammonium nitrate is an excellent, general catalyst for the Friedländer and Friedländer-Borsche quinoline syntheses: Very efficient access to the antitumor alkaloid luotonin A. J Org Chem. 74: 5715–5718. 10.1021/jo900965f 19534479

[20] Atechian S, Nock N, Norcross RD, Ratni H, Thomas AW, et al (2007) New vistas in quinoline synthesis. Tetrahedron 63: 2811–2823.

[21] Ma ZZ, Hano Y, Nomura T, Chen YJ (1997) Two new pyrroloquinazolino-quinoline alkaloids from *Peganum nigellastrum* . Heterocycles 46: 541–546.

[22] Vichai V, Kirtikara K (2006) Sulforhodamine B colorimetric assay for cytotoxicity screening. Nature Protocols 1: 1112–1116. 17406391 10.1038/nprot.2006.179

[23] Staker BL, Hjerrild K, Feese MD, Behnke CA, Burgin AB, et al (2002) The mechanism of topoisomerase 1 poisoning by a camptothecin analog. Proc Natl Acad Sci USA 99: 15387–15392. 12426403 10.1073/pnas.242259599 PMC137726

[24] Pettersen EF, Goddard TD, Huang CC, Couch GS, Greenblatt DM, et al (2004) UCSF Chimera - A visualization system for exploratory research and analysis. J Comput Chem 25: 1605–1612. 15264254 10.1002/jcc.20084

[25] Dunbrack Jr RL (2002) Rotamer libraries in the 21st century. Curr Opin Struct Biol 12: 431–440. 12163064 10.1016/s0959-440x(02)00344-5

[26] Wang J, Wang W, Kollman PA, Case DA (2006) Automatic atom type and bond type perception in molecular mechanical calculations. J Mol Graph Modelling 25: 247–260. 10.1016/j.jmgm.2005.12.005 16458552

[27] Morris GM, Huey R, Lindstrom W, Sanner MF, Belew RK, et al (2009) AutoDock4 and AutoDockTools4: automated docking with selective receptor flexiblity. J Comput Chem 16: 2785–2791. 10.1002/jcc.21256 PMC2760638 19399780

[28] Trott O, Olson AJ (2010) AutoDock Vina: improving the speed and accuracy of docking with a new scoring function, efficient optimization and multithreading. J. Comput Chem 31: 455–461. 19499576 10.1002/jcc.21334 PMC3041641

[29] Molina P, Tárraga A, González-Tejero A (2000) A convenient divergent approach to the alkaloids isaindigotone and luotonin A. Synthesis 1523–1525.

[30] Sridharan V, Menéndez JC (2010) Cerium(IV) ammonium nitrate as a catalyst in organic synthesis. Chem Rev 110: 3805–3849. 20359233 10.1021/cr100004p

[31] Rahman AFMM, Kim DH, Liang JL, Lee ES, Na Y, et al (2008) Synthesis and biological properties of luotonin A derivatives. Bull Kor Chem Soc 29: 1988–1992.

[32] Marco JL, Pérez-Mayoral E, Samadi A, Carreiras MC, Soriano E (2009) Recent advances in the Friedländer reaction. Chem Rev 109: 2652–2671. 19361199 10.1021/cr800482c

[33] Politzer P, Murray JS, Clark T (2013) Halogen bonding and other σ-hole interactions: A perspective. Phys Chem Chem Phys 15: 11178–11189. 23450152 10.1039/c3cp00054k

[34] Howard EI, Sanishvili R, Cachau RE, Mitschler A, Chevrier B, et al (2004) Ultrahigh resolution drug design I: Details of interactions in human aldose reductase-inhibitor complex at 0.66 Å. Prot Struct Funct Bioin. 55: 792–804. 10.1002/prot.20015 15146478

[35] González-Ruiz V, Mussardo P, Corda E, Girotti S, Olives AI, et al (2010) Liquid chromatographic analysis of the anticancer alkaloid luotonin A and some new derivatives in human serum samples. J Sep Sci 33: 2086–2093. 20568252 10.1002/jssc.201000175

[36] Verma RP, Hansch C (2009) Camptothecins: A SAR/QSAR study. Chem Rev 109: 213–235. 19099450 10.1021/cr0780210

[37] Available: www.chemicalize.org.Accessed on 22/01/2014.

[38] Wadkins RM, Bearss D, Manikumar G, Wani MC, Wall ME, et al (2004) Topoisomerase 1-DNA complex stability induced by camptothecins and its role in drug activity. Curr Med Chem-Anti- Cancer Agents 4: 327–334. 10.2174/1568011043352894 15281905

