# Supplementary material for: B-Ring-Aryl Substituted Luotonin A Analogues with a New Binding Mode to the Topoisomerase 1-DNA Complex Show Enhanced Cytotoxic Activity
Source: PLoS One. 2014 May 15;9(5):e95998. doi: 10.1371/journal.pone.0095998 (PMC4022624; doi:10.1371/journal.pone.0095998)
Supplement: File S1 — SI1, 1H and 13C–NMR spectra of all compounds. Figure S1, Two views of compound 3a (luotonin A) docked in the topoisomerase 1 active site. Figure S2, Two views of compound 3b docked in the topoisomerase 1 active site. Figure S3, Two views of compound 3c docked in the topoisomerase 1 active site. Figure S4, Two views of compound 3d docked in the topoisomerase 1 active site. Figure S5, Two views of compound 3e docked in the topoisomerase 1 active site. Figure S6, Two views of compound 3f docked in the topoisomerase 1 active site. Figure S7, Two views of compound 3g docked in the topoisomerase 1 active site. (ZIP) [file pone.0095998.s001.zip › Supporting info/Copies of spectra.pdf]

Supporting information

B-ring-aryl substituted luotonin analogues with a new binding mode to the topoisomerase I-DNA complex show enhanced anticancer activity

Víctor González-Ruiz,[1,2] Irene Pascua,[3,4] Tamara Fernández-Marcelo,[3,4] Pascual Ribelles,[2,5] Giulia Bianchini,[2,5] Vellaisamy Sridharan,[5,6] Pilar Iniesta,[3,4]* M. Teresa Ramos,[2,5] Ana I. Olives,[1,2] M. Antonia Martín,[1,2]* J. Carlos Menéndez[2,5]*

1 Sección Departamental de Química Analítica, Facultad de Farmacia, Universidad Complutense, 28040 Madrid, Spain.

2 BIOHET (Biologically Relevant Heterocycles) group, Facultad de Farmacia, Universidad Complutense, 28040 Madrid, Spain.

3 Departamento de Bioquímica y Biología Molecular II, Facultad de Farmacia, Universidad Complutense, 28040 Madrid, Spain.

4 Instituto de Investigación Sanitaria del Hospital Clínico San Carlos. C/ Profesor Martín Lagos s/n, 28040, Madrid, Spain.

5 Departamento de Química Orgánica y Farmacéutica, Facultad de Farmacia, Universidad Complutense, 28040 Madrid, Spain.

6 Department of Chemical and Biotechnology, SASTRA University, Thanjavur, 613401, India

e-mail: josecm@farm.ucm.es

Copies of spectra of all compounds

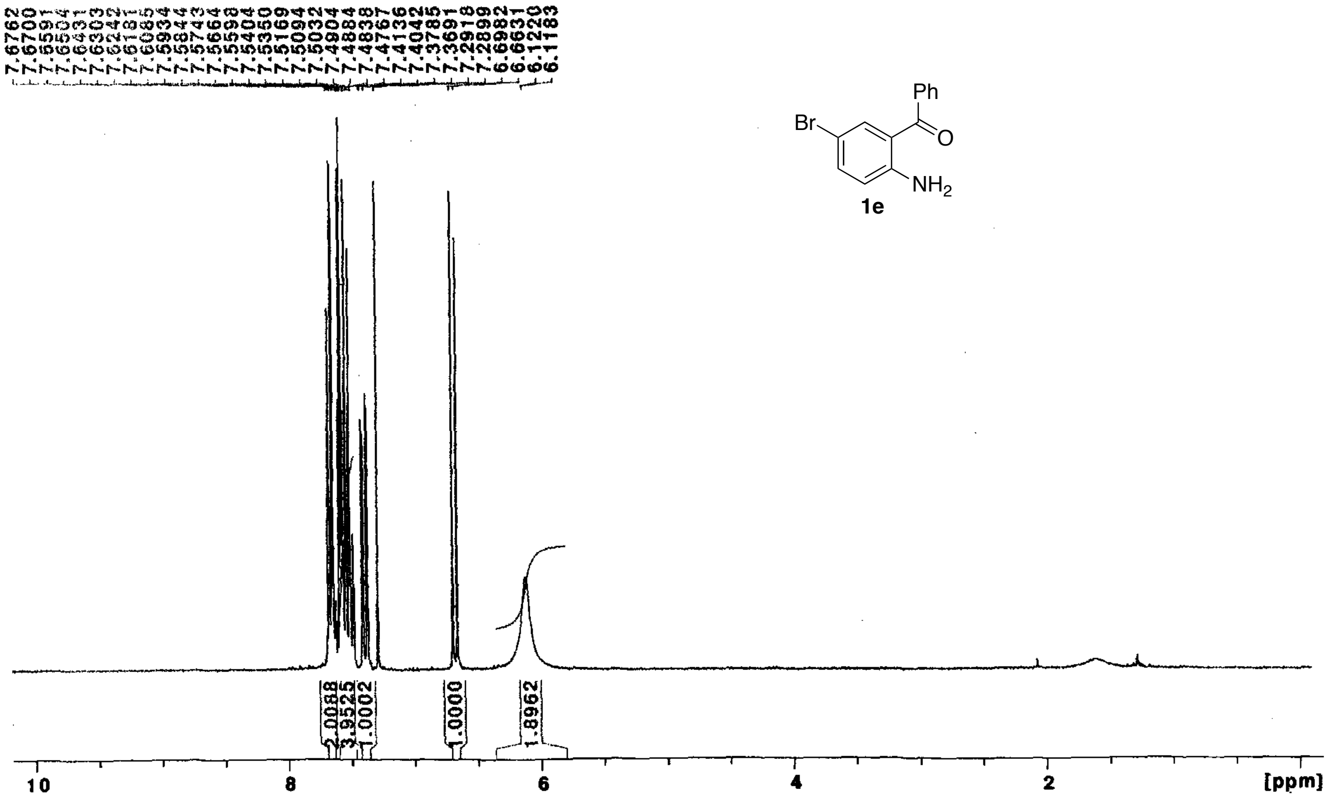

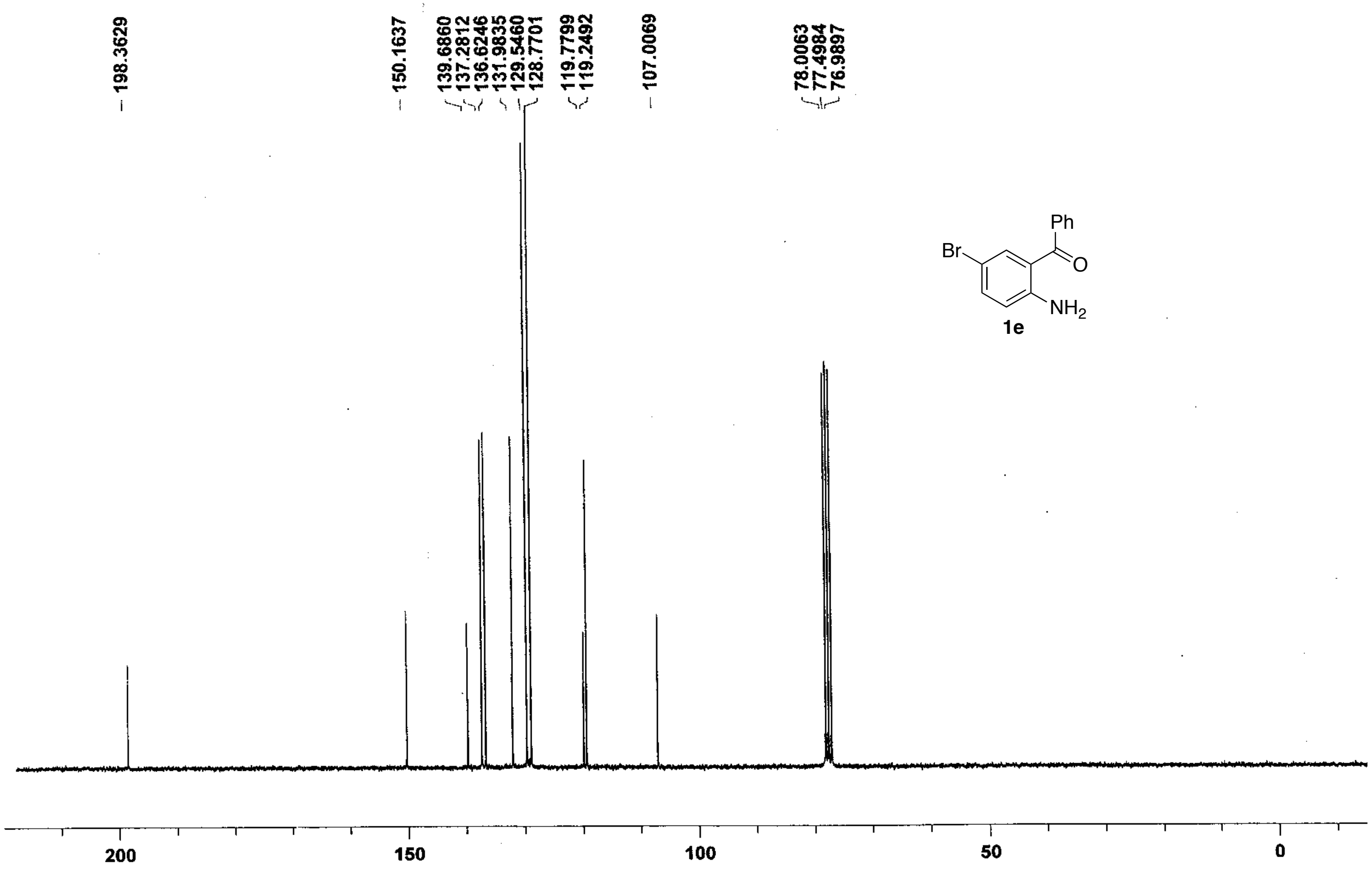
198.3629
150.1637
139.6860
137.2812
136.6246
131.9835
129.5460
128.7701
119.7799
119.2492
107.0069
78.0063
77.4984
76.9897
Ph
Br
O
NH2
1e
200
150
100
50
0

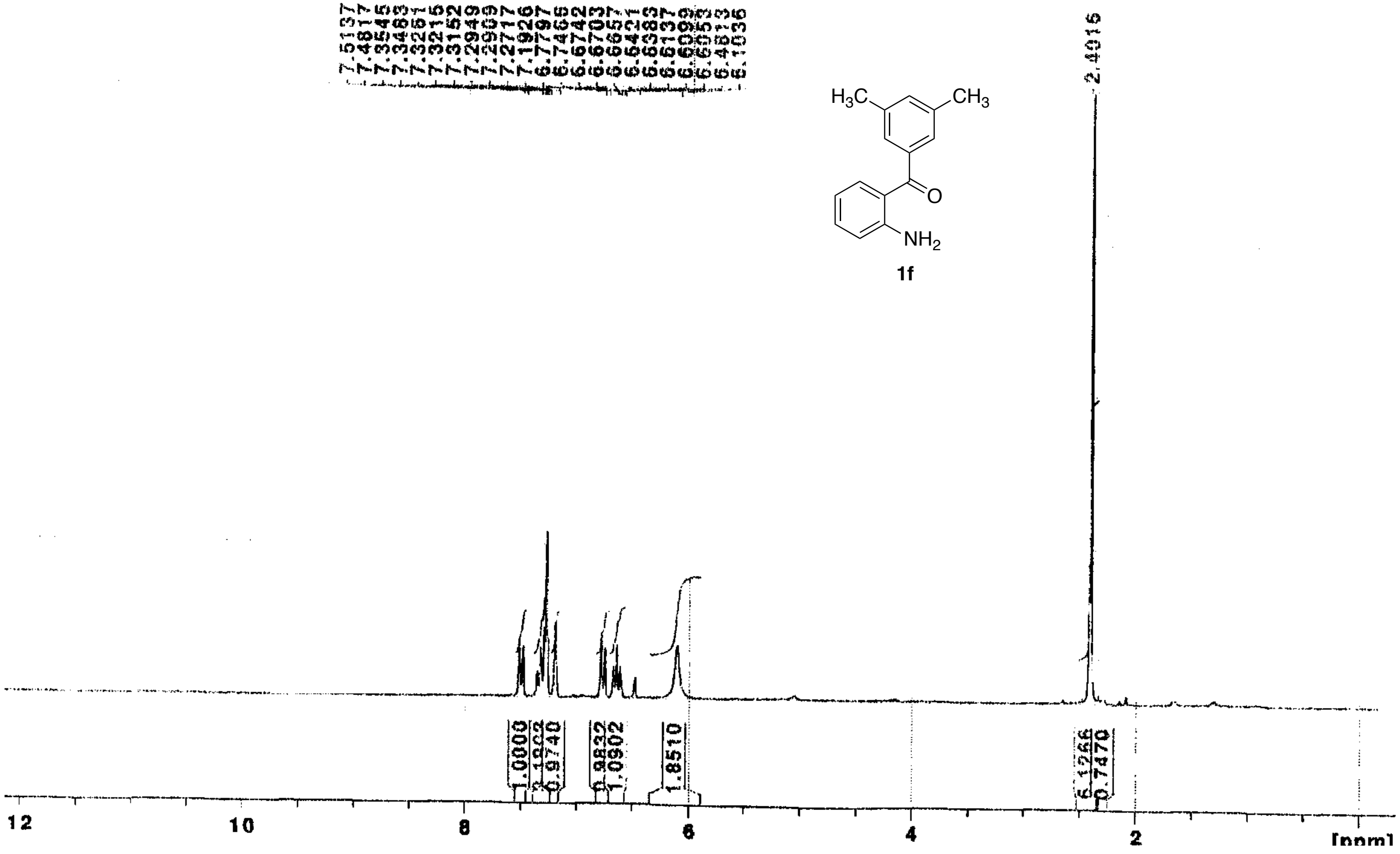

7.5137
7.4817
7.3545
7.3463
7.3261
7.3215
7.3152
7.2949
7.2909
7.2717
7.1926
6.7797
6.7466
6.6742
6.6703
6.6657
6.6421
6.6383
6.6137
6.6099
6.6053
6.4813
6.1036
2.4016
H3C
CH3
O
NH2
1f
1.0000
2.1202
0.9740
0.9832
1.0902
1.8510
6.1266
0.7470
12
10
8
6
4
2
[ppm]

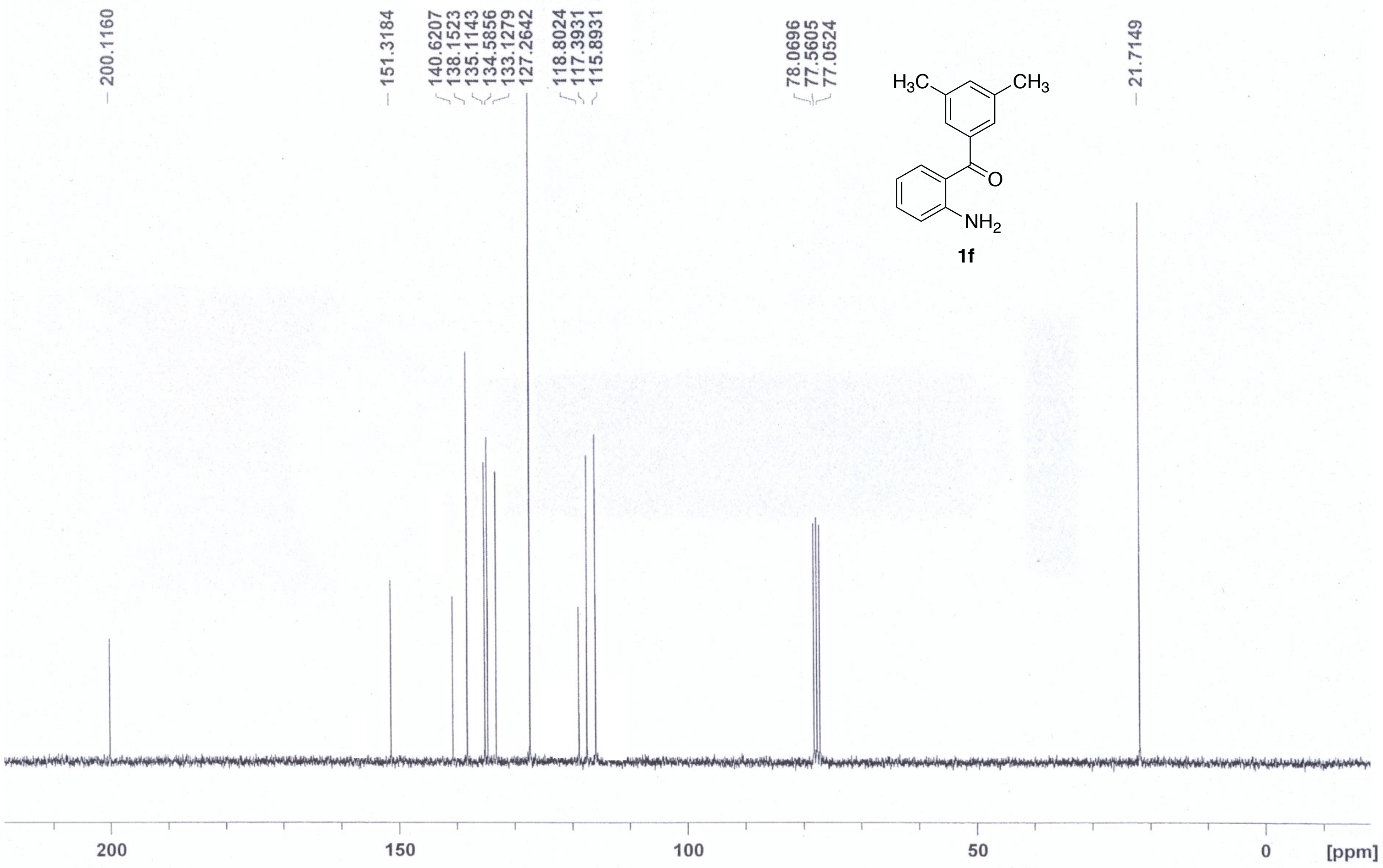
200.1160
151.3184
140.6207
138.1523
135.1143
134.5856
133.1279
127.2642
118.8024
117.3931
115.8931
78.0696
77.5605
77.0524
21.7149
200
150
100
50
0
[ppm]

1f

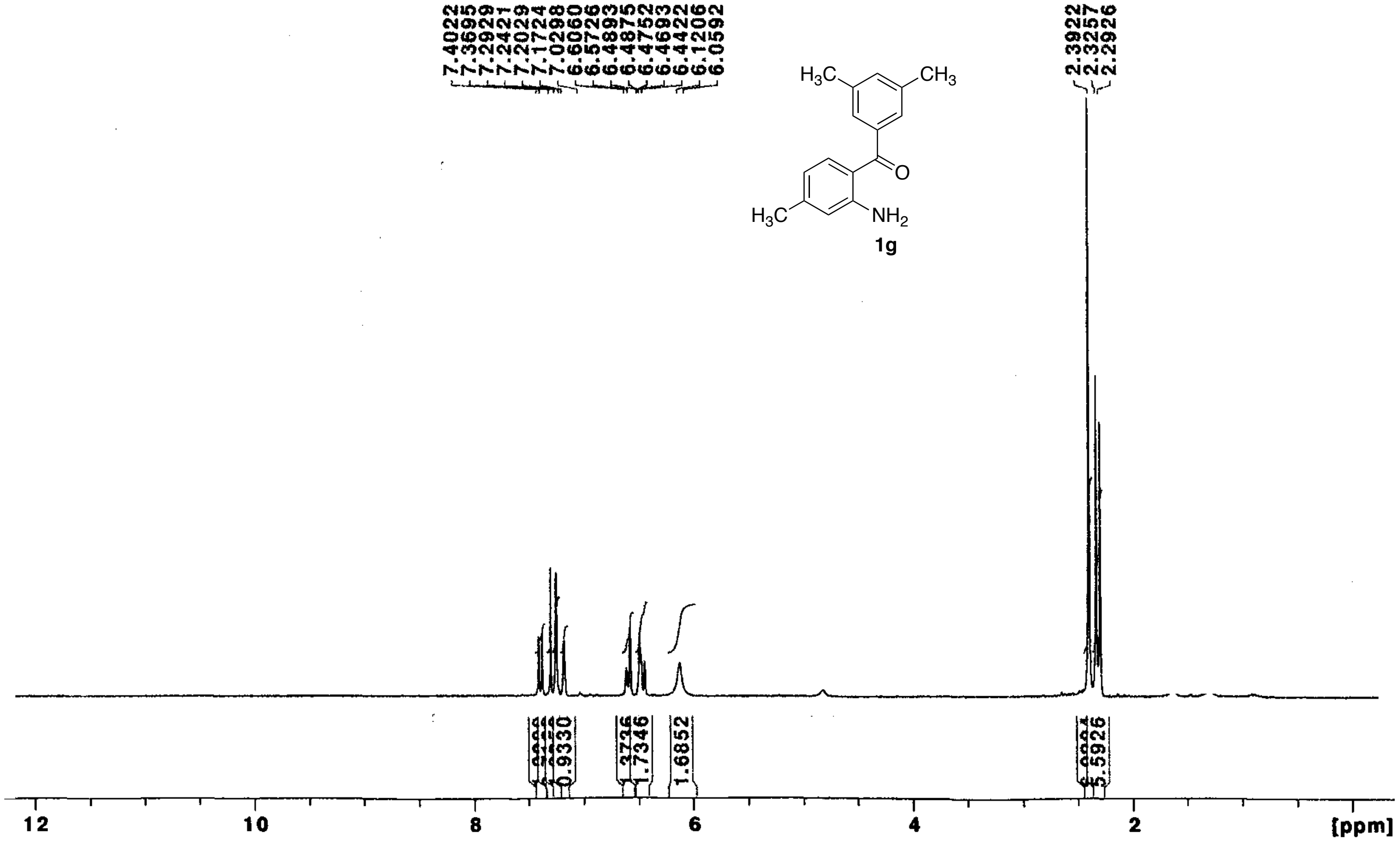

7.4022
7.3695
7.2929
7.2421
7.2029
7.1724
7.0298
6.6060
6.5726
6.4893
6.4875
6.4752
6.4693
6.4422
6.1206
6.0592
2.3922
2.3257
2.2926
H3C
CH3
O
H3C
NH2
1g
0.9330
1.7346
1.6852
5.5926
12
10
8
6
4
2
[ppm]

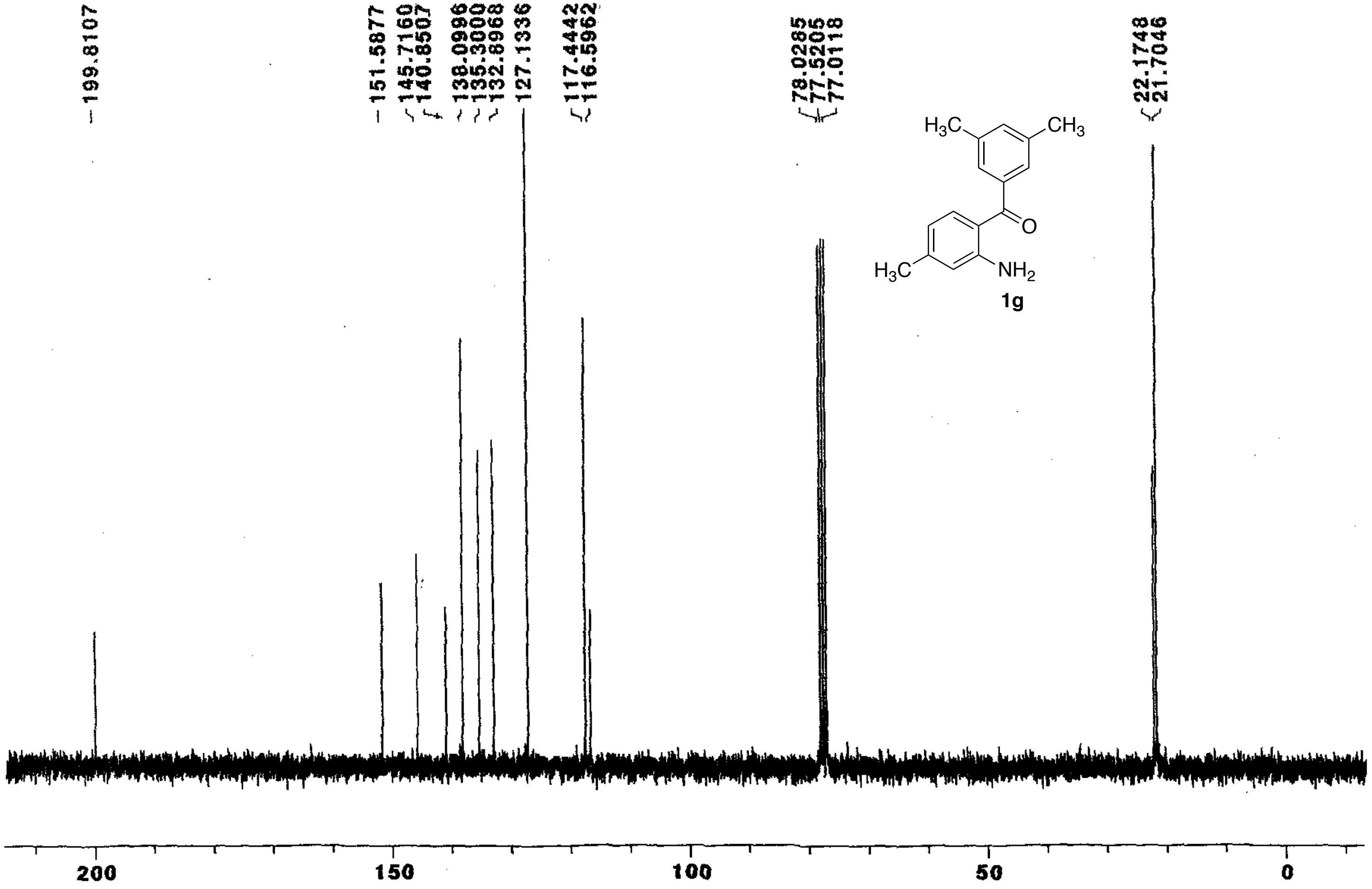

199.8107
151.5877
145.7160
140.8507
138.0996
135.3000
132.8968
127.1336
117.4442
116.5962
78.0285
77.5205
77.0118
22.1748
21.7046
H3C
CH3
O
H3C
NH2
1g
200
150
100
50
0

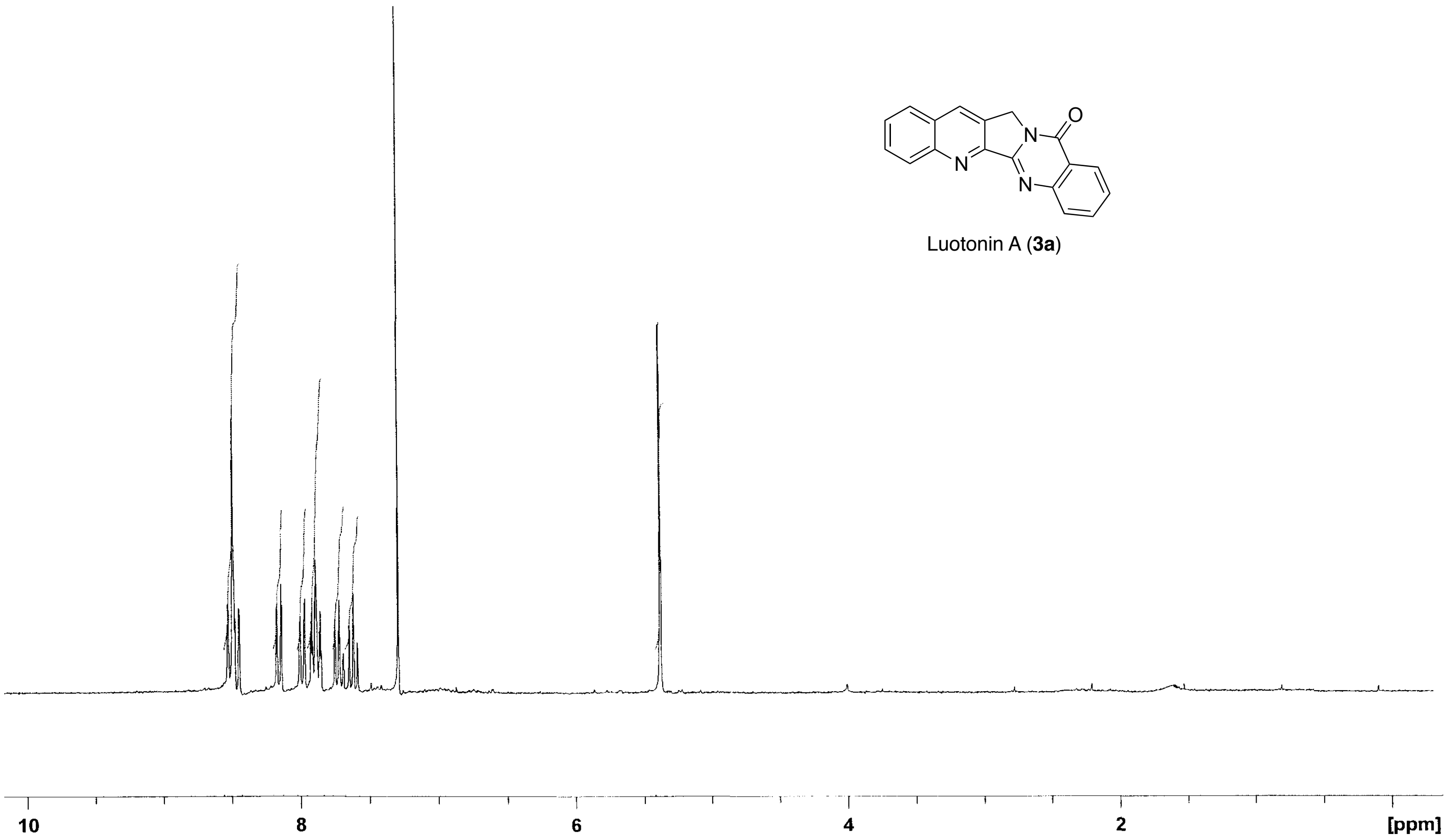

Luotonin A (**3a**)

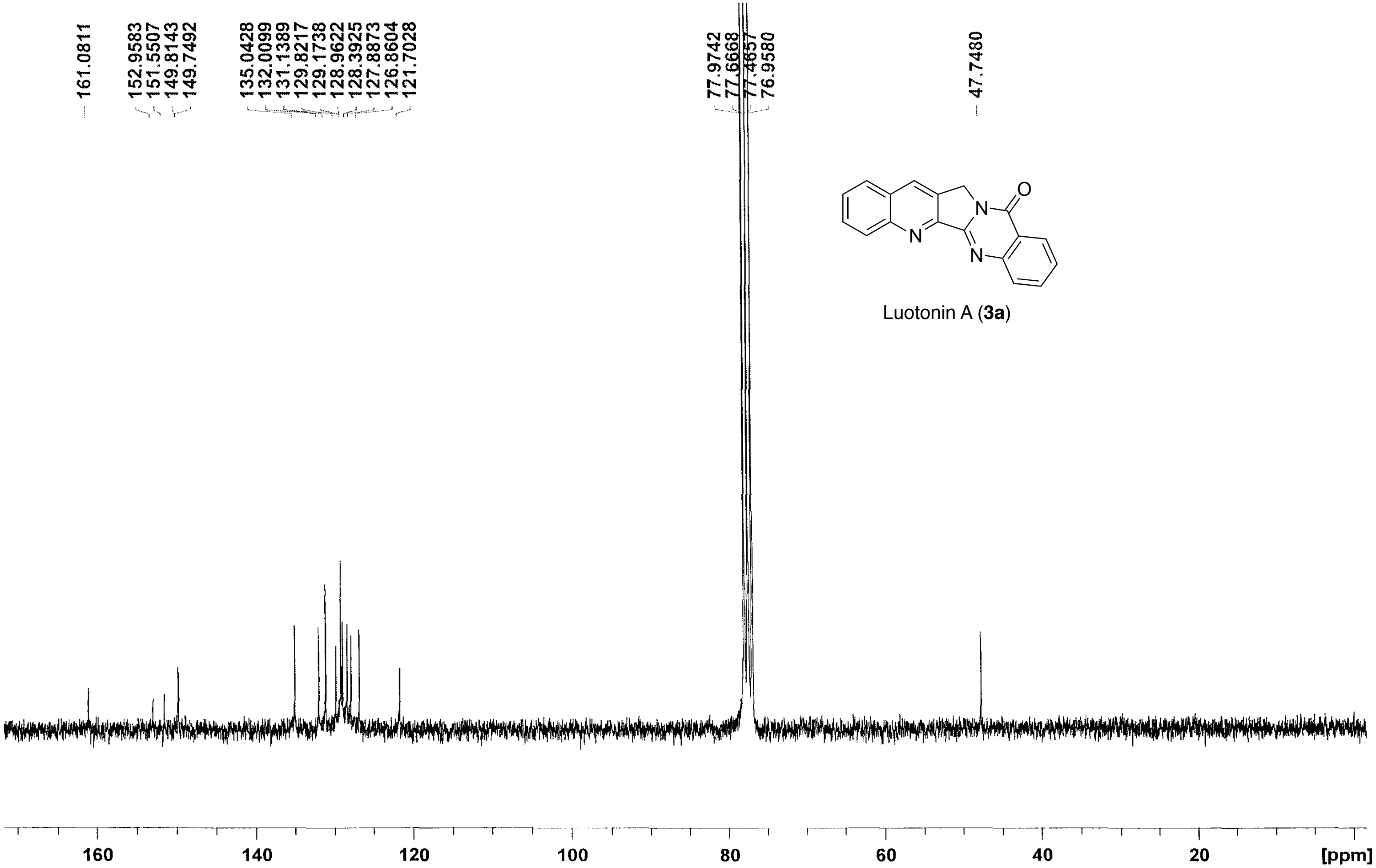
161.0811
152.9583
151.5507
149.8143
149.7492
135.0428
132.0099
131.1389
129.8217
129.1738
128.9622
128.3925
127.8873
126.8604
121.7028
77.9742
77.6668
77.4657
76.9580
47.7480
Luotonin A (**3a**)
160
140
120
100
80
60
40
20
[ppm]

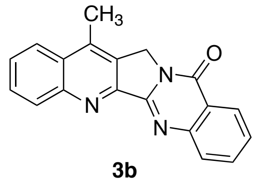

3b

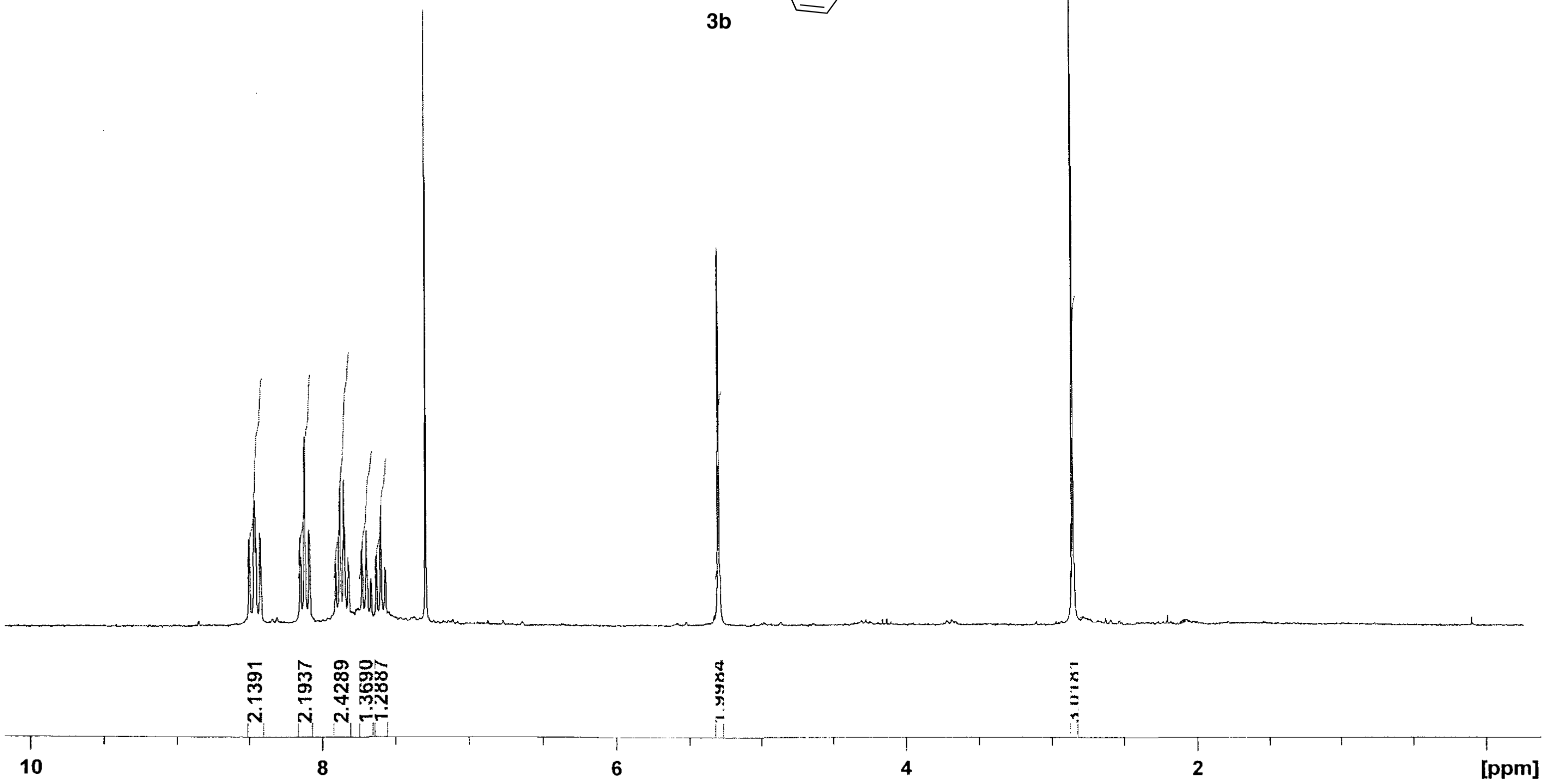

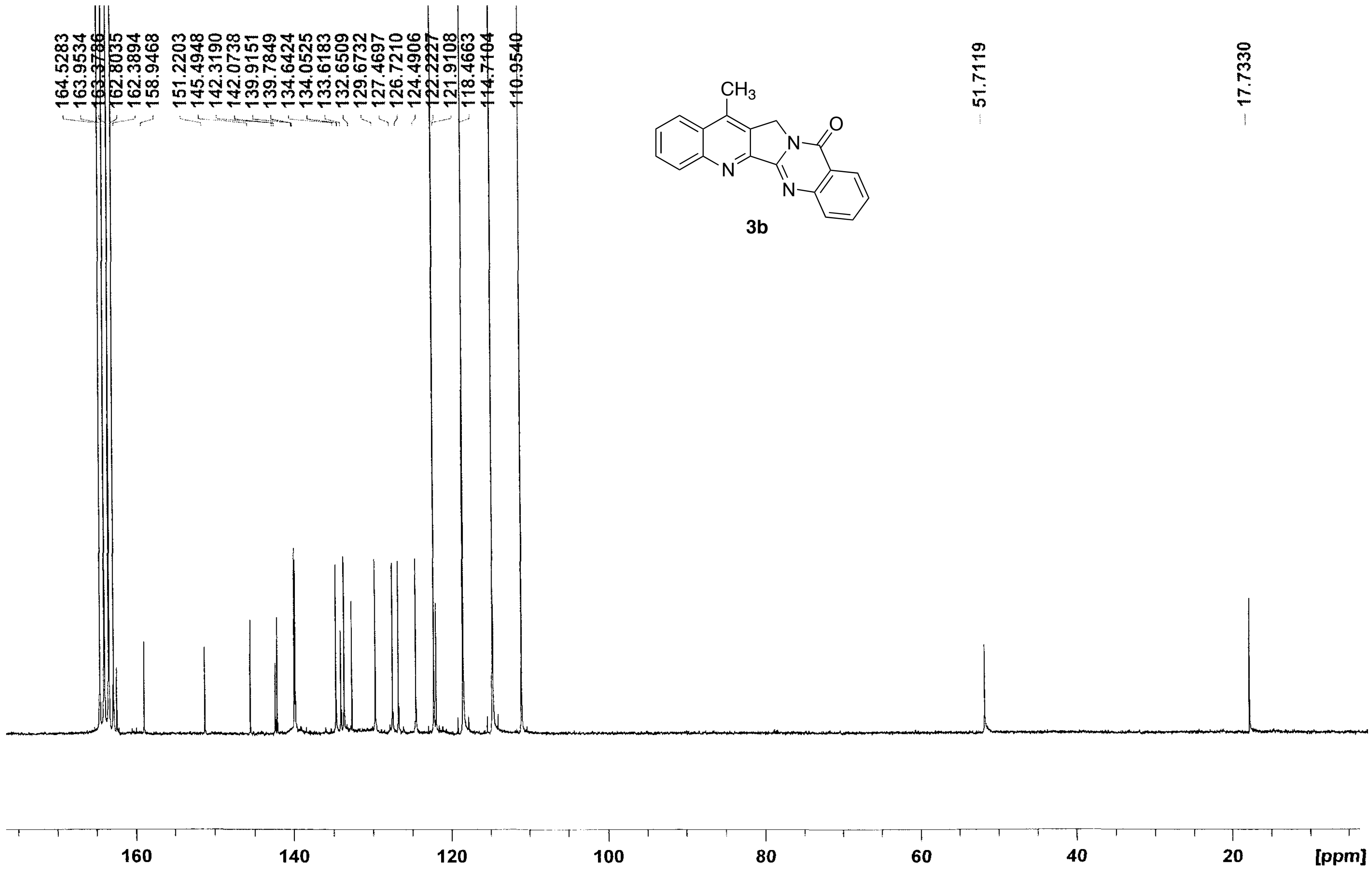
164.5283
163.9534
163.3786
162.8035
162.3894
158.9468
151.2203
145.4948
142.3190
142.0738
139.9151
139.7849
134.6424
134.0525
133.6183
132.6509
129.6732
127.4697
126.7210
124.4906
122.2227
121.9108
118.4663
114.7404
110.9540
51.7119
17.7330
CH3
O
N
N
N
3b
160
140
120
100
80
60
40
20
[ppm]

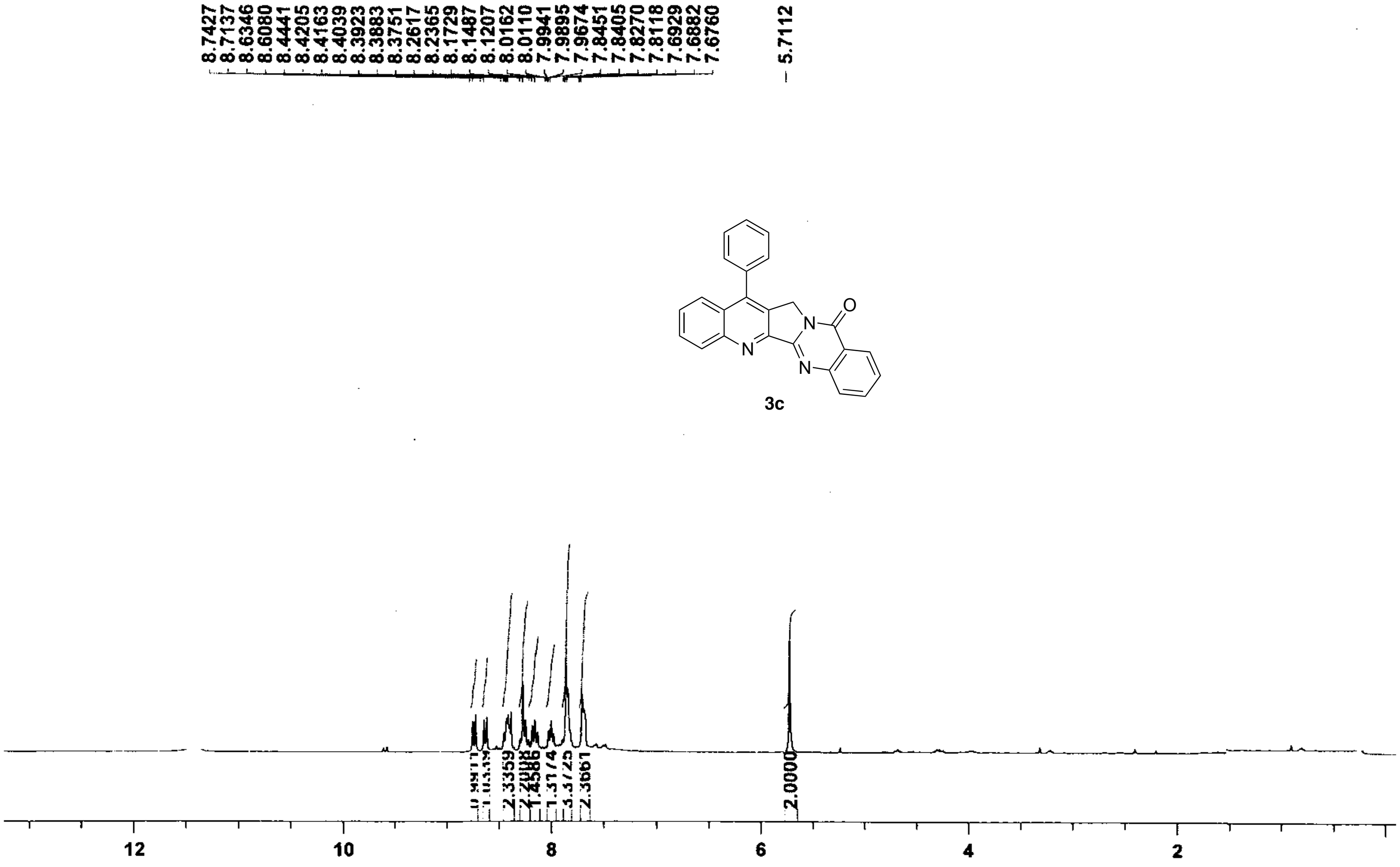
8.7427
8.7137
8.6346
8.6080
8.4441
8.4205
8.4163
8.4039
8.3923
8.3883
8.3751
8.2617
8.2365
8.1729
8.1487
8.1207
8.0162
8.0110
7.9941
7.9895
7.9674
7.8451
7.8405
7.8270
7.8118
7.6929
7.6882
7.6760
5.7112
3c
2.3359
2.3661
2.0000
12
10
8
6
4
2

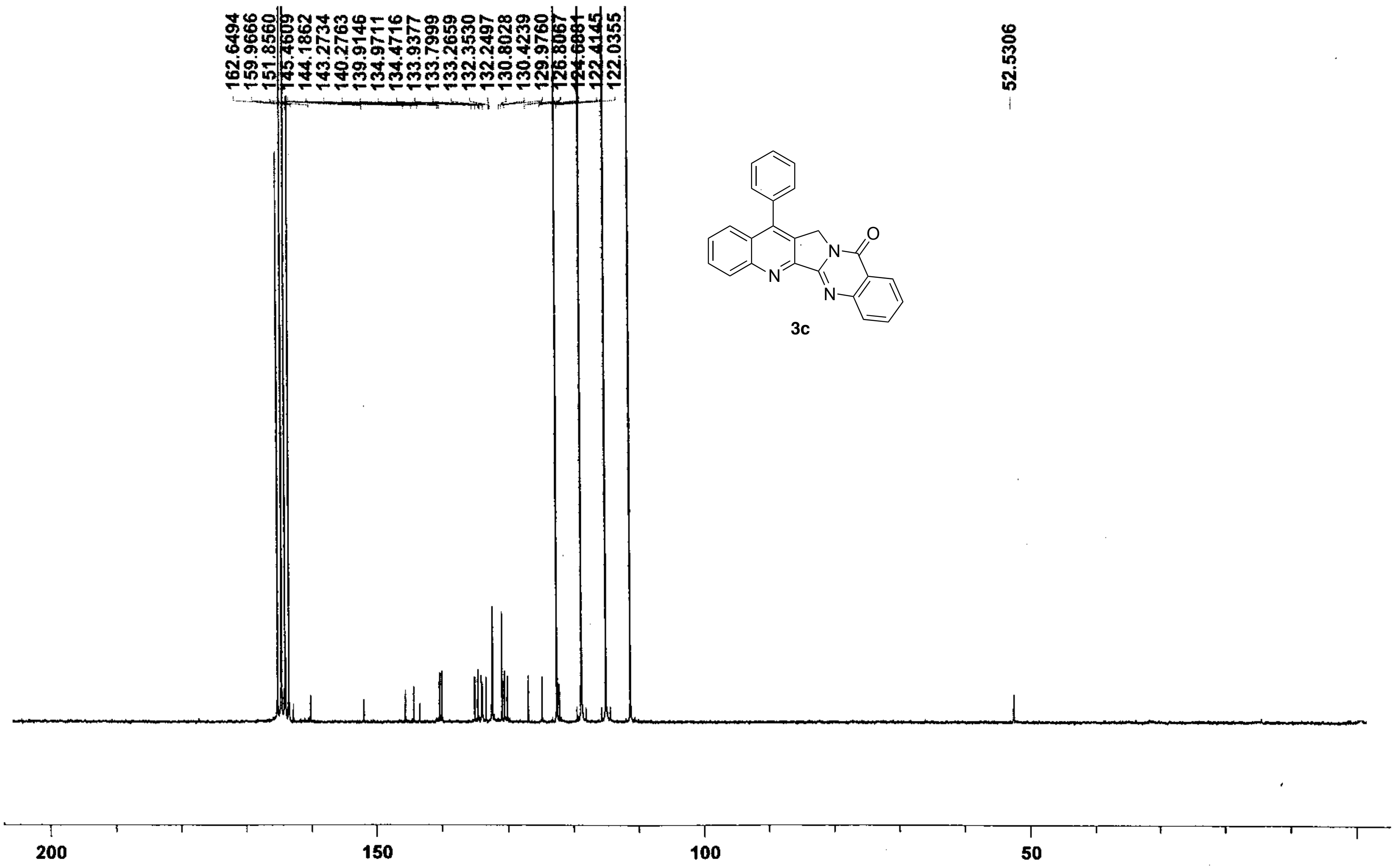
162.6494
159.9666
151.8560
145.4609
144.1862
143.2734
140.2763
139.9146
134.9711
134.4716
133.9377
133.7999
133.2659
132.3530
132.2497
130.8028
130.4239
129.9760
126.8067
124.6081
122.4145
122.0355
52.5306
O
N
N
N
3c
200
150
100
50

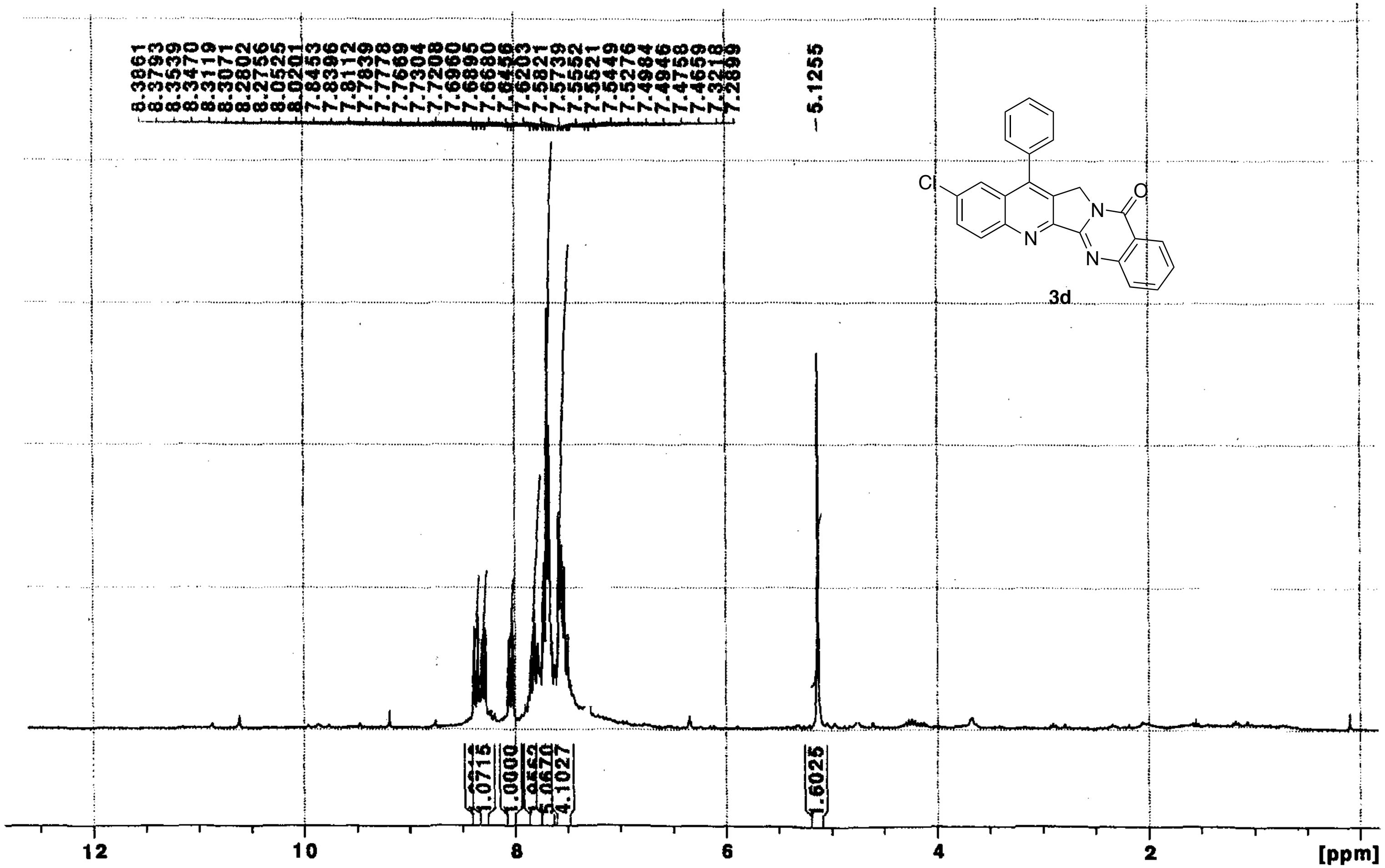
5.1255
Cl
N
O
3d
1.0000
4.1027
1.6025
12
10
8
6
4
2
[ppm]

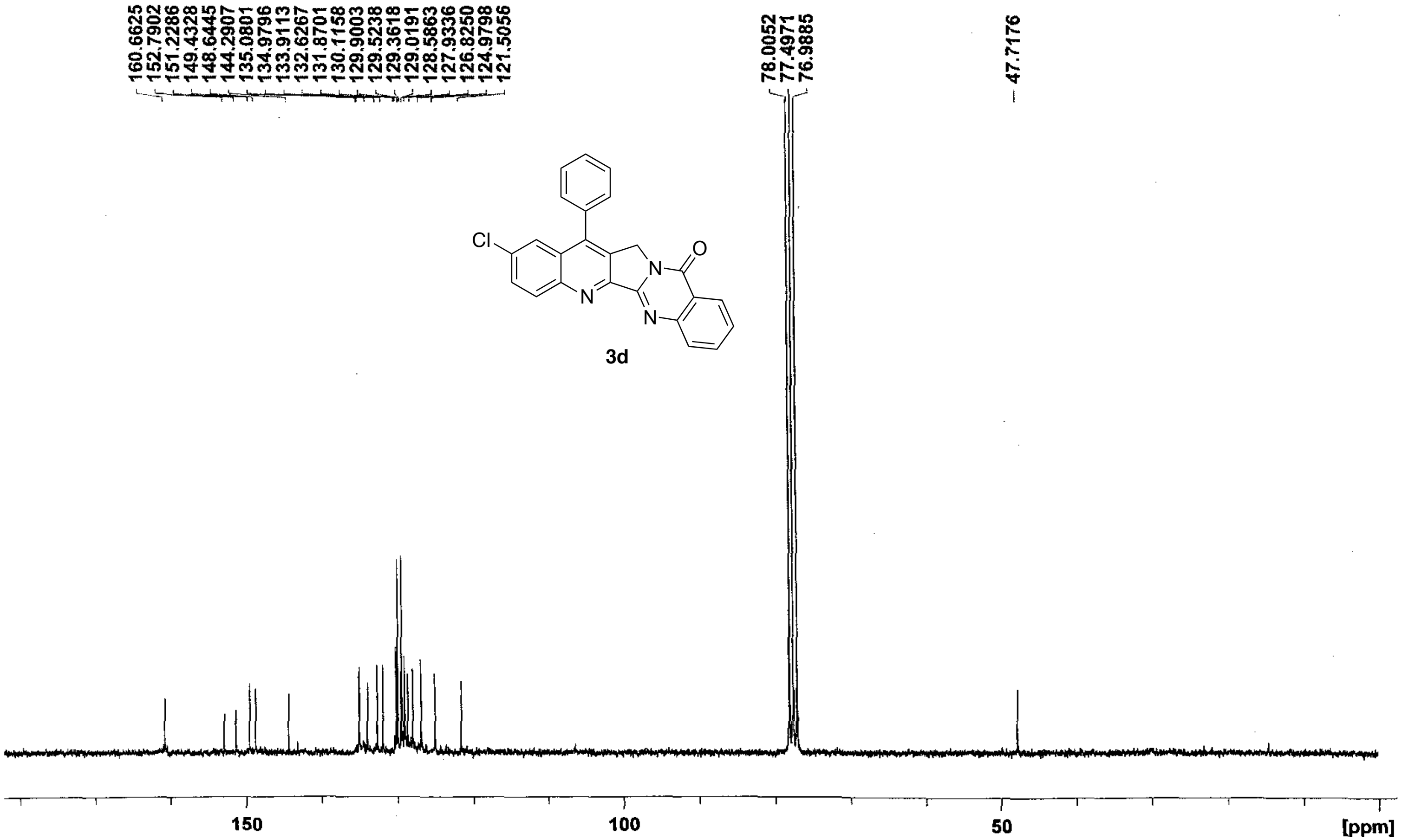

160.6625
152.7902
151.2286
149.4328
148.6445
144.2907
135.0801
134.9796
133.9113
132.6267
131.8701
130.1158
129.9003
129.5238
129.3618
129.0191
128.5863
127.9336
126.8250
124.9798
121.5056
78.0052
77.4971
76.9885
47.7176
Cl
N
N
N
O
3d
150
100
50
[ppm]

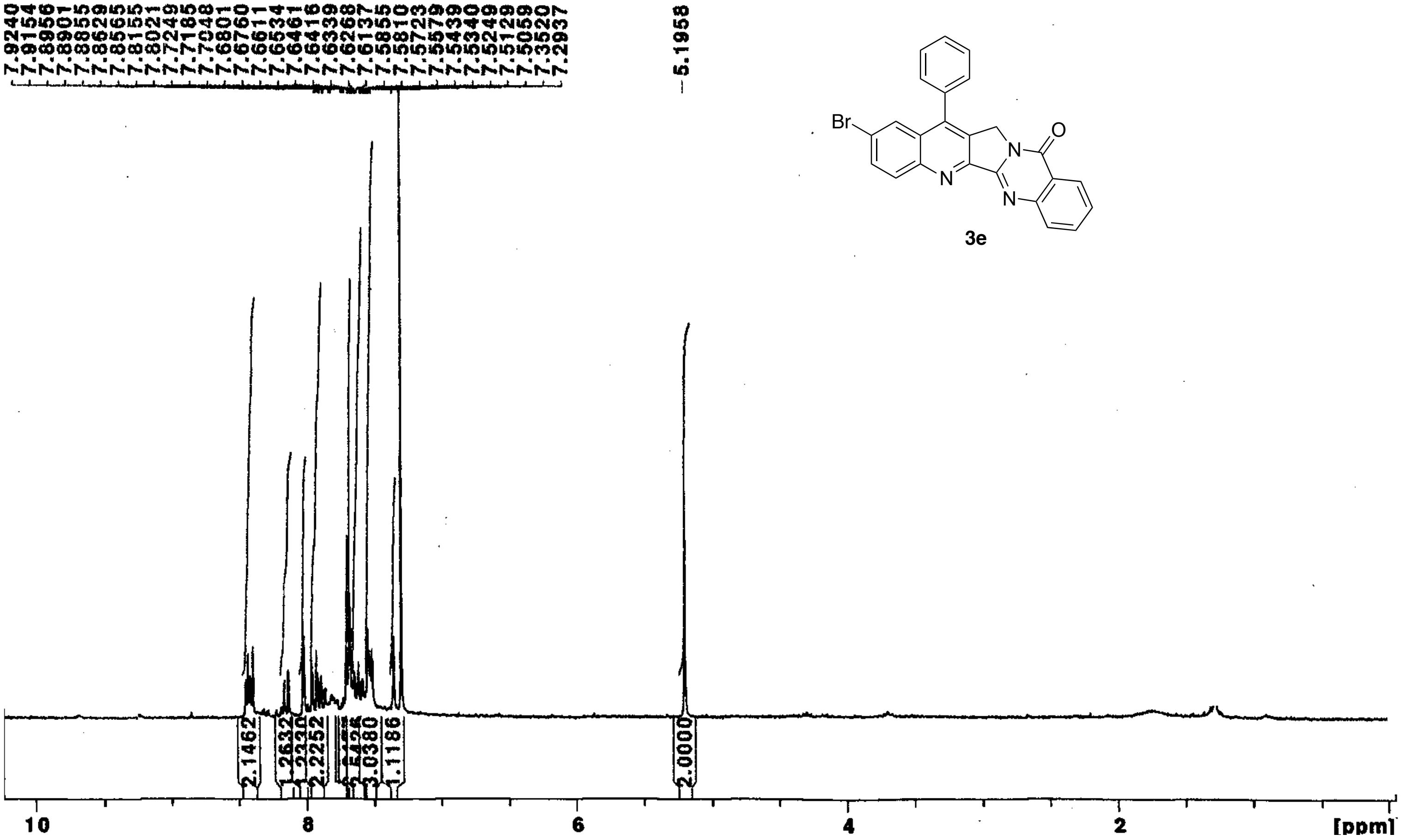

7.9240
7.9154
7.8956
7.8901
7.8855
7.8629
7.8565
7.8155
7.8021
7.7249
7.7185
7.7048
7.6801
7.6760
7.6611
7.6534
7.6461
7.6416
7.6339
7.6268
7.6137
7.5855
7.5810
7.5723
7.5579
7.5439
7.5340
7.5249
7.5129
7.5059
7.3520
7.2937
5.1958
Br
N
N
N
O
3e
2.1462
1.2632
1.2330
2.2252
3.0380
1.1186
2.0000
10
8
6
4
2
[ppm]

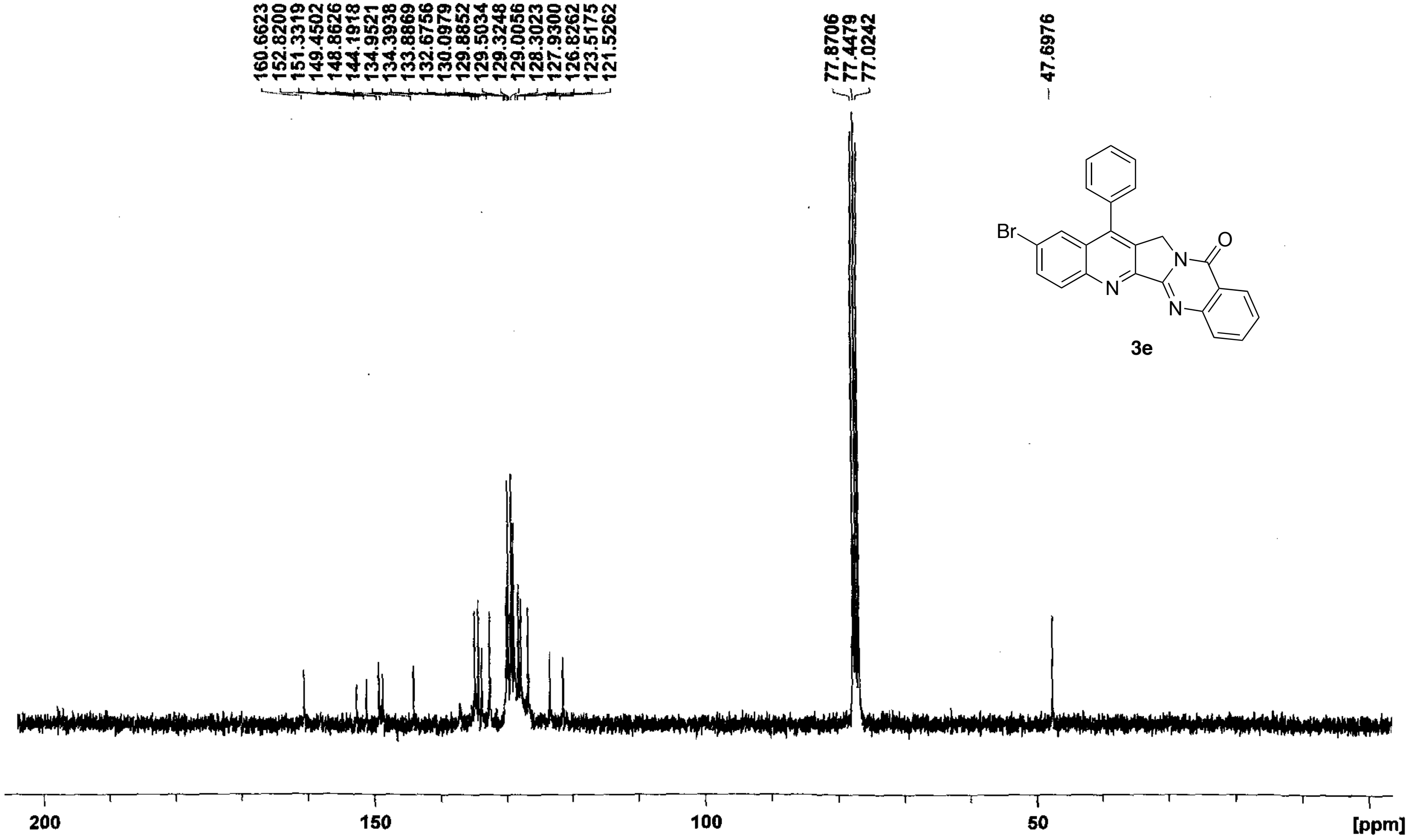
160.6623
152.8200
151.3319
149.4502
148.8626
144.1918
134.9521
134.3938
133.8869
132.6756
130.0979
129.8852
129.5034
129.3248
129.0056
128.3023
127.9300
126.8262
123.5175
121.5262
77.8706
77.4479
77.0242
47.6976
Br
N
O
3e
200
150
100
50
[ppm]

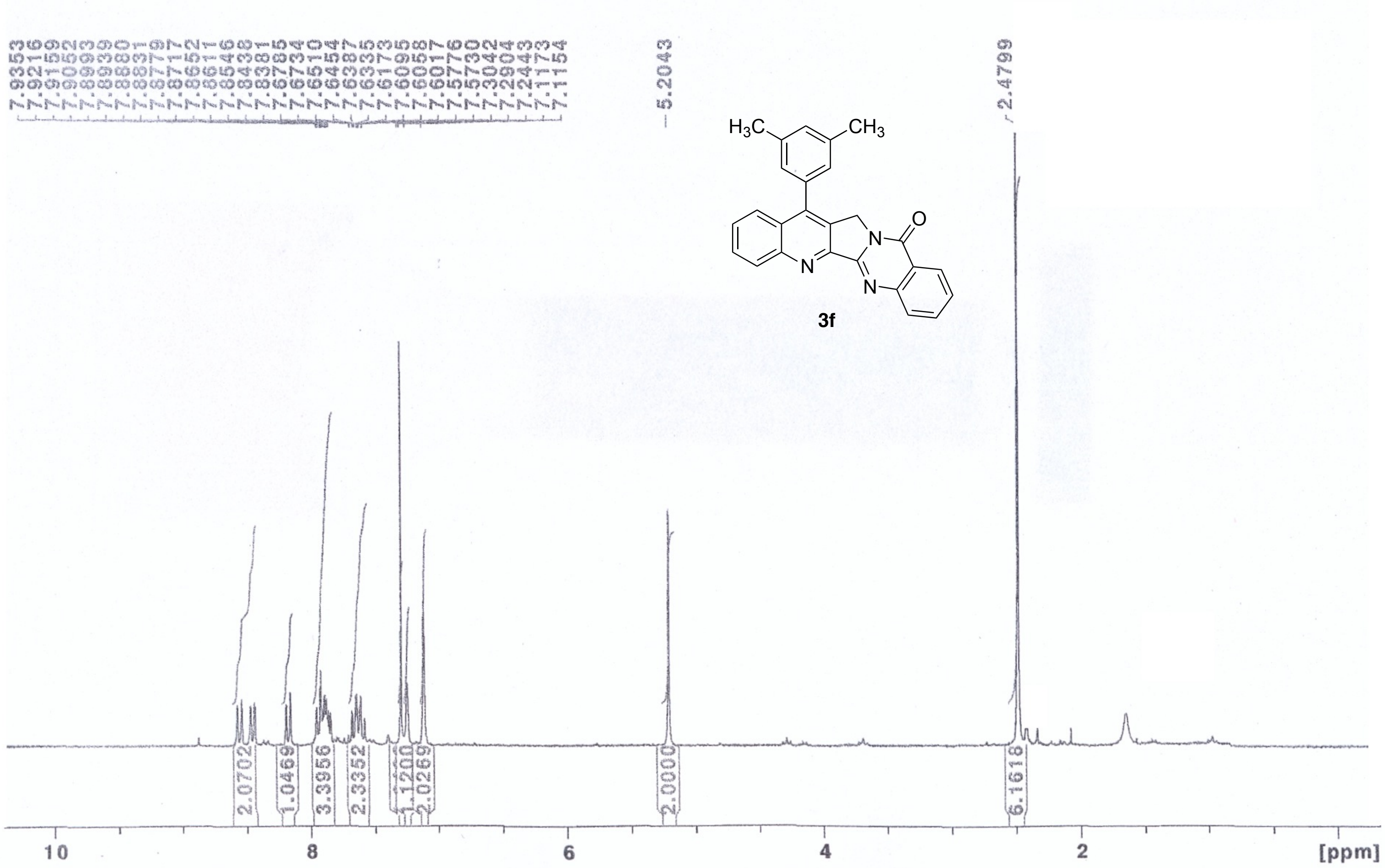
7.9353
7.9216
7.9159
7.9052
7.8993
7.8939
7.8880
7.8831
7.8779
7.8717
7.8652
7.8611
7.8546
7.8438
7.8381
7.6785
7.6734
7.6510
7.6454
7.6387
7.6335
7.6173
7.6095
7.6058
7.6017
7.5776
7.5730
7.3042
7.2904
7.2443
7.1173
7.1154
5.2043
2.4799
H3C
CH3
O
N
N
N
3f
2.0702
1.0469
3.3956
2.3352
1.1200
2.0269
2.0000
6.1618
10
8
6
4
2
[ppm]

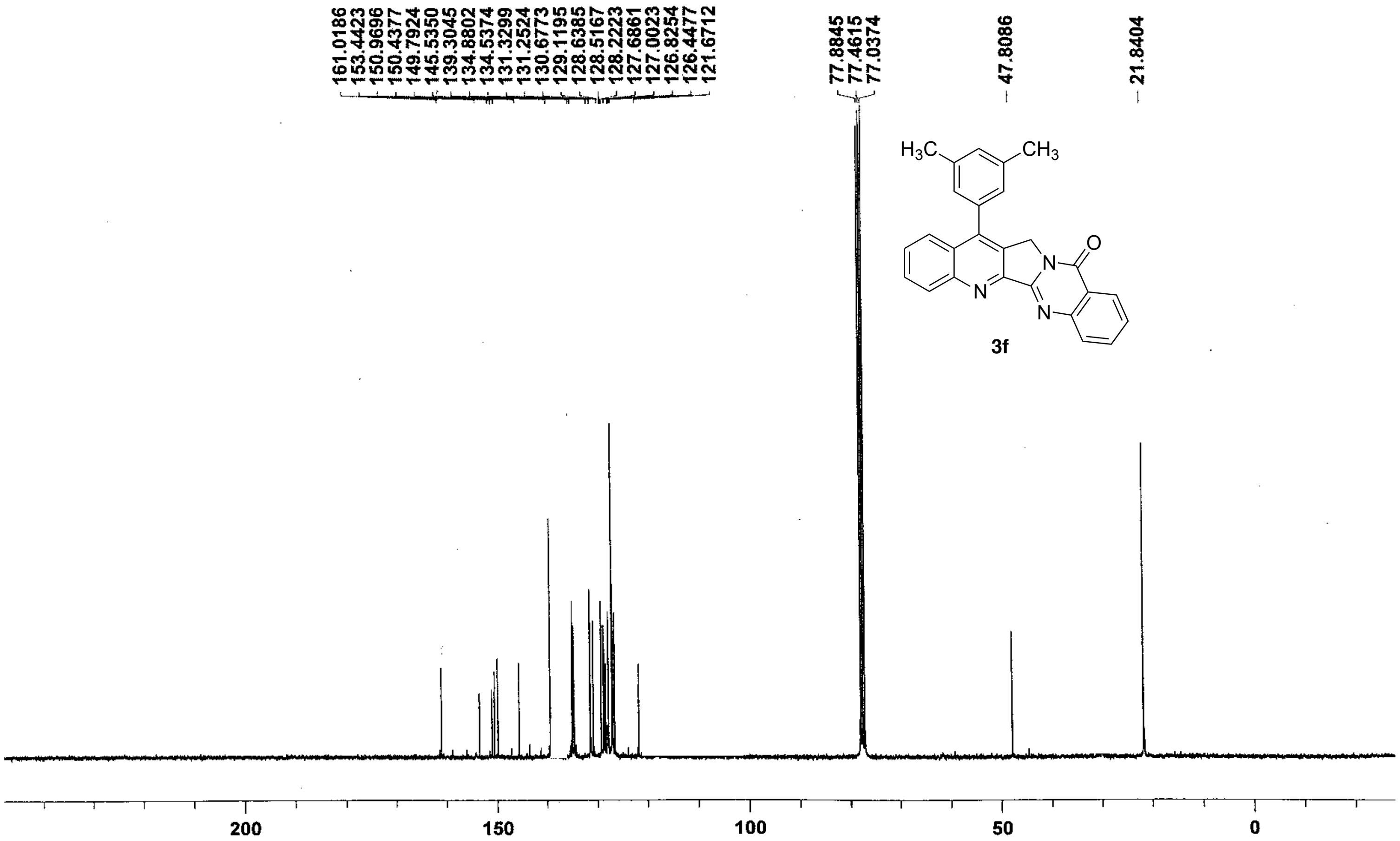
161.0186
153.4423
150.9696
150.4377
149.7924
145.5350
139.3045
134.8802
134.5374
131.3299
131.2524
130.6773
129.1195
128.6385
128.5167
128.2223
127.6861
127.0023
126.8254
126.4477
121.6712
77.8845
77.4615
77.0374
47.8086
21.8404
H_3C
CH_3
O
N
N
N
3f
200
150
100
50
0

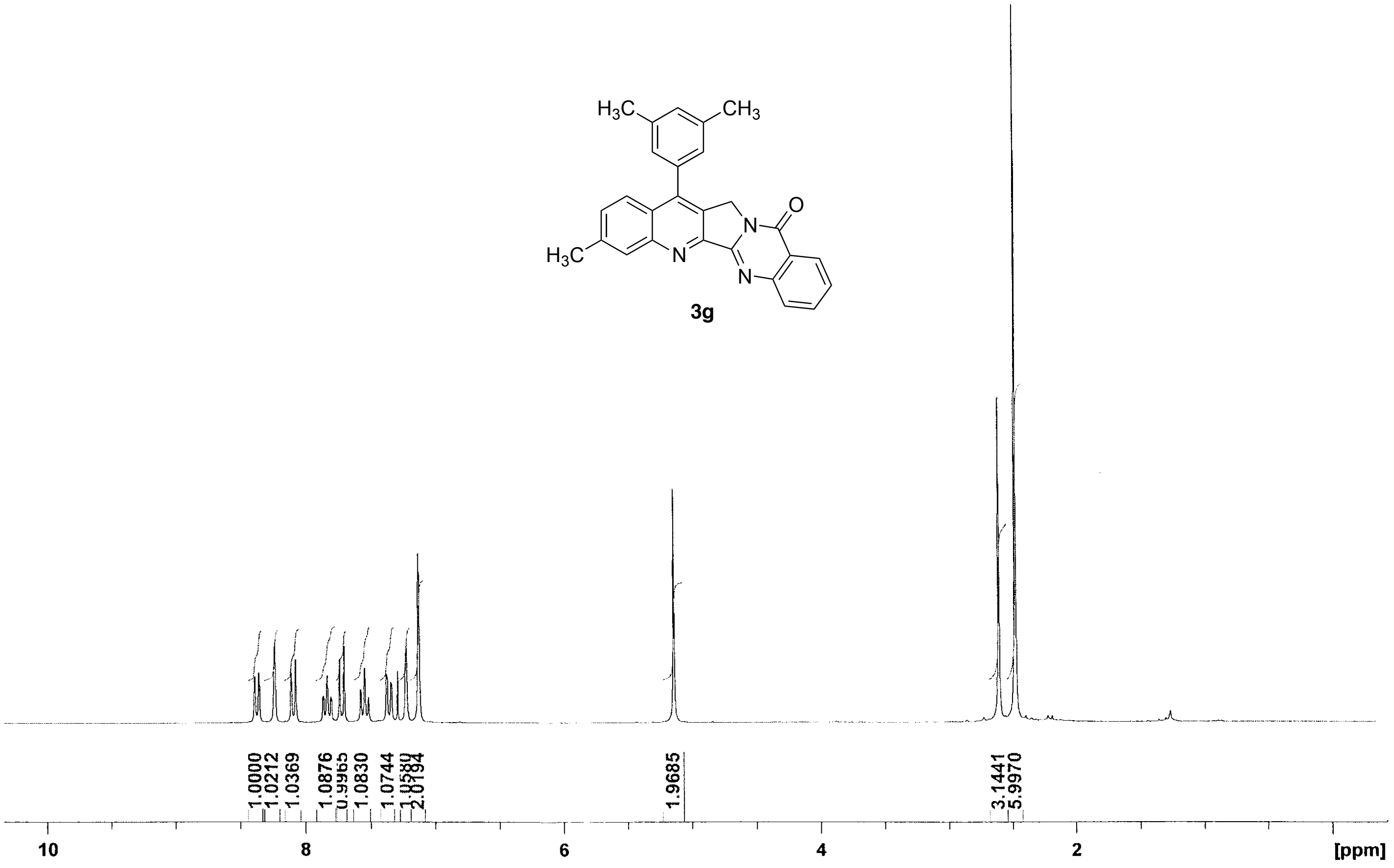
H3C
CH3
H3C
N
N
N
O
3g
1.0000
1.0212
1.0369
1.0876
0.9965
1.0830
1.0744
1.0580
2.0194
1.9685
3.1441
5.9970
10
8
6
4
2
[ppm]

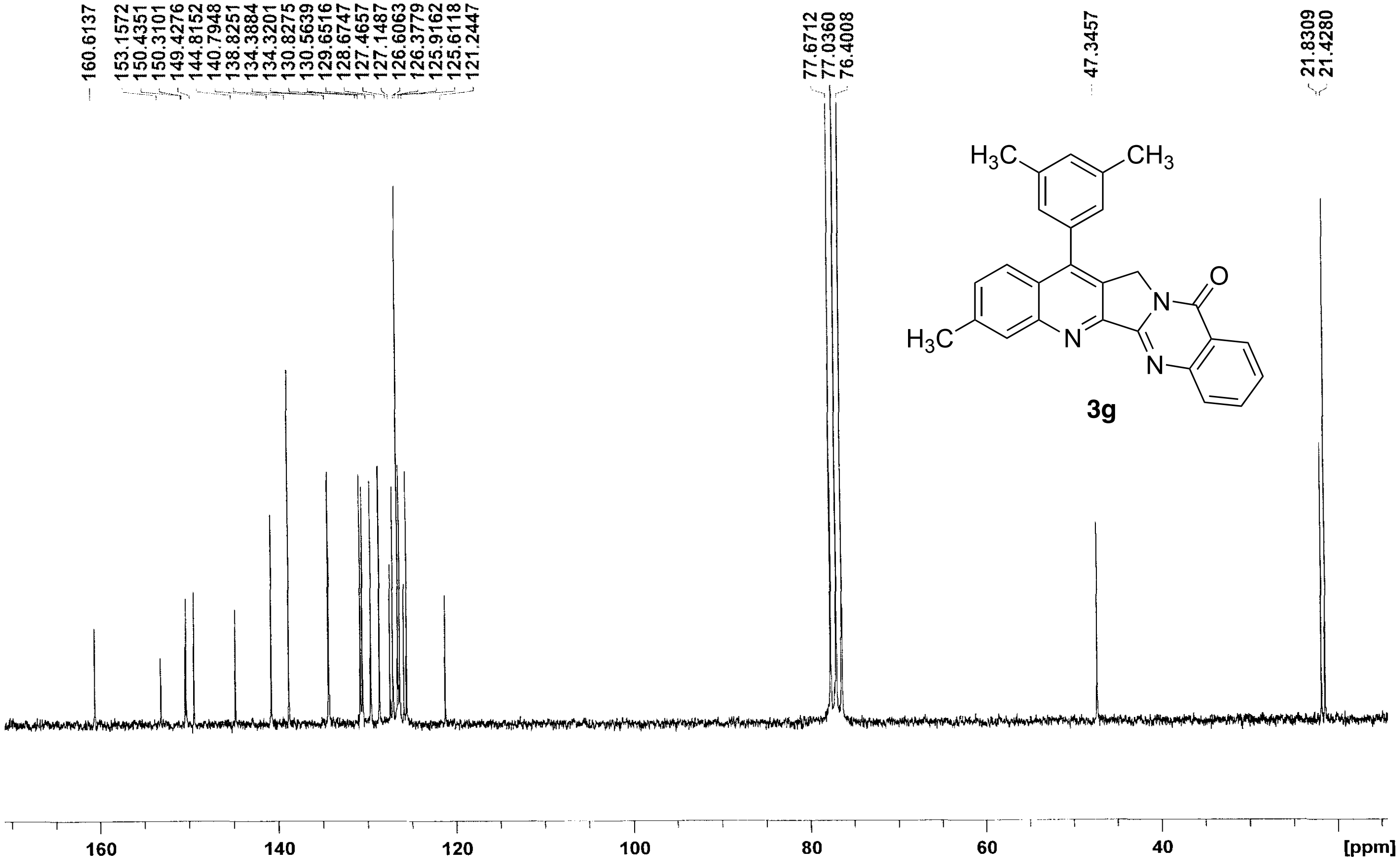
160.6137
153.1572
150.4351
150.3101
149.4276
144.8152
140.7948
138.8251
134.3884
134.3201
130.8275
130.5639
129.6516
128.6747
127.4657
127.1487
126.6063
126.3779
125.9162
125.6118
121.2447
77.6712
77.0360
76.4008
47.3457
21.8309
21.4280
H_3C
CH_3
O
N
N
N
H_3C
3g
160
140
120
100
80
60
40
[ppm]

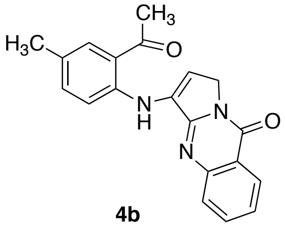
CH3
H3C
NH
N
O

4b

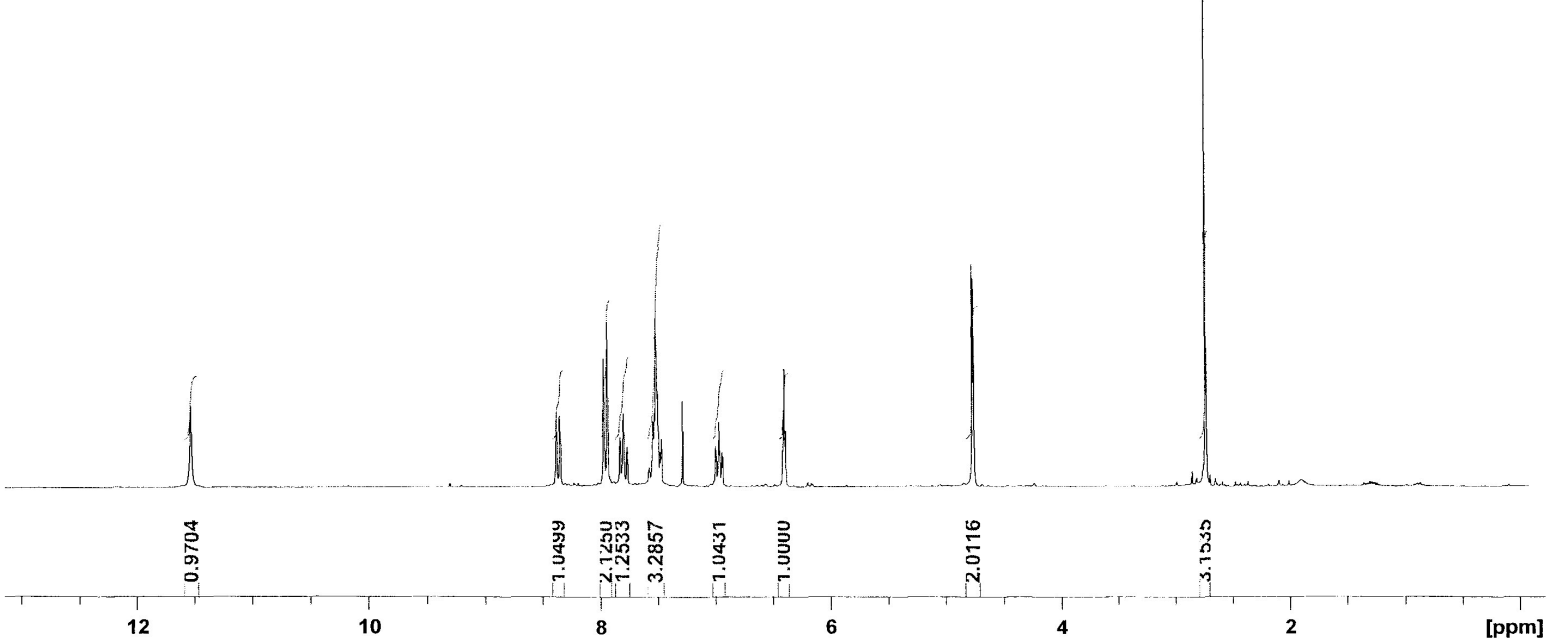
0.9704
1.0499
2.1250
1.2533
3.2857
1.0431
1.0000
2.0116
3.1535
12
10
8
6
4
2
[ppm]

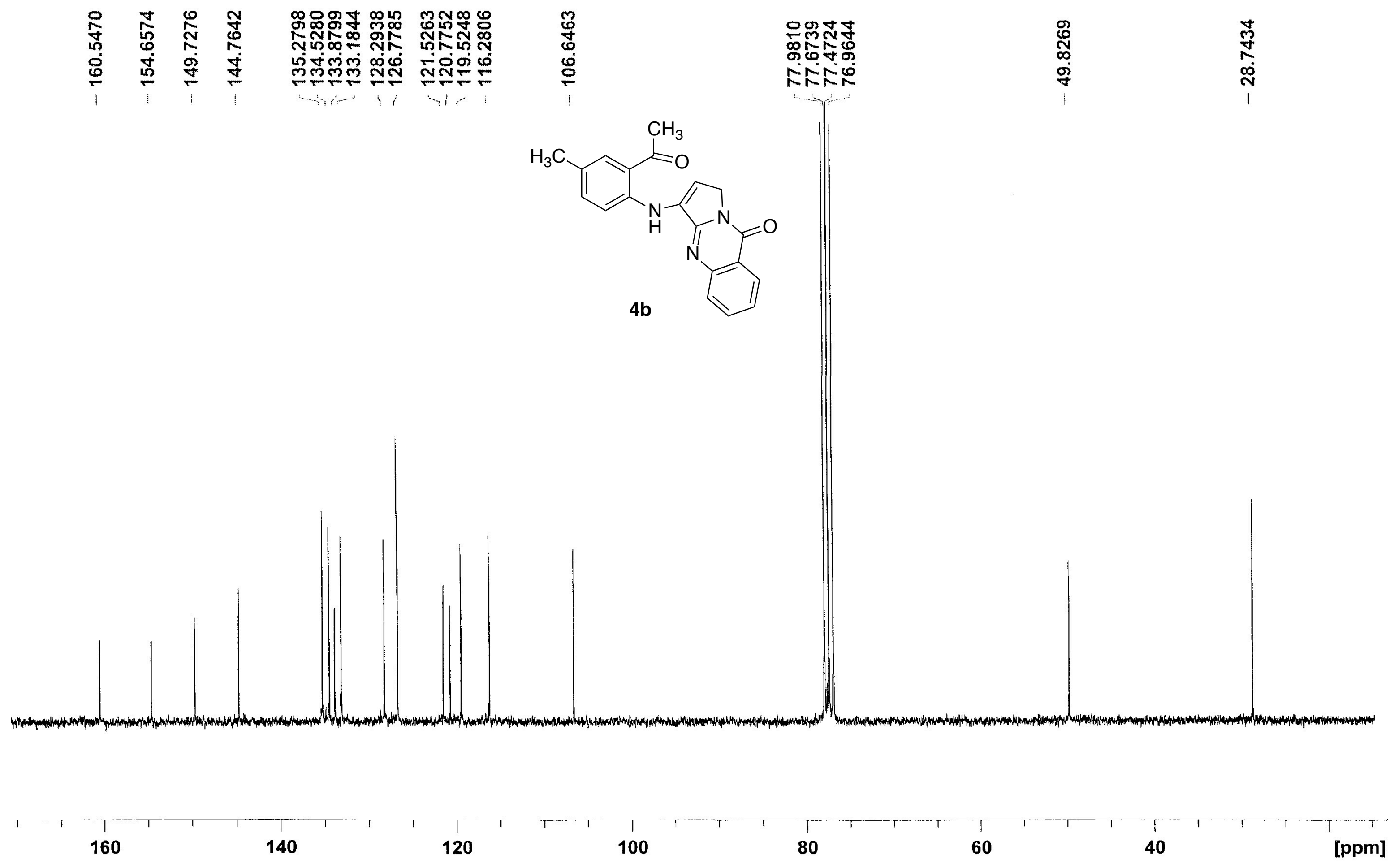
160.5470
154.6574
149.7276
144.7642
135.2798
134.5280
133.8799
133.1844
128.2938
126.7785
121.5263
120.7752
119.5248
116.2806
106.6463
77.9810
77.6739
77.4724
76.9644
49.8269
28.7434
H3C
CH3
O
N
H
N
N
O
4b
160
140
120
100
80
60
40
[ppm]

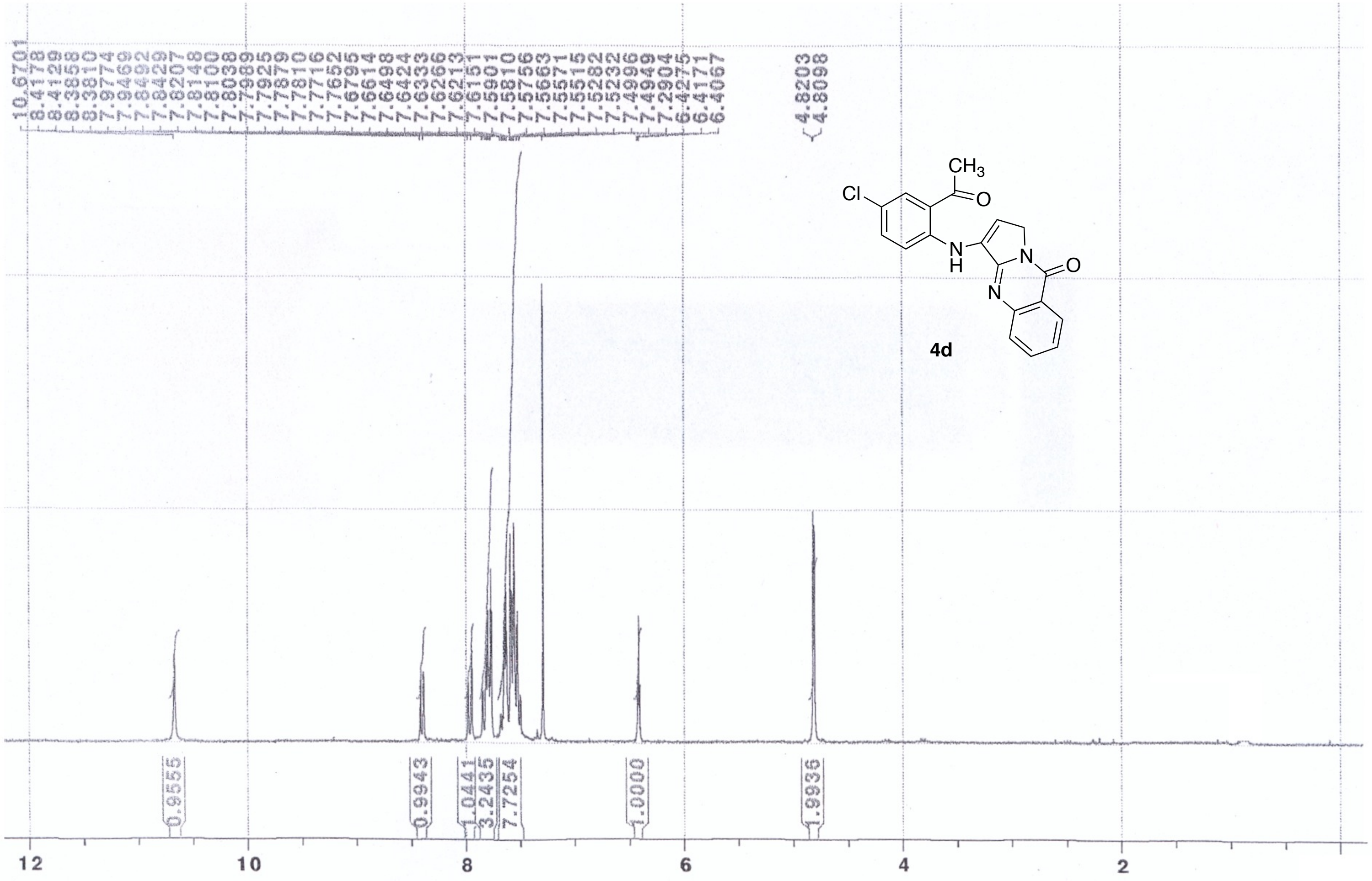
10.6701
8.4178
8.4129
8.3858
8.3810
7.9774
7.9469
7.8492
7.8429
7.8207
7.8148
7.8100
7.8038
7.7989
7.7925
7.7879
7.7810
7.7716
7.7652
7.6795
7.6614
7.6498
7.6424
7.6333
7.6266
7.6213
7.6151
7.5901
7.5810
7.5756
7.5663
7.5571
7.5515
7.5282
7.5232
7.4996
7.4949
7.2904
6.4275
6.4171
6.4067
4.8203
4.8098
CH3
Cl
O
NH
N
N
O
4d
0.9555
0.9943
1.0441
3.2435
7.7254
1.0000
1.9936
12
10
8
6
4
2

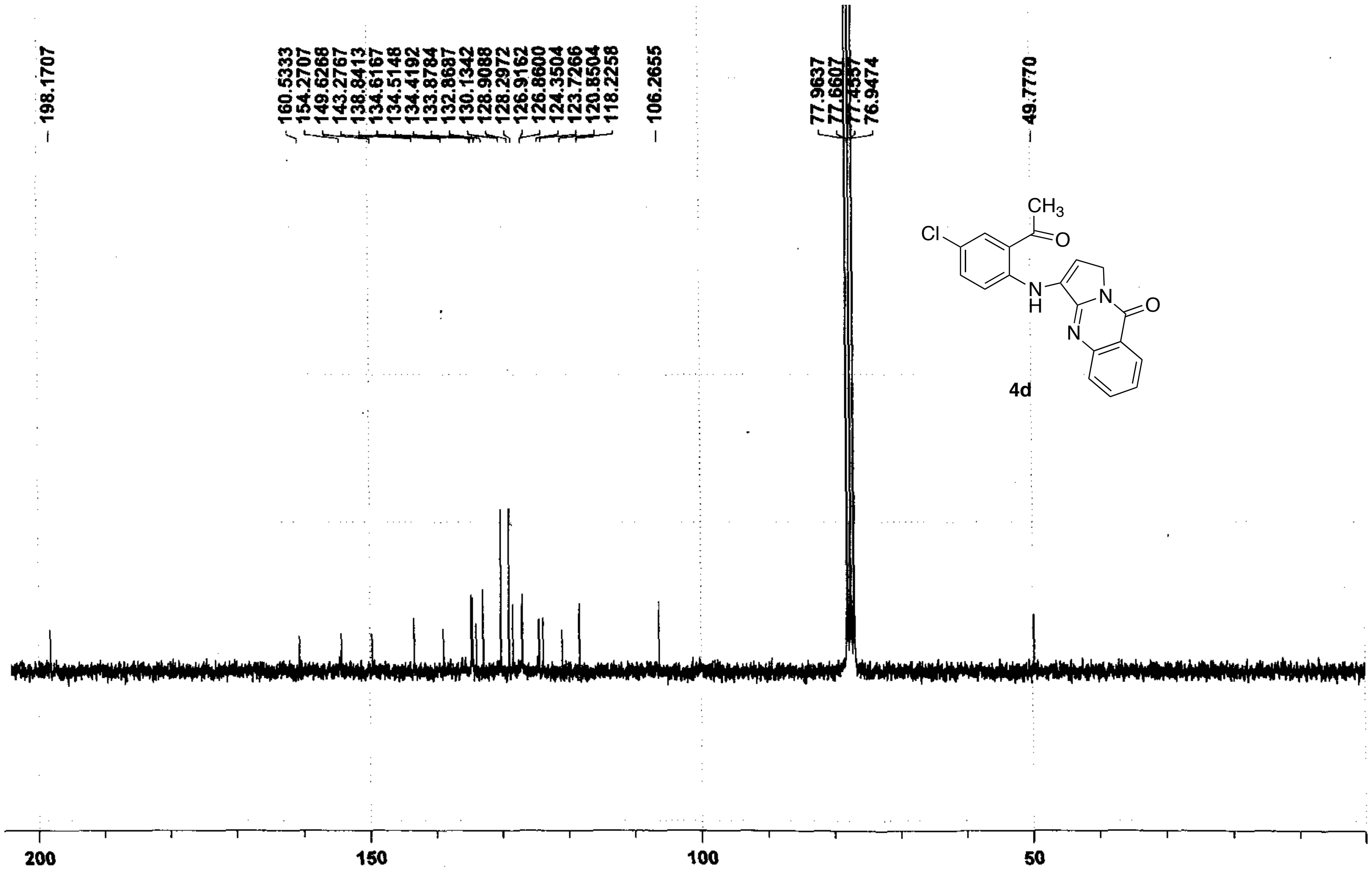
198.1707
160.5333
154.2707
149.6268
143.2767
138.8413
134.6167
134.5148
134.4192
133.8784
132.8687
130.1342
128.9088
128.2972
126.9162
126.8600
124.3504
123.7266
120.8504
118.2258
106.2655
77.9637
77.6607
77.4557
76.9474
49.7770
CH3
Cl
O
N
H
N
N
O
4d
200
150
100
50
