# Supplementary figures and images for: B-Ring-Aryl Substituted Luotonin A Analogues with a New Binding Mode to the Topoisomerase 1-DNA Complex Show Enhanced Cytotoxic Activity
Source: PLoS One. 2014 May 15;9(5):e95998. doi: 10.1371/journal.pone.0095998 (PMC4022624; doi:10.1371/journal.pone.0095998)

**A**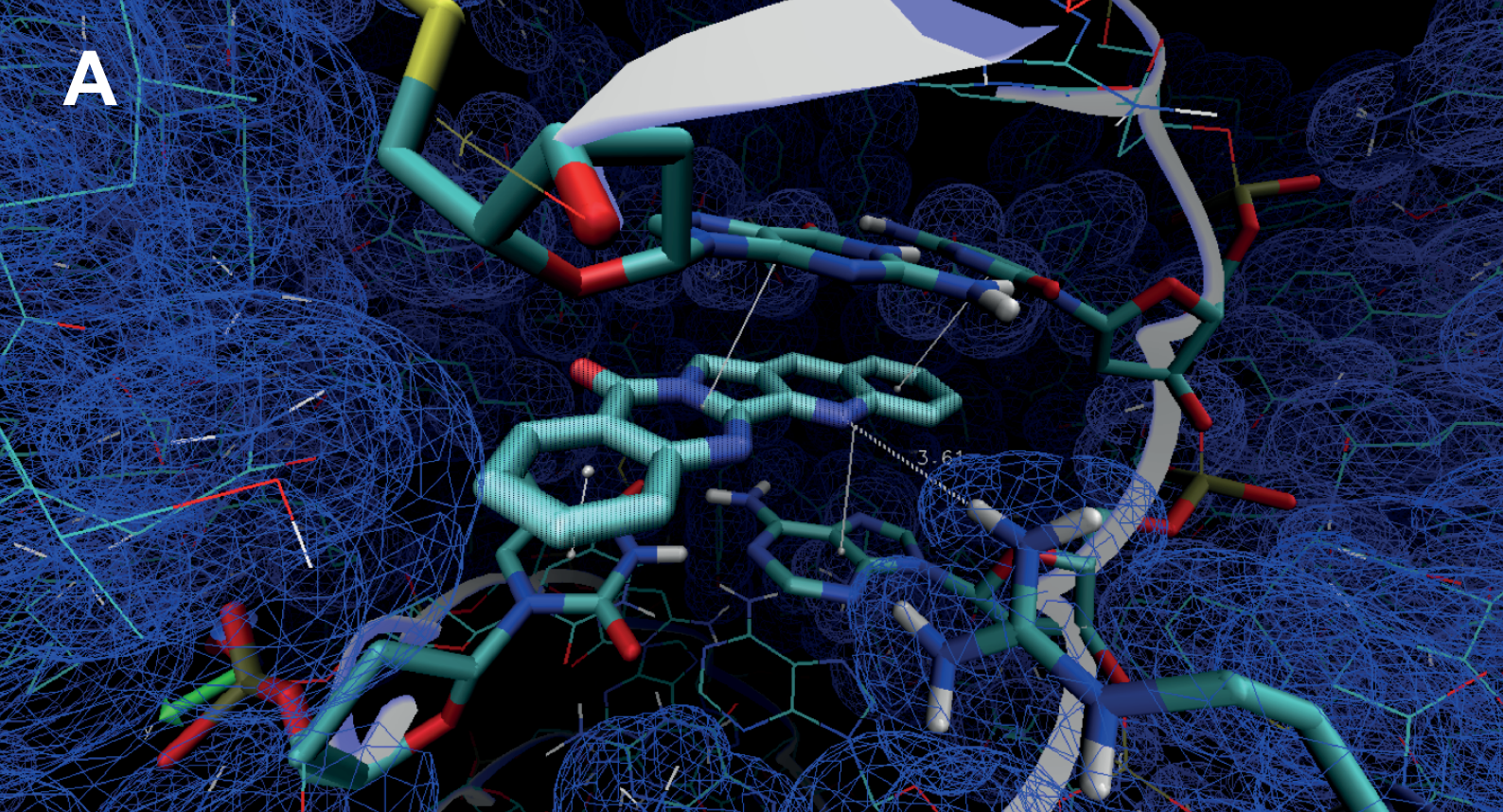**B**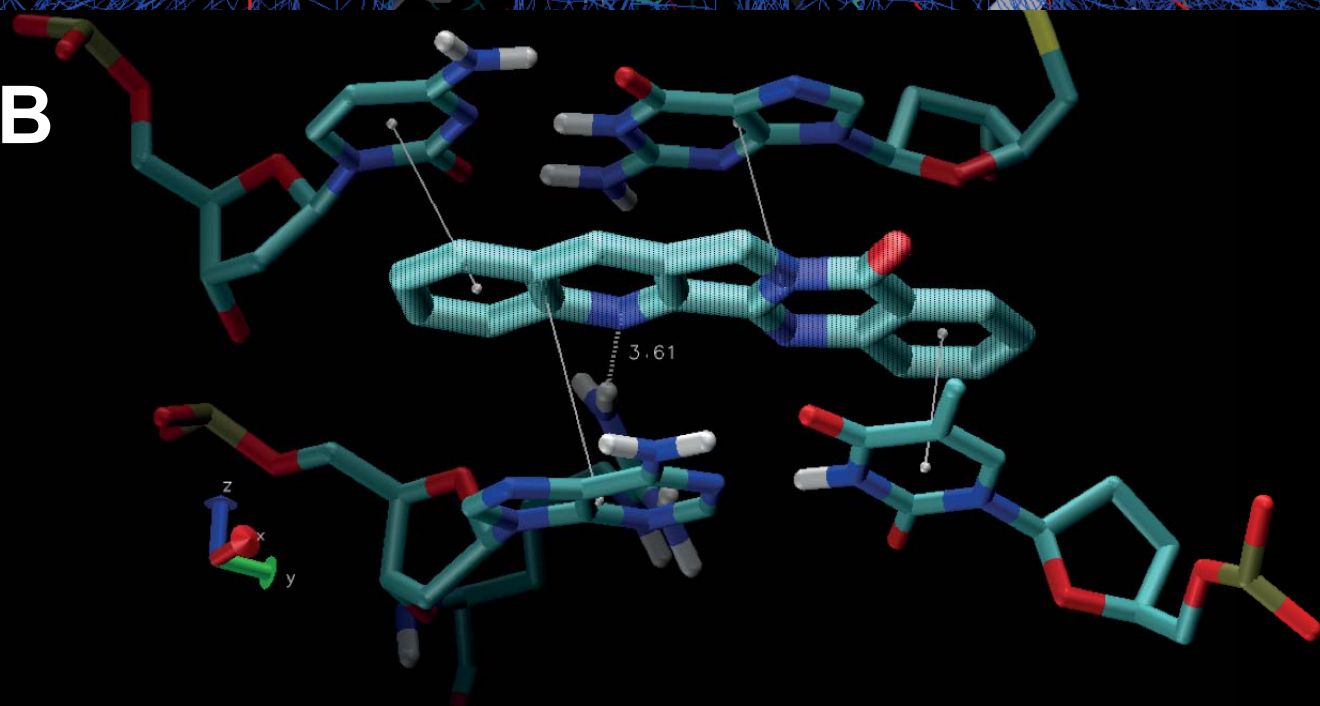

Supplement: File S1 — SI1, 1H and 13C–NMR spectra of all compounds. Figure S1, Two views of compound 3a (luotonin A) docked in the topoisomerase 1 active site. Figure S2, Two views of compound 3b docked in the topoisomerase 1 active site. Figure S3, Two views of compound 3c docked in the topoisomerase 1 active site. Figure S4, Two views of compound 3d docked in the topoisomerase 1 active site. Figure S5, Two views of compound 3e docked in the topoisomerase 1 active site. Figure S6, Two views of compound 3f docked in the topoisomerase 1 active site. Figure S7, Two views of compound 3g docked in the topoisomerase 1 active site. (ZIP) [file pone.0095998.s001.zip › Supporting info/FigureS1.pdf]

**A**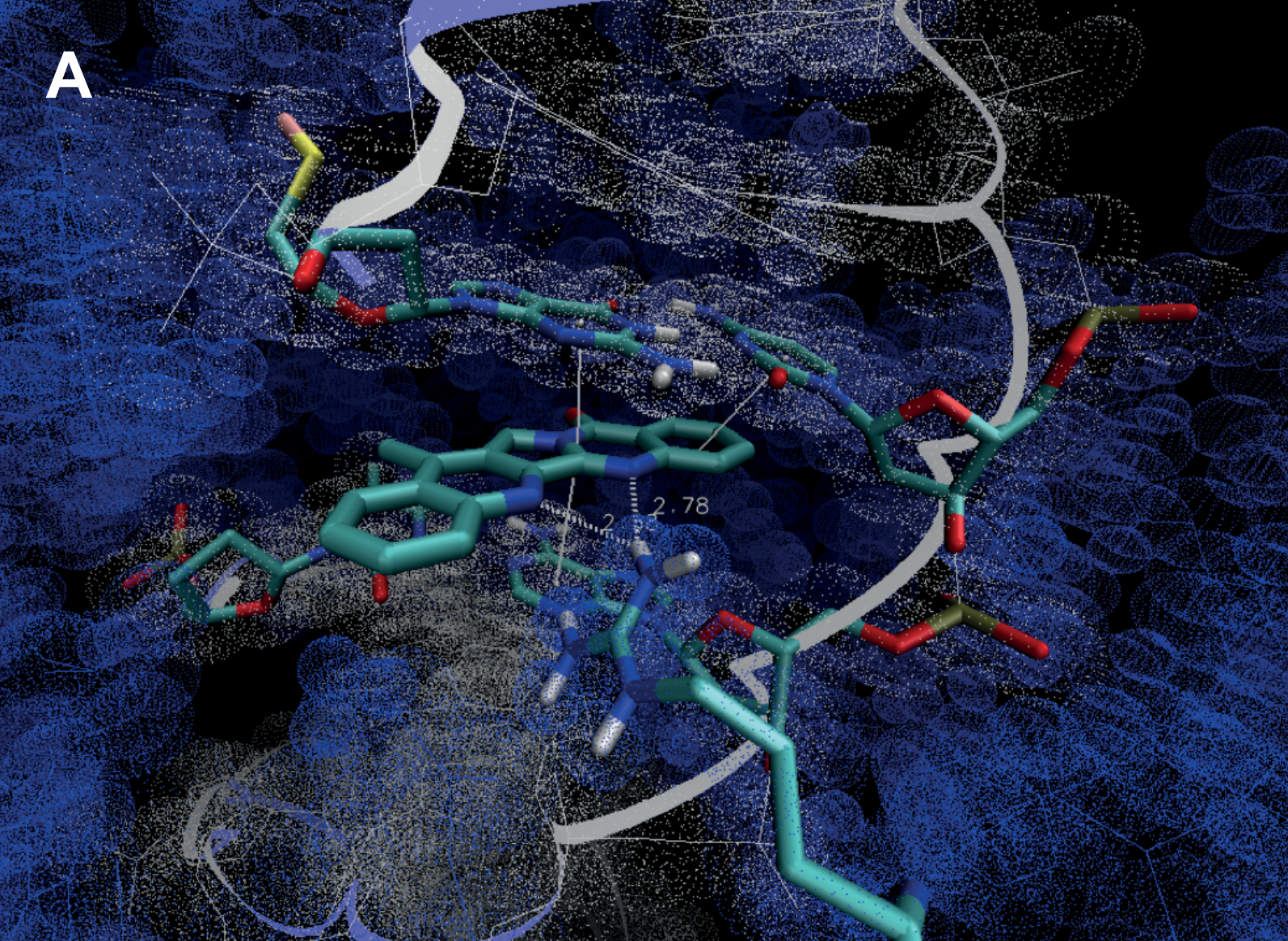**B**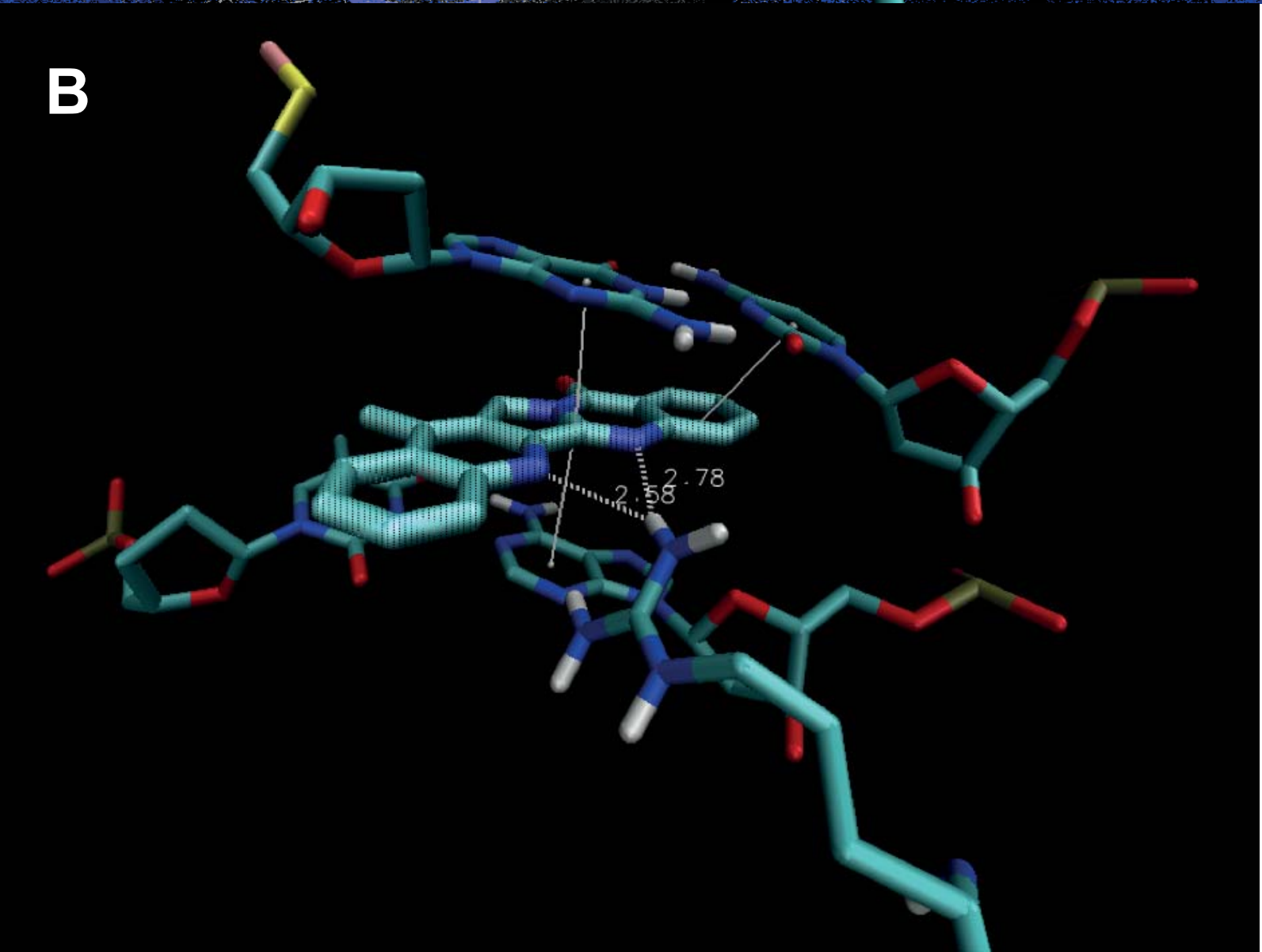

Supplement: File S1 — SI1, 1H and 13C–NMR spectra of all compounds. Figure S1, Two views of compound 3a (luotonin A) docked in the topoisomerase 1 active site. Figure S2, Two views of compound 3b docked in the topoisomerase 1 active site. Figure S3, Two views of compound 3c docked in the topoisomerase 1 active site. Figure S4, Two views of compound 3d docked in the topoisomerase 1 active site. Figure S5, Two views of compound 3e docked in the topoisomerase 1 active site. Figure S6, Two views of compound 3f docked in the topoisomerase 1 active site. Figure S7, Two views of compound 3g docked in the topoisomerase 1 active site. (ZIP) [file pone.0095998.s001.zip › Supporting info/FigureS2.pdf]

**A**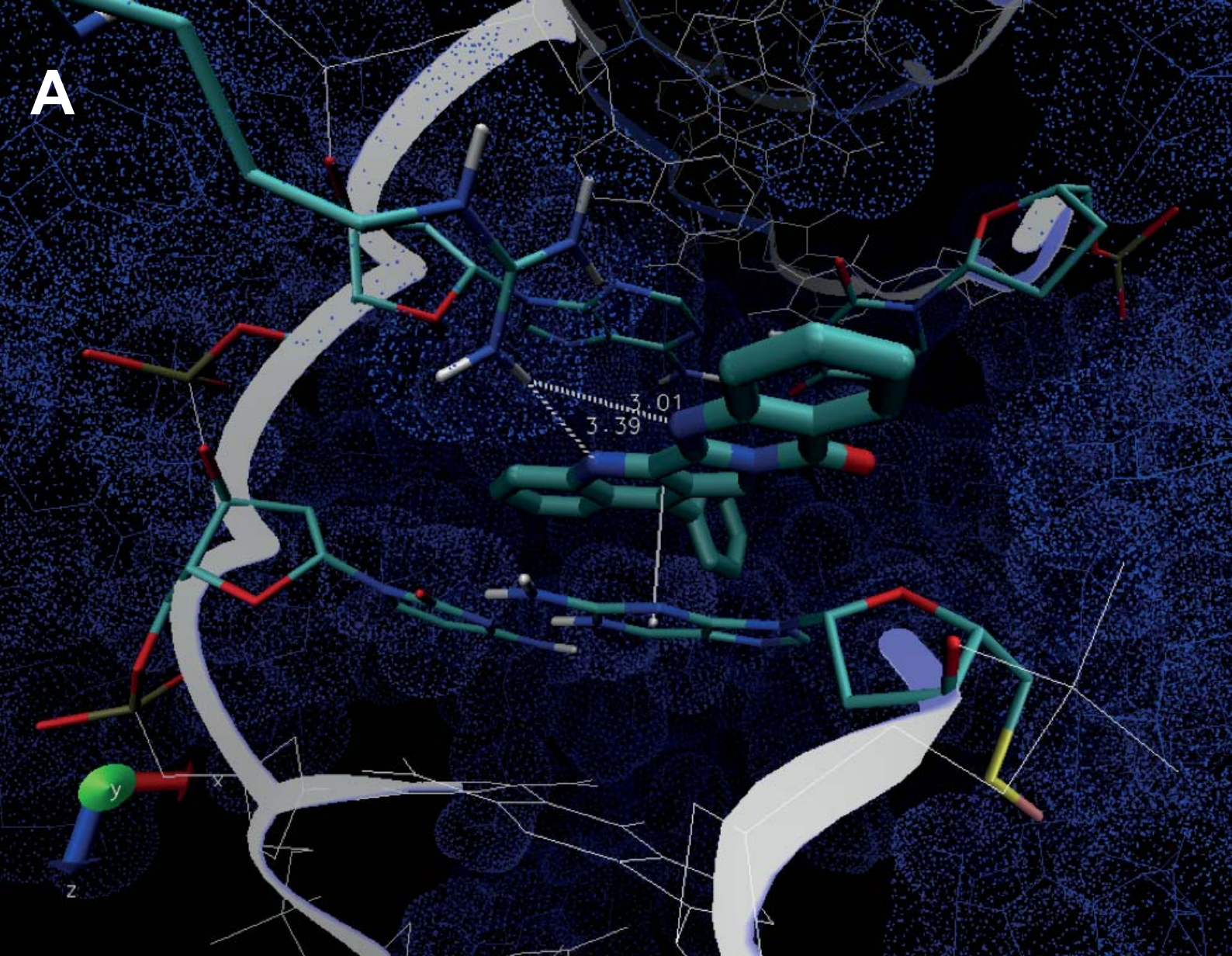**B**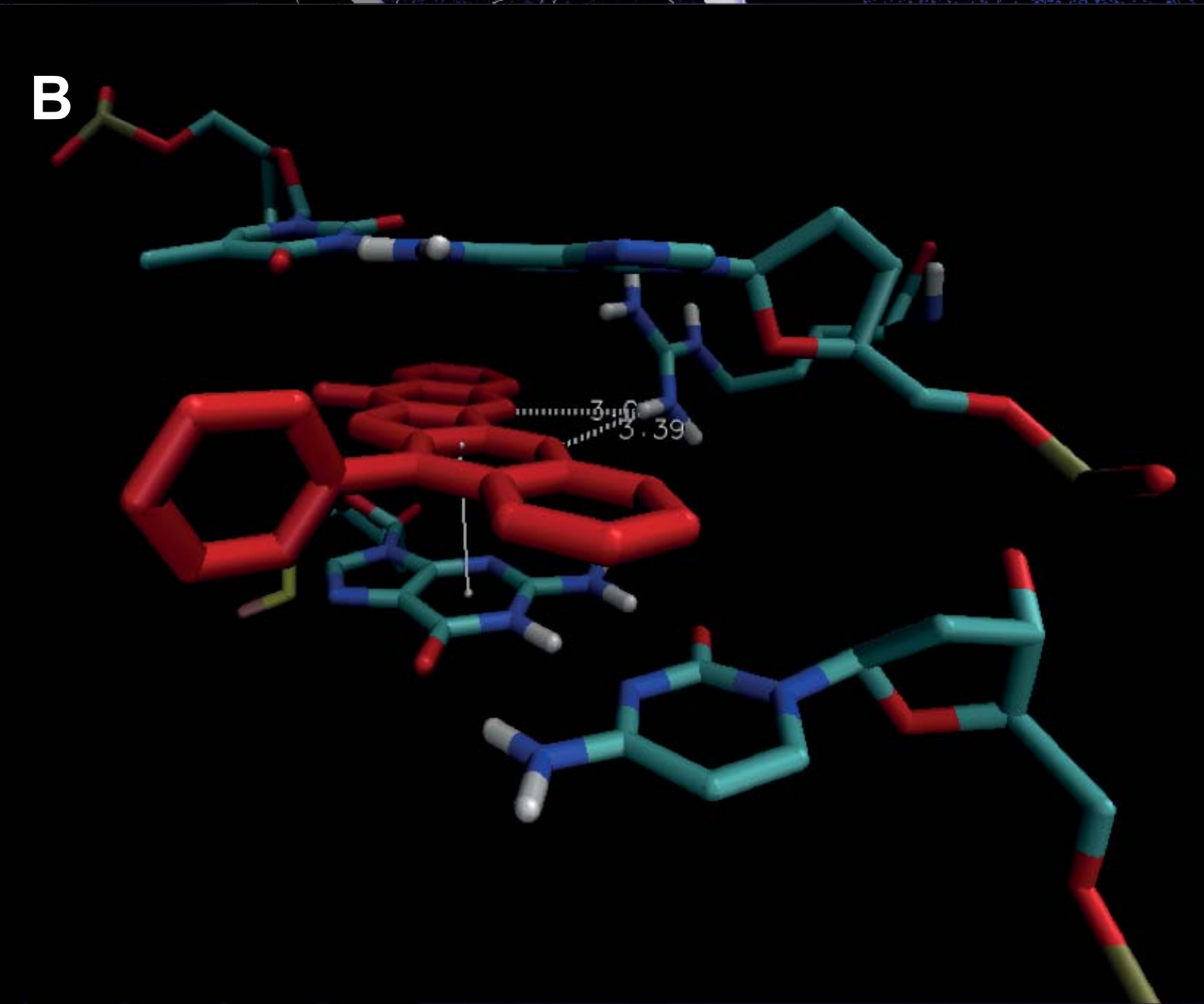

Supplement: File S1 — SI1, 1H and 13C–NMR spectra of all compounds. Figure S1, Two views of compound 3a (luotonin A) docked in the topoisomerase 1 active site. Figure S2, Two views of compound 3b docked in the topoisomerase 1 active site. Figure S3, Two views of compound 3c docked in the topoisomerase 1 active site. Figure S4, Two views of compound 3d docked in the topoisomerase 1 active site. Figure S5, Two views of compound 3e docked in the topoisomerase 1 active site. Figure S6, Two views of compound 3f docked in the topoisomerase 1 active site. Figure S7, Two views of compound 3g docked in the topoisomerase 1 active site. (ZIP) [file pone.0095998.s001.zip › Supporting info/FigureS3.pdf]

**A**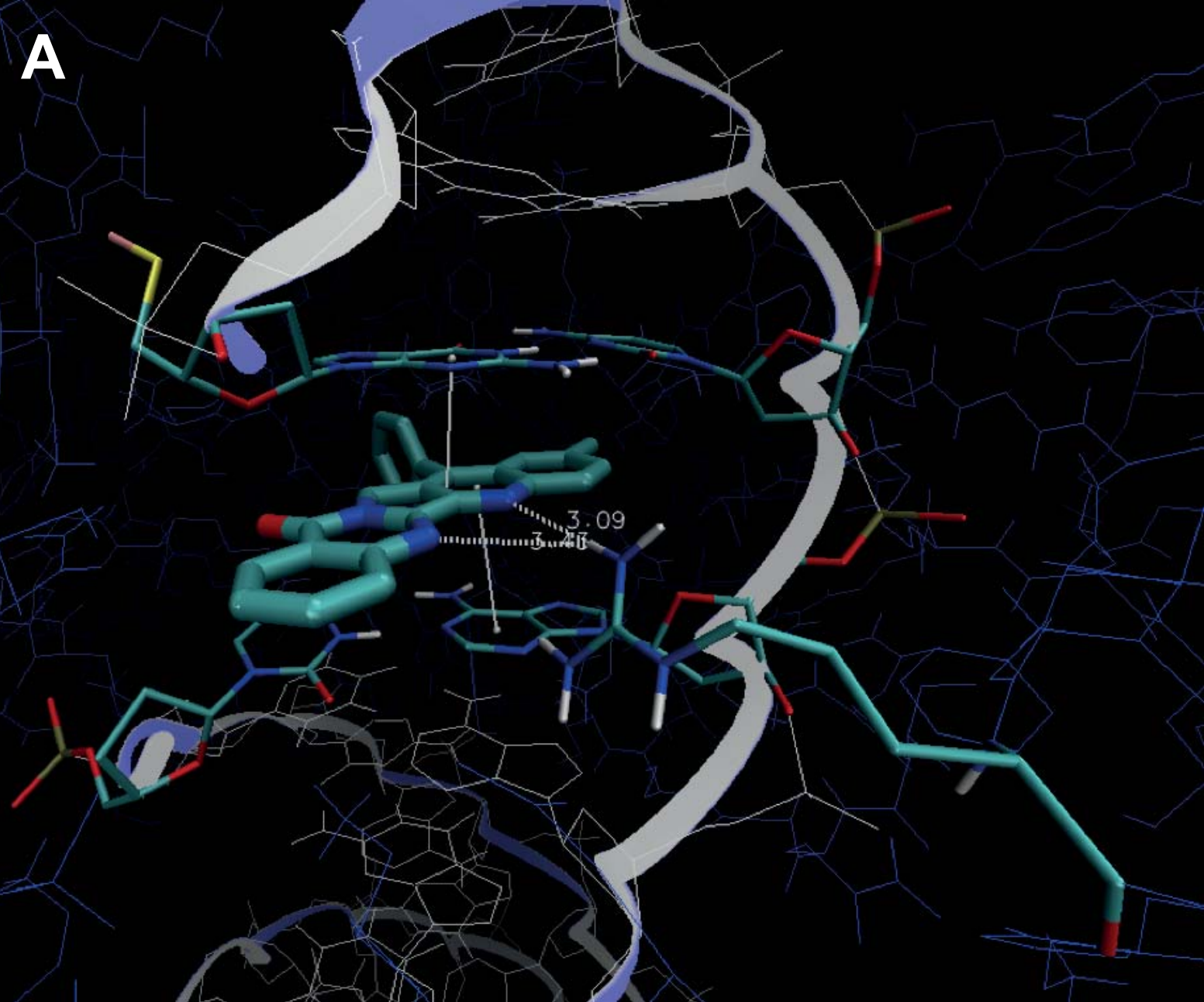**B**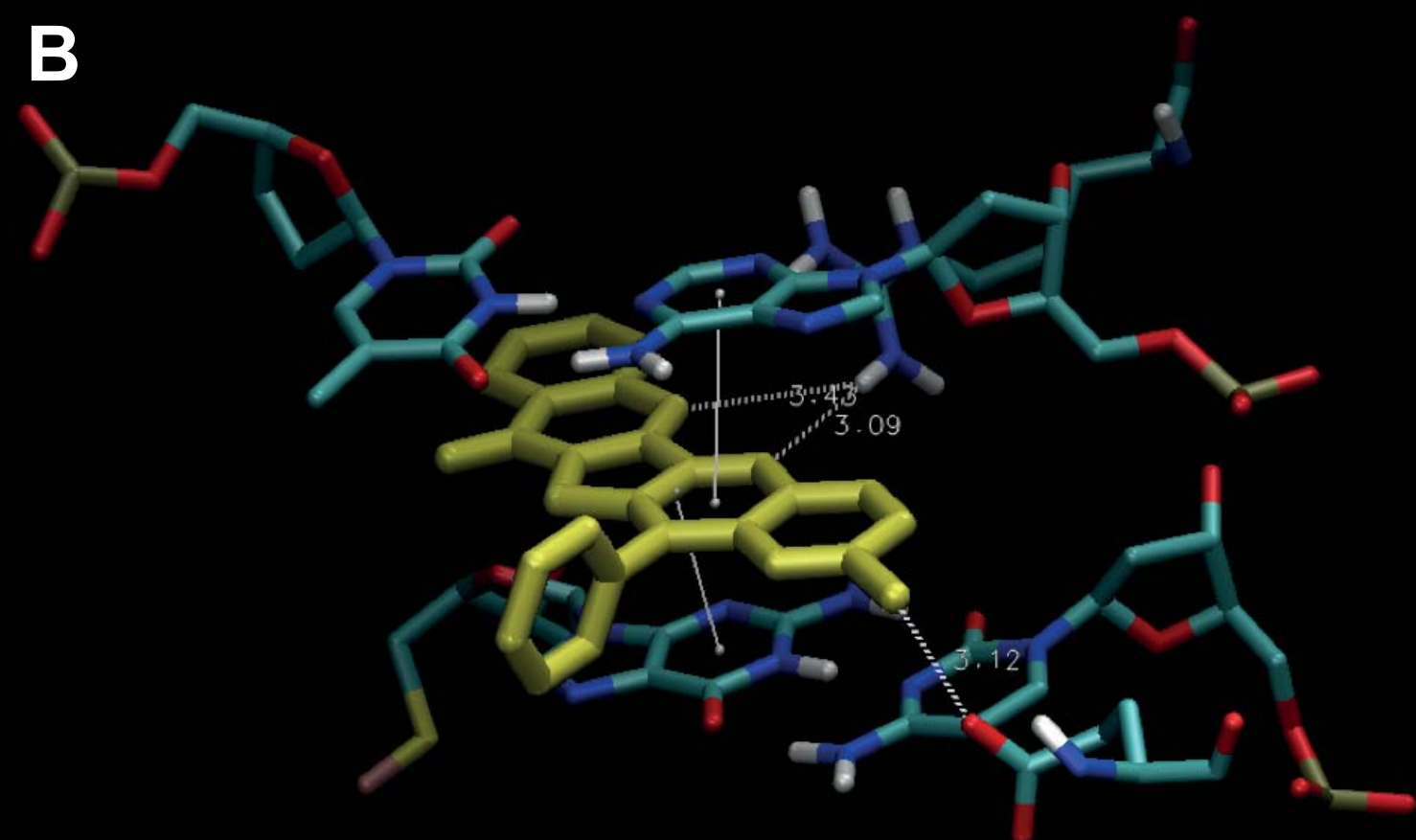

Supplement: File S1 — SI1, 1H and 13C–NMR spectra of all compounds. Figure S1, Two views of compound 3a (luotonin A) docked in the topoisomerase 1 active site. Figure S2, Two views of compound 3b docked in the topoisomerase 1 active site. Figure S3, Two views of compound 3c docked in the topoisomerase 1 active site. Figure S4, Two views of compound 3d docked in the topoisomerase 1 active site. Figure S5, Two views of compound 3e docked in the topoisomerase 1 active site. Figure S6, Two views of compound 3f docked in the topoisomerase 1 active site. Figure S7, Two views of compound 3g docked in the topoisomerase 1 active site. (ZIP) [file pone.0095998.s001.zip › Supporting info/FigureS4.pdf]

**A**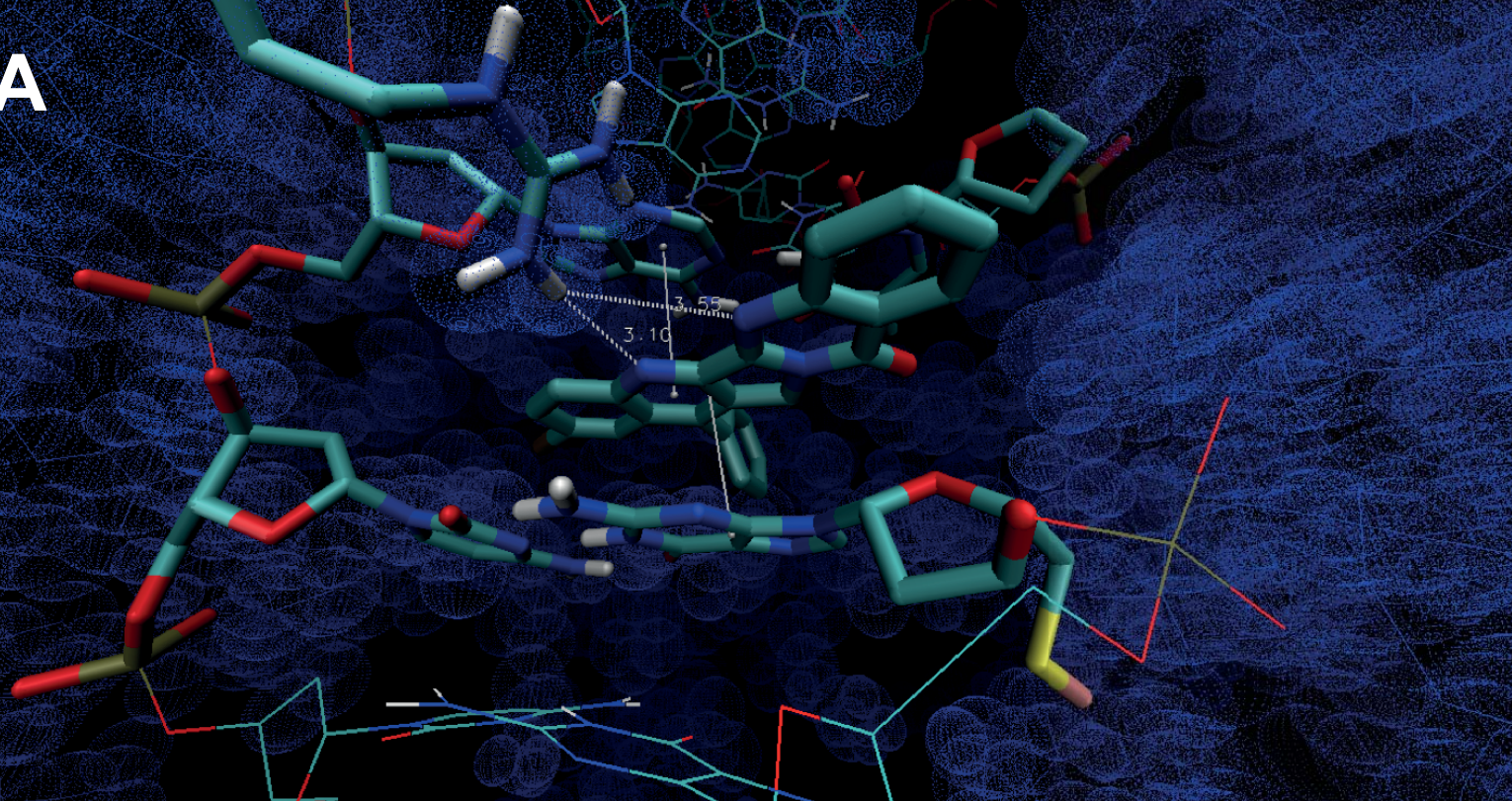**B**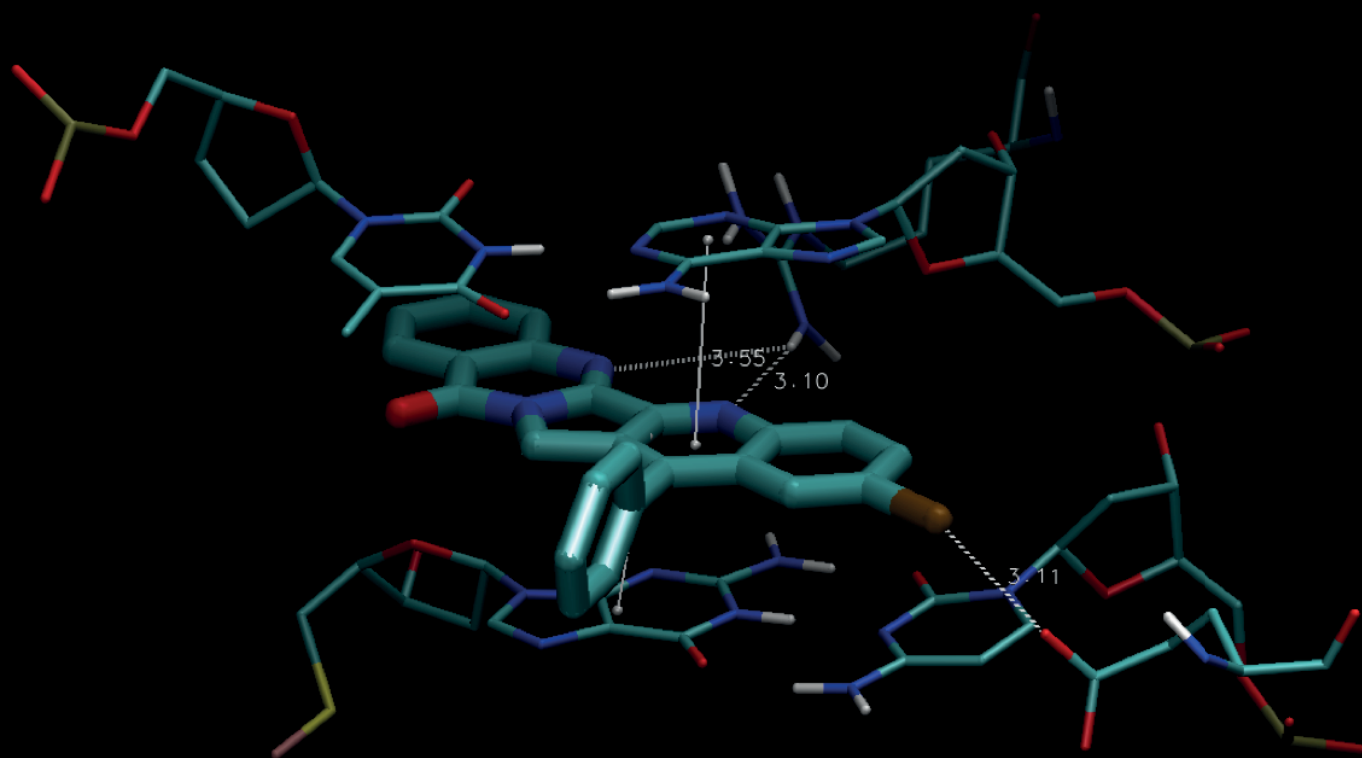

Supplement: File S1 — SI1, 1H and 13C–NMR spectra of all compounds. Figure S1, Two views of compound 3a (luotonin A) docked in the topoisomerase 1 active site. Figure S2, Two views of compound 3b docked in the topoisomerase 1 active site. Figure S3, Two views of compound 3c docked in the topoisomerase 1 active site. Figure S4, Two views of compound 3d docked in the topoisomerase 1 active site. Figure S5, Two views of compound 3e docked in the topoisomerase 1 active site. Figure S6, Two views of compound 3f docked in the topoisomerase 1 active site. Figure S7, Two views of compound 3g docked in the topoisomerase 1 active site. (ZIP) [file pone.0095998.s001.zip › Supporting info/FigureS5.pdf]

**A**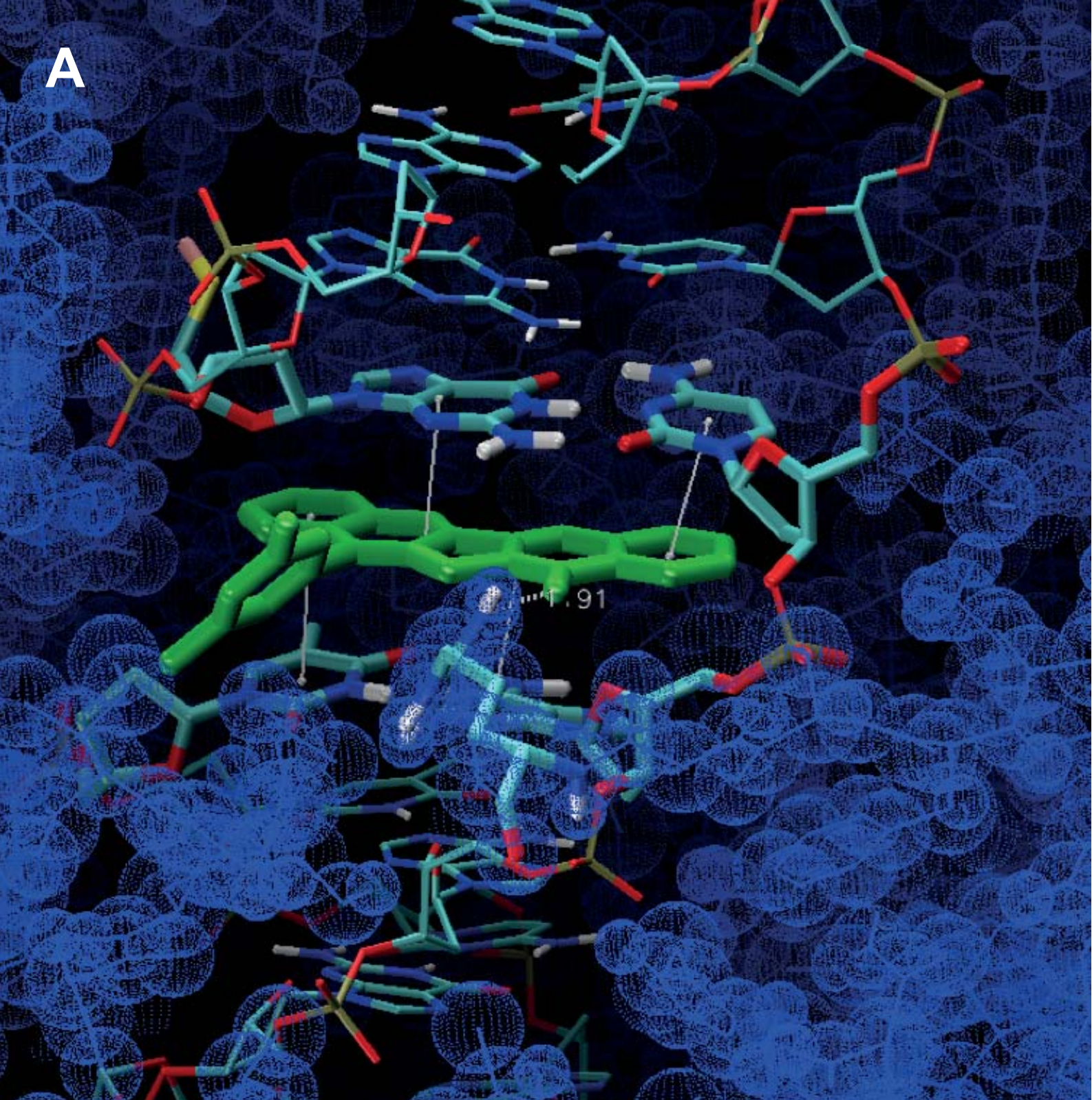**B**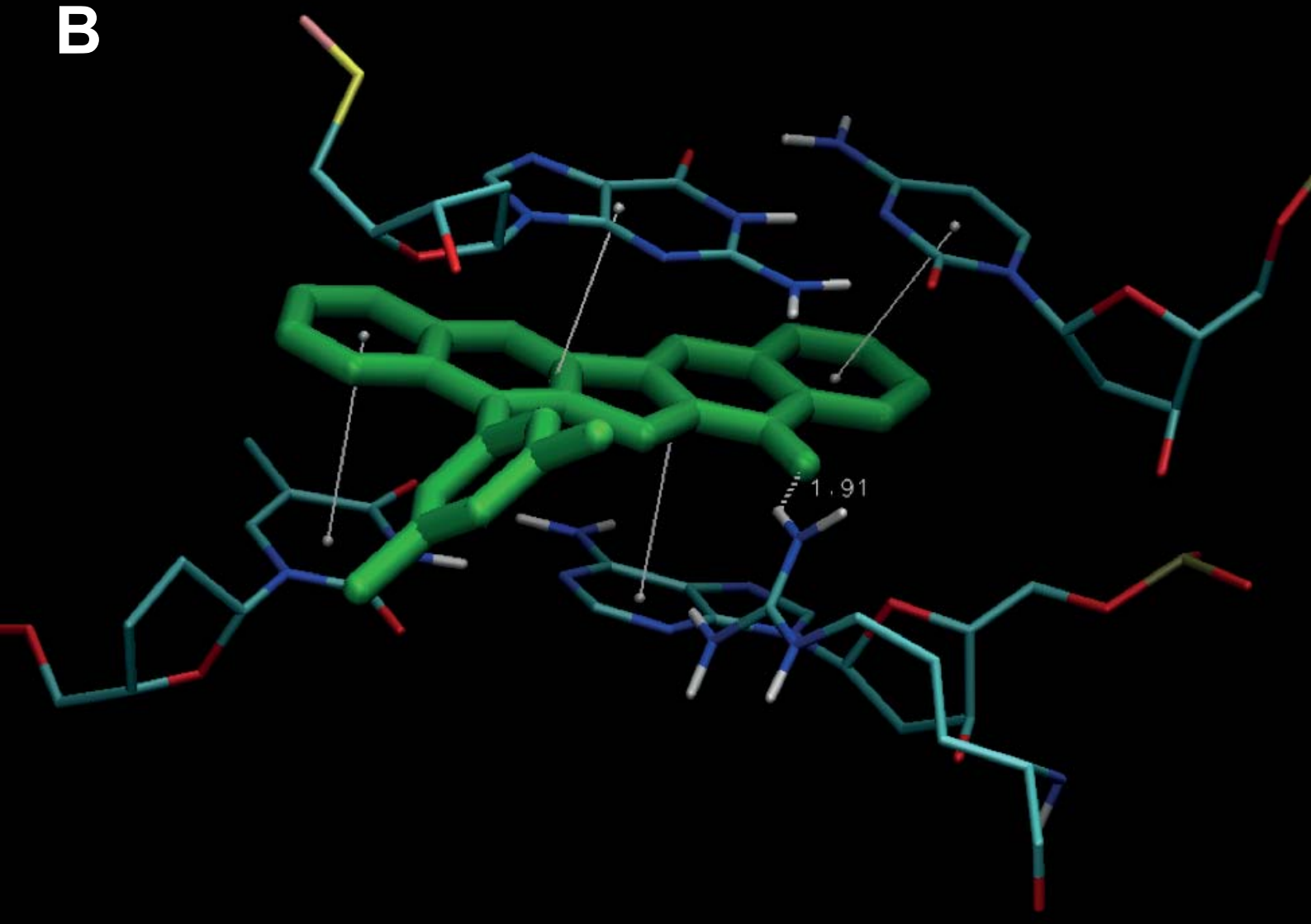

Supplement: File S1 — SI1, 1H and 13C–NMR spectra of all compounds. Figure S1, Two views of compound 3a (luotonin A) docked in the topoisomerase 1 active site. Figure S2, Two views of compound 3b docked in the topoisomerase 1 active site. Figure S3, Two views of compound 3c docked in the topoisomerase 1 active site. Figure S4, Two views of compound 3d docked in the topoisomerase 1 active site. Figure S5, Two views of compound 3e docked in the topoisomerase 1 active site. Figure S6, Two views of compound 3f docked in the topoisomerase 1 active site. Figure S7, Two views of compound 3g docked in the topoisomerase 1 active site. (ZIP) [file pone.0095998.s001.zip › Supporting info/FigureS6.pdf]

**A**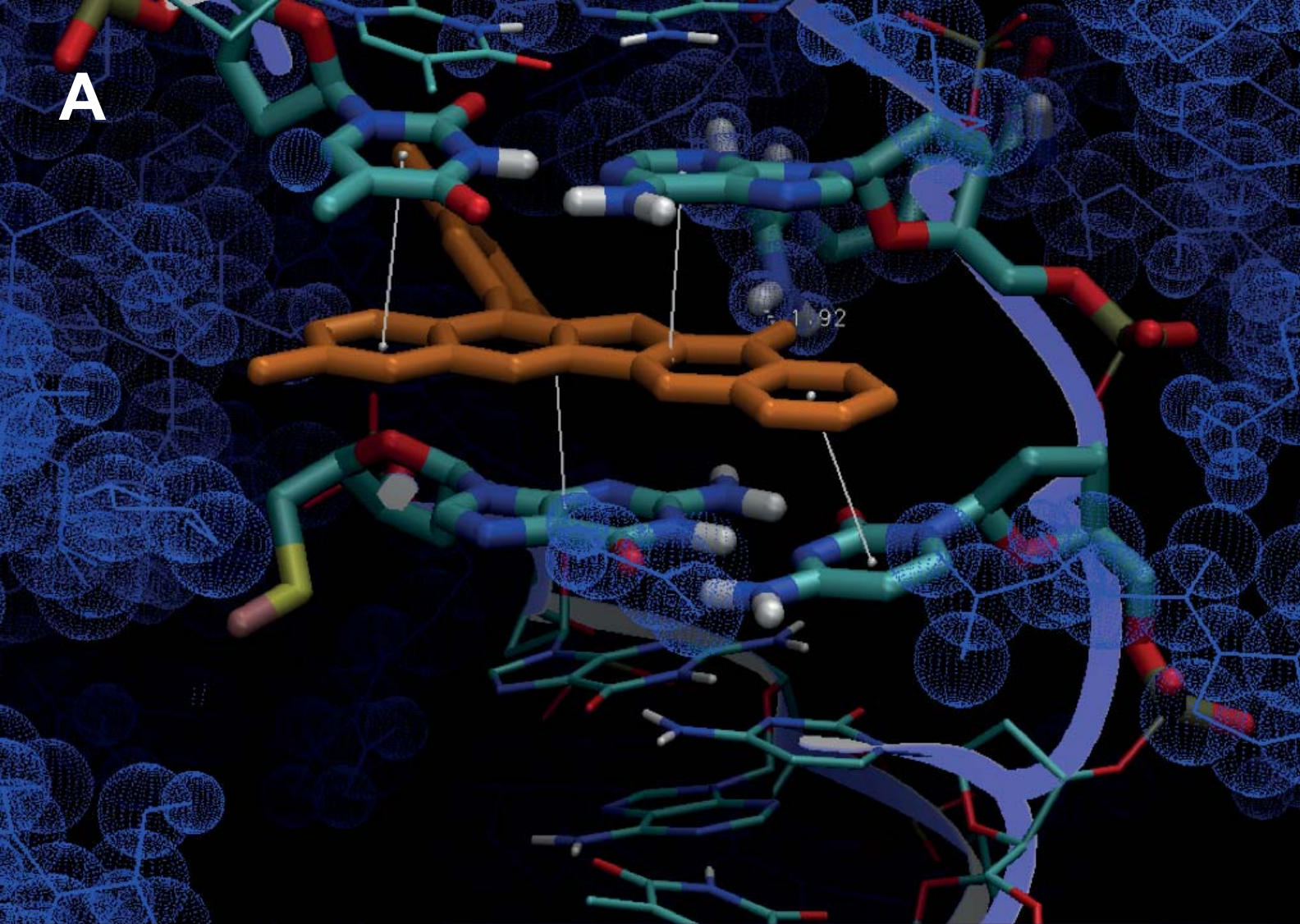**B**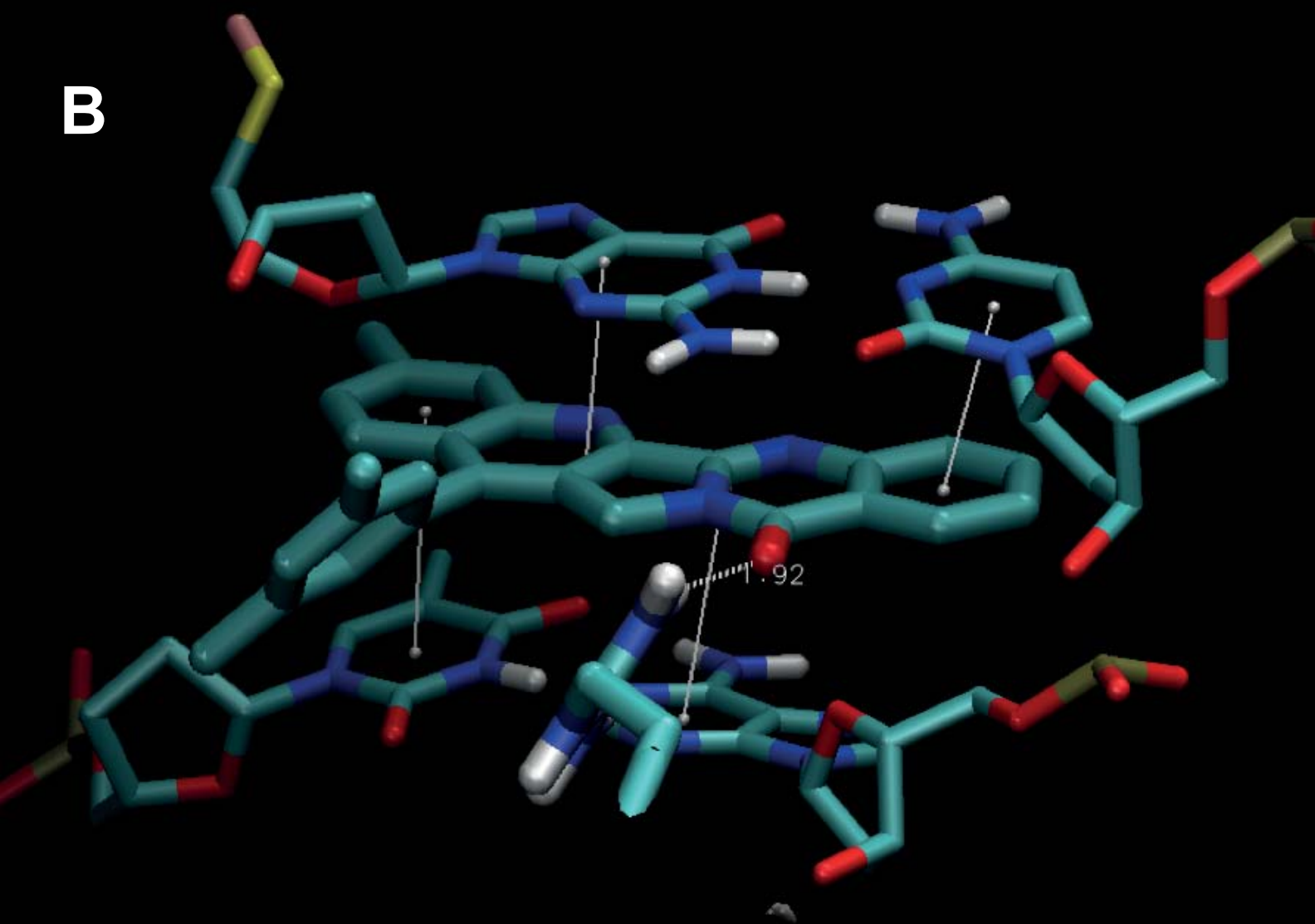

Supplement: File S1 — SI1, 1H and 13C–NMR spectra of all compounds. Figure S1, Two views of compound 3a (luotonin A) docked in the topoisomerase 1 active site. Figure S2, Two views of compound 3b docked in the topoisomerase 1 active site. Figure S3, Two views of compound 3c docked in the topoisomerase 1 active site. Figure S4, Two views of compound 3d docked in the topoisomerase 1 active site. Figure S5, Two views of compound 3e docked in the topoisomerase 1 active site. Figure S6, Two views of compound 3f docked in the topoisomerase 1 active site. Figure S7, Two views of compound 3g docked in the topoisomerase 1 active site. (ZIP) [file pone.0095998.s001.zip › Supporting info/FigureS7.pdf]
